# The effect of weather and climate on dengue outbreak risk in Peru, 2000-2018: A time-series analysis

**DOI:** 10.1371/journal.pntd.0010479

**Published:** 2022-06-30

**Authors:** Tia Dostal, Julianne Meisner, César Munayco, Patricia J. García, César Cárcamo, Jose Enrique Pérez Lu, Cory Morin, Lauren Frisbie, Peter M. Rabinowitz

**Affiliations:** 1 Center for One Health Research, Department of Environmental and Occupational Health Sciences, University of Washington, Seattle, Washington, United States of America; 2 Department of Epidemiology, University of Washington, Seattle, Washington, United States of America; 3 Centro Nacional de Epidemiología, Prevención y Control de Enfermedades, Peruvian Ministry of Health, Lima, Peru; 4 School of Public Health and Administration, Universidad Peruana Cayetano Heredia, Lima, Peru; 5 Center for Health and the Global Environment, Department of Environmental and Occupational Health Sciences, University of Washington, Seattle, Washington, United States of America; University of Colombo Faculty of Medicine, SRI LANKA

## Abstract

**Background:**

Dengue fever is the most common arboviral disease in humans, with an estimated 50-100 million annual infections worldwide. Dengue fever cases have increased substantially in the past four decades, driven largely by anthropogenic factors including climate change. More than half the population of Peru is at risk of dengue infection and due to its geography, Peru is also particularly sensitive to the effects of El Niño Southern Oscillation (ENSO). Determining the effect of ENSO on the risk for dengue outbreaks is of particular public health relevance and may also be applicable to other *Aedes*-vectored viruses.

**Methods:**

We conducted a time-series analysis at the level of the district-month, using surveillance data collected from January 2000 to September 2018 from all districts with a mean elevation suitable to survival of the mosquito vector (<2,500m), and ENSO and weather data from publicly-available datasets maintained by national and international agencies. We took a Bayesian hierarchical modeling approach to address correlation in space, and B-splines with four knots per year to address correlation in time. We furthermore conducted subgroup analyses by season and natural region.

**Results:**

We detected a positive and significant effect of temperature (°C, RR 1.14, 95% CI 1.13, 1.15, adjusted for precipitation) and ENSO (ICEN index: RR 1.17, 95% CI 1.15, 1.20; ONI index: RR 1.04, 95% CI 1.02, 1.07) on outbreak risk, but no evidence of a strong effect for precipitation after adjustment for temperature. Both natural region and season were found to be significant effect modifiers of the ENSO-dengue effect, with the effect of ENSO being stronger in the summer and the Selva Alta and Costa regions, compared with winter and Selva Baja and Sierra regions.

**Conclusions:**

Our results provide strong evidence that temperature and ENSO have significant effects on dengue outbreaks in Peru, however these results interact with region and season, and are stronger for local ENSO impacts than remote ENSO impacts. These findings support optimization of a dengue early warning system based on local weather and climate monitoring, including where and when to deploy such a system and parameterization of ENSO events, and provide high-precision effect estimates for future climate and dengue modeling efforts.

## Author summary

The theoretical importance of the El Niño Southern Oscillation to infectious disease transmission is widely accepted, however few studies have quantified this effect or its interaction with local environment. Using surveillance data on outbreaks of dengue fever in Peru, we have found evidence that El Niño events increase the risk of dengue outbreaks in the high jungle and coast, and decrease this risk in the low jungle. Our findings are likely generalizable to other viruses which are, like dengue virus, are transmitted by *Aedes aegypti* mosquitoes, including Zika, chikungunya, and yellow fever. As climate change is expected to increase the frequency of El Niño events, these results indicate that arbovirus outbreaks may also increase, and that El Niño events may be leveraged to predict them.

## Introduction

Dengue fever is one of the most important arboviral diseases worldwide, with significant socioeconomic and health impacts in the tropics and subtropics. In 2019, the World Health Organization (WHO) included dengue as one of the top ten threats to global health due to its increasing incidence worldwide and its spread into temperate climates [1]. While the global burden of dengue is unknown, cases are increasing rapidly with an 8-fold increase in reported cases over the last two decades [2]. In the Americas, the highest incidence of dengue cases occurred in 2019, with over 3 million infections reported [3].

The relationship between weather and the seasonal and inter-annual variation in dengue cases has long been a focus of dengue research. Dengue is endemic in some regions of Peru and the Peruvian Ministry of Health considers dengue to be an important re-emerging disease [4, 5]: in 2019, there were 17,143 reported cases of dengue with a cumulative national incidence rate of 53 cases per 100,000 people [6]. Currently, more than half of the population of Peru is at risk of dengue infection, with 17 of the 25 departments believed to be inhabited by *Ae. aegypti* mosquitoes [7, 8]. Environmental conditions, including weather, have a well-documented role in the survival, behavior, and proliferation of the mosquito vector (*Aedes aegypti* and *Aedes albopictus*), with warmer temperatures documented to increase mosquito feeding behavior, reduce gonotrophic cycle length, and hasten larval development [9]. Temperature also has a direct effect on viral replication in the mosquito midgut, with higher temperatures associated with a shorter extrinsic incubation period [10]. *Aedes* mosquitoes are container breeders, readily ovipositing in manmade containers such as water storage containers and discarded trash. Increases in rainfall can create new water-filled breeding sites in both natural and artificial containers, while drought may also increase breeding sites as households store water [11, 12].

The El Niño Southern Oscillation (ENSO) is considered to be one of the Earth’s most significant weather-producing phenomena, in which fluctuating ocean temperatures, trade wind strength, and atmospheric pressure lead to far-reaching climate condition changes [13–15]. El Niño and La Niña events generally occur every two to seven years in an irregular cycle with intervening neutral periods [16, 17]. El Niño warming events and La Niña cooling events can lead to widespread impacts on local weather, altering regional rainfall, temperature, and sunlight availability through atmospheric teleconnections [17].

Theoretically, the temperature and precipitation changes that appear with ENSO could affect dengue transmission by changing mosquito population and viral replication dynamics. The relationship between ENSO and dengue has become more relevant with an increasing ability to reliably forecast El Niño and La Niña events. In the future, such forecasts could form the basis of an early warning system for arboviral diseases, including dengue, and facilitate outbreak preparedness and targeting of interventions [18, 19].

Peru is particularly sensitive to ENSO due to its long coastline on the Pacific Ocean, the Andes mountains, and its low tropical rainforest regions [20]. This diversity of environmental conditions provides a unique opportunity to study interactions with local environment and the ENSO-dengue relationship. With the Andes mountain range running north to south across Peru, the country can be divided into three natural or “eco” regions: the Costa (coast), a narrow strip of land between the Pacific Ocean and the western Andes; the Sierra (mountains), encompassing the Andes mountain range; and the Selva (jungle), which covers the majority of Peru and is further divided into the high jungle (Selva Alta) located in the eastern foothills of the Andes, and the low jungle (Selva Baja), part of the Amazon River basin. The local impacts of El Niño and La Niña vary between regions and events, but studies have found that strong El Niño events can bring heavy rainfall to the Costa north of Lima and areas north of the Pacific hydrographic drainage, while also causing decreased rainfall in the Lake Titicaca hydrographic drainage and southern Andean region [20, 21]. In addition to changes in rainfall, El Niño events can cause increased temperatures and heat waves, particularly along the coast [21]. While previous studies have detected associations between local weather variables and dengue epidemics in Peru [22, 23], prior efforts to estimate the association with El Niño events have been largely descriptive [24] and limited in geographic scope [24, 25].

Despite the wealth of literature on the meteorological drivers of dengue outbreaks and the theoretical importance of ENSO events, literature on the relationship between ENSO and outbreaks of arboviruses, and modification of this effect by season or local environment, is limited. We utilized a hierarchical Bayesian approach and 18 years of dengue surveillance to characterize the association between ENSO effects and dengue risk in Peru at the district level. This work fills a knowledge gap important to mitigating the effects of dengue in Peru by describing the utility of local weather data and El Niño events in forecasting dengue epidemics.

## Methods

R version 3.5.3 [26] was used for all analyses.

### Setting

**Fig 1:**
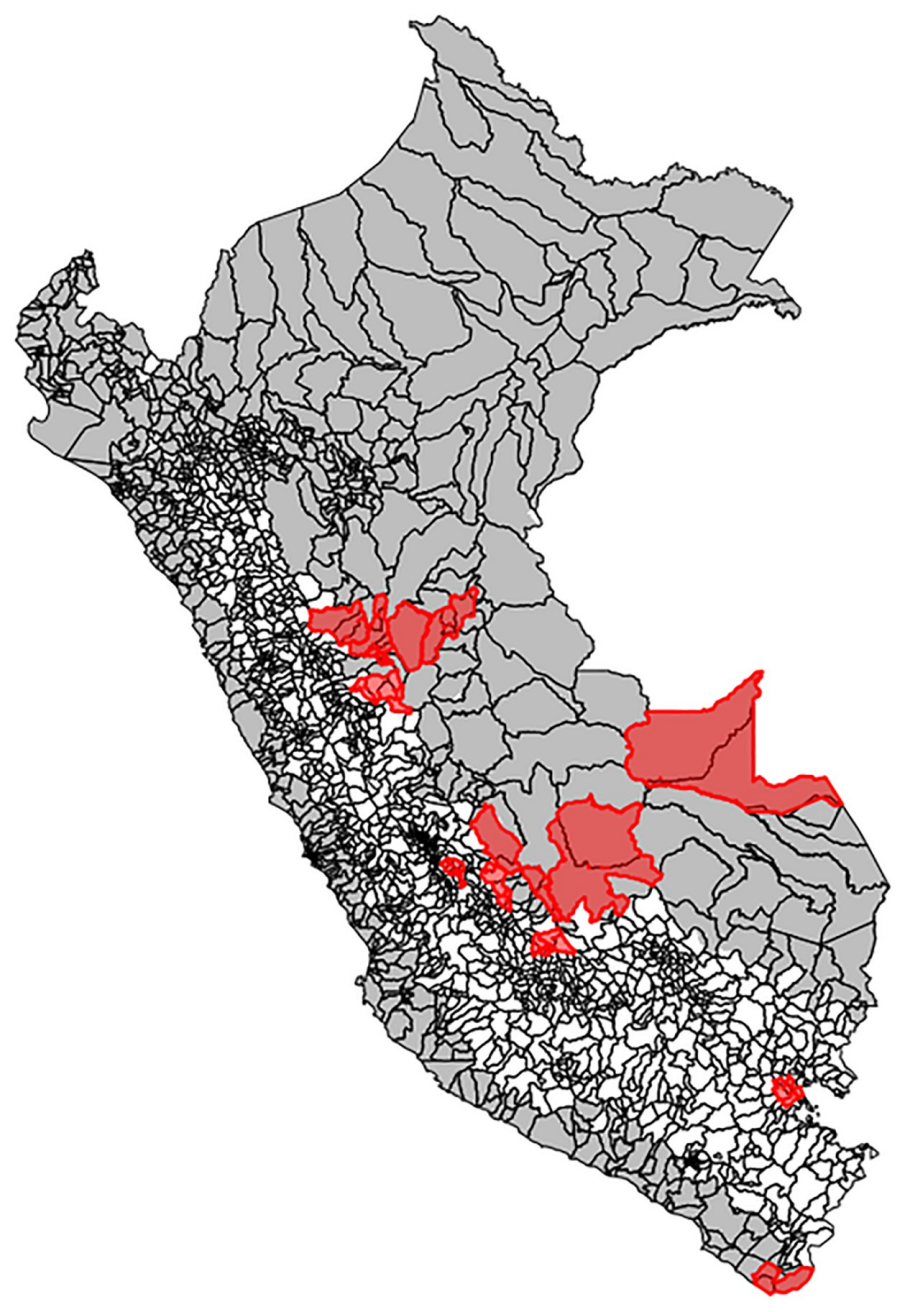
Study districts across Peru (grey). Higher elevation districts (white) excluded from analyses. Merged districts (red) were created by collapsing districts formed during the study period with their parent district(s). Base map source: GEOSS Information Exchange Data Hub, archived.

The study area includes all districts in Peru with a mean elevation of less than 2,500 meters, over the period January 2000 to September 2018. There are 1,851 total districts in Peru; as some districts were formed during the study period, and novel districts would not be contributing to reporting prior to their inception, we considered these newly-formed districts as part of their parent districts to mitigate missingness in the outcome variable. This resulted in a total of 1,834 districts. After eliminating districts with a mean elevation of more than 2,500 meters, an altitude at which the *Ae. aegypti* vector is unlikely to be present [27], 701 districts remained for analysis (Fig 1). We did not exclude district-months with low mean temperatures (i.e., temperatures significantly below the survival threshold of *Ae. aegypti*) as altitude thresholding eliminated nearly all such district-months; in the final analysis dataset, less than 1% of observations fell below 10°C.

### Study design

This study was a time series study, analyzed at the level of the district-month. This level of aggregation was dictated by data availability, with disease surveillance data available at the district-level and finest ENSO and environmental data available at the monthly-level.

#### Exposure

Temperature was parameterized as mean value in Celsius, and precipitation as total kg/m^2^ over an average month (30.33 days). These data were acquired from the NASA GLDAS, a global repository of moderate-resolution (0.25°) data derived from ground and satellite observations and land surface models [28, 29]. These data were downloaded from the NASA EARTHDATA website [30] and averaged over district in QGIS version 3.6 using the zonal statistics tool [31]. For districts smaller than the pixel size in the GLDAS data (n = 25), a 0.125° buffer was used. For aggregated districts and months following the creation of the novel district(s), mean temperature and precipitation for a given month were taken over the parent and novel districts.

ENSO events were parameterized as binary variables, with two indices used to reflect both remote and local ENSO effects in separate models. Remote impacts can cause weather change via atmospheric teleconnections, while local impacts can cause coastal rain and marine ecosystem disturbances. For the remote impacts we used monthly Oceanic Niño Index (ONI) data for the Niño 3.4 region, downloaded from the National Oceanic and Atmospheric Administration (NOAA) website [32]. For the local impacts we used monthly El Niño Coastal Index (ICEN) data, developed by Peru’s Estudio Nacional del Fenómeno El Niño, which we downloaded from the Instituto del Mar del Peru website [33]. Months with a sea surface temperature anomaly of greater than 0.5°C were classified as El Niño for the Niño 3.4 index, and months with sea surface temperature anomaly of greater than 0.4°C were classified as El Niño for the Niño ICEN index, in keeping with the respective agency’s definition of (at minimum) weak El Niño.

#### Outcome

Dengue cases were collected from Peru’s national dengue surveillance system, which is coordinated by the Centro Nacional de Epidemiología, Peruvian Ministry of Health. These data classify cases by diagnosis (with and without alarm signs and serious dengue), and type (probable, confirmed, and discarded), and include district of residence, age, sex, date of initial symptoms, date of diagnosis, and date of notification. We collapsed probable and confirmed cases for all analyses, where probable cases were defined by history of recent fever in addition to two or more symptoms (headache, retro-orbital pain, myalgia, joint pain, rash, or hemmorhagic condition), and confirmed cases were defined by meeting the probable case definition in addition to either (1) serum isolation, or (2) four-fold change in IgM or IgG titer, or (3) positive PCR, or (4) positive immunoassay, or (5) epidemiological link (residing in an area with laboratory-confirmed transmission and presence of *Ae. aegypti* in the past 15 days). Discarded cases were ruled out via laboratory tests or absence of an epidemiological link [34]. While case definitions changed during the study period, all data used were updated to the current WHO definition. No analyses were dengue serotype-specific. Date was defined by date of symptom onset, and any district-week with no reported cases was assigned 0 cases.

Due to concerns surrounding surveillance fatigue (whereby districts experiencing a very high number of cases become overwhelmed and cease reporting) [35] leading to outcome misclassification, we parameterized outcome as dengue outbreaks. Date of initial symptoms was used to assign cases to calendar week. In keeping with prior literature, an outbreak was defined as five or more cases within three or more consecutive weeks, extending forward and backward in time until at least two weeks with no reported cases occurred [22]. District weeks were assigned as outbreak weeks if they occurred during an outbreak in that district, regardless of conditions in neighboring districts (i.e., if a given district experienced 3 cases in a two week period, and a neighboring district experienced 4 cases in the same three week period, none of the district weeks in question would be assigned as outbreak weeks). This approach ensures imported cases do not contribute to a district’s outcome measurement, but may miss small outbreaks spread across border communities.

To aggregate from district-weeks to district-months, we summed the number of outbreak-weeks in a given month.

#### Confounders

In order to be a confounder, a variable must have a causal relationship with the outcome, and either have a causal relationship with the exposure or share a common cause [36]. While there are many important causes of dengue outbreaks—human mobility, vector control efforts, wind speed, etc.—these variables are not causes of ENSO events, nor are they likely to share common causes, thus we did not identify any confounders for the ENSO models. As temperature and precipitation may share a common cause, temperature may be a confounder of the precipitation effect, and precipitation may be a confounder of the temperature effect. We handled this by fitting a single model for temperature and precipitation and interpreting the coefficient for temperature as adjusted for precipitation, and the coefficient for precipitation as adjusted for temperature.

#### Effect modifiers

We investigated season and eco-region as effect modifiers for the ENSO effect, defining effect modification by the presence of a statistically significant interaction term on the multiplicative scale.

Eco-region was hypothesized to be an effect modifier as topographical, ecological, and climactic features unique to each region may modify both the impact of ENSO events and mosquito population and dengue transmission dynamics. Eco-region was parameterized as Selva Alta, Selva Baja, Costa, and Sierra [37].

Eco-region data were downloaded from a Universidad de San Martín de Porres database [38]. We parameterized season as binary summer/winter, and defined summer as December to April and winter as May to November.

#### Statistical analyses

We fit all models as Poisson Bayesian hierarchical spatial smoothing models, with district-month as the unit of analysis and outcome parameterized as outbreak weeks in each district-month (the number of weeks, in that district and month, in which an outbreak occurred), using the INLA package version 19.09.03 [39]. We fit the BYM model, a standard model for estimation of ecological associations [40]. This model “absorbs” extra-Poisson variation into the structured and unstructured spatial random effects [41]:

$$\begin{array}{c} Y_{i}∼Poisson\left(\lambda_{i}\right) \end{array}$$ (1)

$$\begin{array}{c} \lambda_{i}=exp(\beta_{0}+\beta_{1}X+\sum_{k=1}^{K}b_{kt}\beta_{k}+e_{i}) \end{array}$$ (2)

$$\begin{array}{c} e_{i}=S_{i}+\epsilon_{i} \end{array}$$ (3)

$$\begin{array}{c} \epsilon_{i}|\sigma_{\epsilon }^{2}∼{}_{iid}N\left(0,\sigma_{\epsilon }^{2}\right) \end{array}$$ (4)

$$\begin{array}{c} S_{i}|S_{j},j\in ne\left(i\right)∼N({\bar{S}}_{j},\frac{\sigma_{s}^{2}}{m_{i}}) \end{array}$$ (5)

Where

- *i* indexes district (*i*=1,2,…,701) and *t* indexes time (*t*=1,2,…,225)
- *Y*_*i*_ is outbreak weeks in district *i*
- **X** is a vector of fixed effects
- Time trends are modeled with a cubic B-spline basis *b*_*kt*_ and *K* is such that we have 4 knots evenly spaced per year
- *ϵ*_*i*_ are the unstructured (non-spatial) random effects
- **S** is the vector of structured (spatial) random effects, assumed to follow the ICAR model
- *ne*(*i*) denotes neighbors (shared boundaries) of district *i*
- *m*_*i*_ is the number of neighbors of district *i*

For both random effects we used a “penalized complexity” prior [42, 43] *Pr*(*σ* > *U*) = *α*, where *σ* is the standard deviation for the structured and unstructured random effects, *U* = 1, and *α* = 0.01, giving the prior *Pr*(*σ* > 1) = 0.01. With a log link, this specification gives a 99% posterior credible interval of (0.31, 2.72), on the multiplicative scale, for each random effect’s residual relative risk. This specification was to be wide enough such that the prior would not have undue influence on the posterior.

Fixed effects were governed by the model in question, at minimum containing the exposure of interest (temperature, precipitation, El Niño ONI, El Niño ICEN), and at most extending to interaction terms between the exposure of interest and an effect modifier (season, eco-region).

#### Inference

We have used B-splines to address temporal autocorrelation rather than including temporal random effects to both mitigate the computational challenges of fitting models with 701 spatial random effects and 225 temporal random effects, and to retain interpretability of our effect estimates. If temporal random effects were included, effect estimates derived from regression coefficients would pertain to district-weeks with a temporal random effect of 0.

#### Model checking

In keeping with best practices for hypothesis testing and statistical inference, all hypotheses—and therefore fixed effects and their functional forms—were pre-specified and not subject to model testing or fitting. Thus, no variable selection occurred. However, partial autocorrelation functions and Moran’s I were applied to model residuals to check for residual temporal and spatial autocorrelation, respectively, to ensure our choices of splines in time, structured and unstructured random effects in space, and penalized complexity priors were adequate to address autocorrelation. Also consistent with these principles, results are only presented for exposures of interest as detailed above.

We implemented Moran’s I using the moran.test() function in the spdep package [44], defining neighbors by shared boundaries and autocorrelation as *p* ≤ 0.05. We computed partial autocorrelation functions using the pacf() function in the stats package [26], and inspected a sample of plots from randomly-selected districts to evaluate evidence of residual temporal autocorrelation.

## Results

### Descriptive statistics

**Fig 2:**
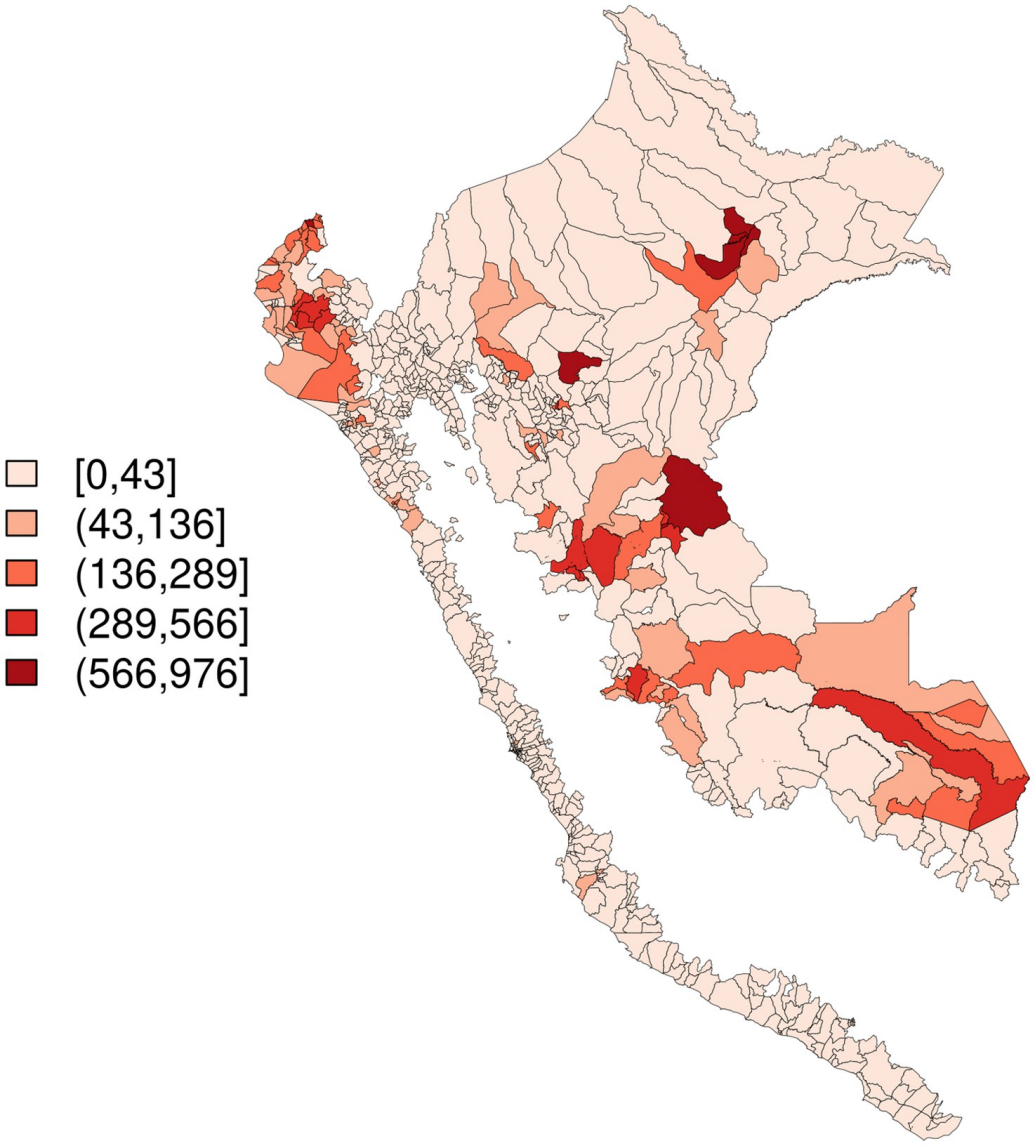
Outbreak weeks, summed over time, merged districts in Peru (n = 1,834), January 2000-September 2018. Base map source: GEOSS Information Exchange Data Hub, archived.

**Fig 3:**
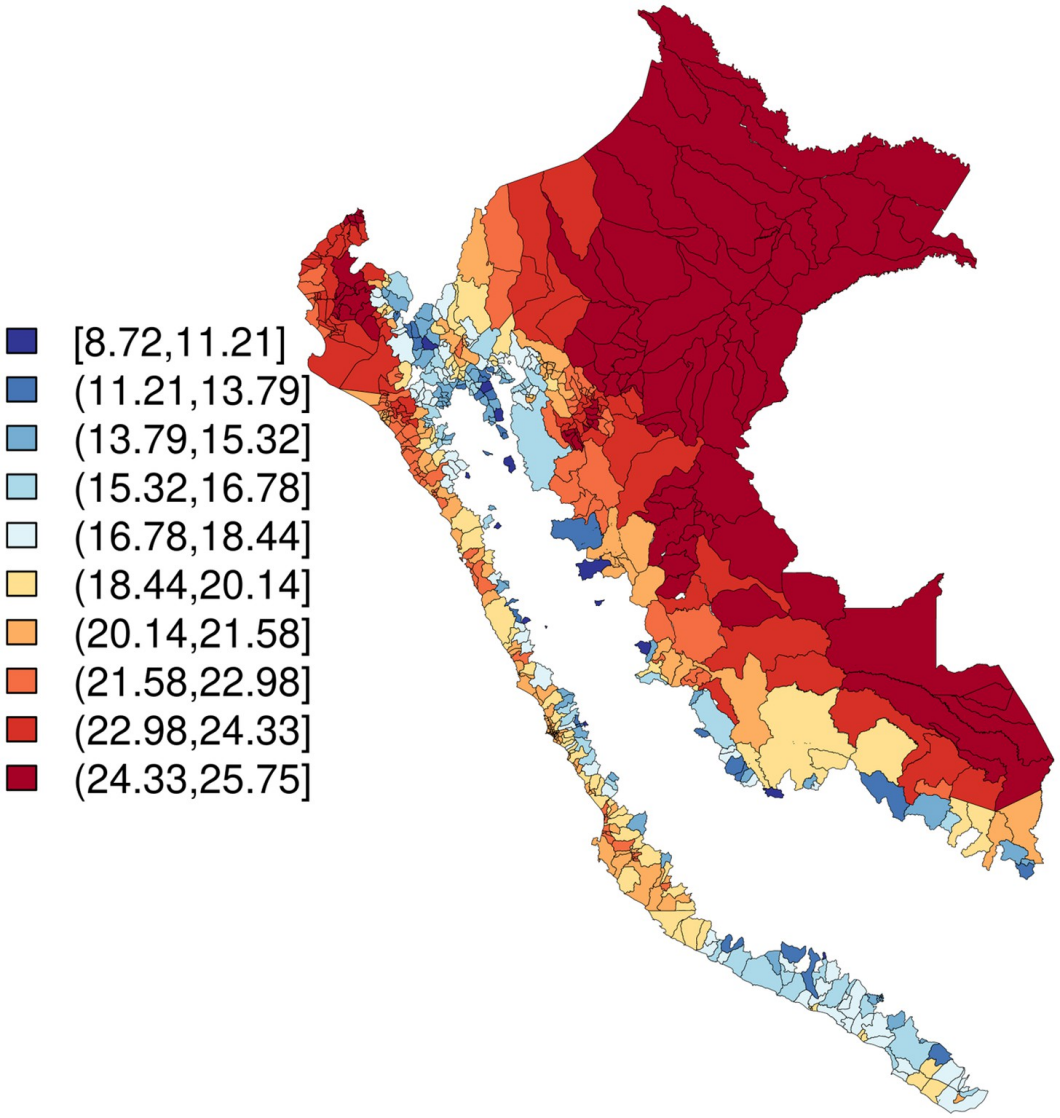
Temperature (mean over time; °C), merged districts in Peru (n = 1,834), January 2000-September 2018. Base map source: GEOSS Information Exchange Data Hub, archived.

**Fig 4:**
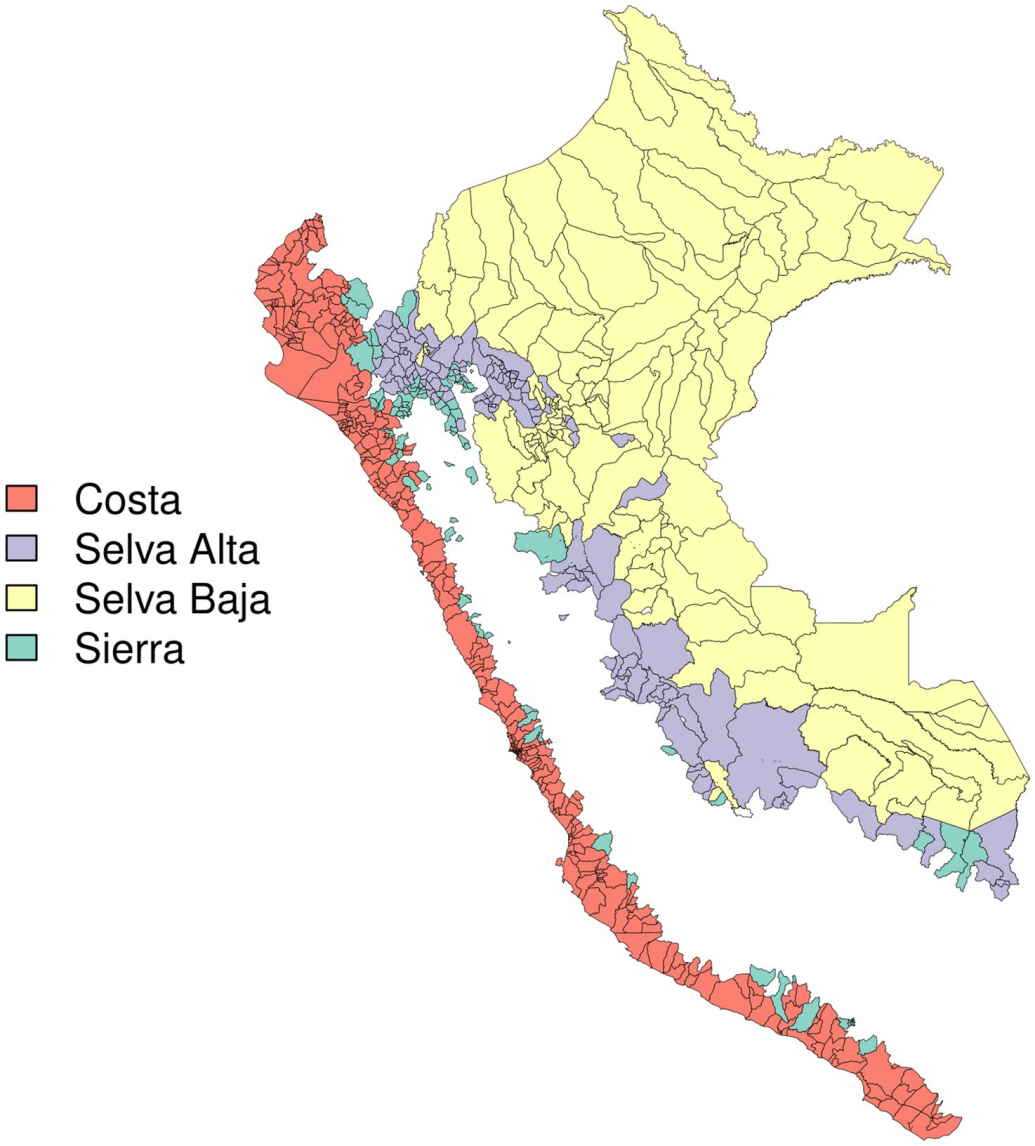
Natural region, merged districts in Peru (n = 1,834), January 2000-September 2018. Base map source: GEOSS Information Exchange Data Hub.

**Fig 5:**
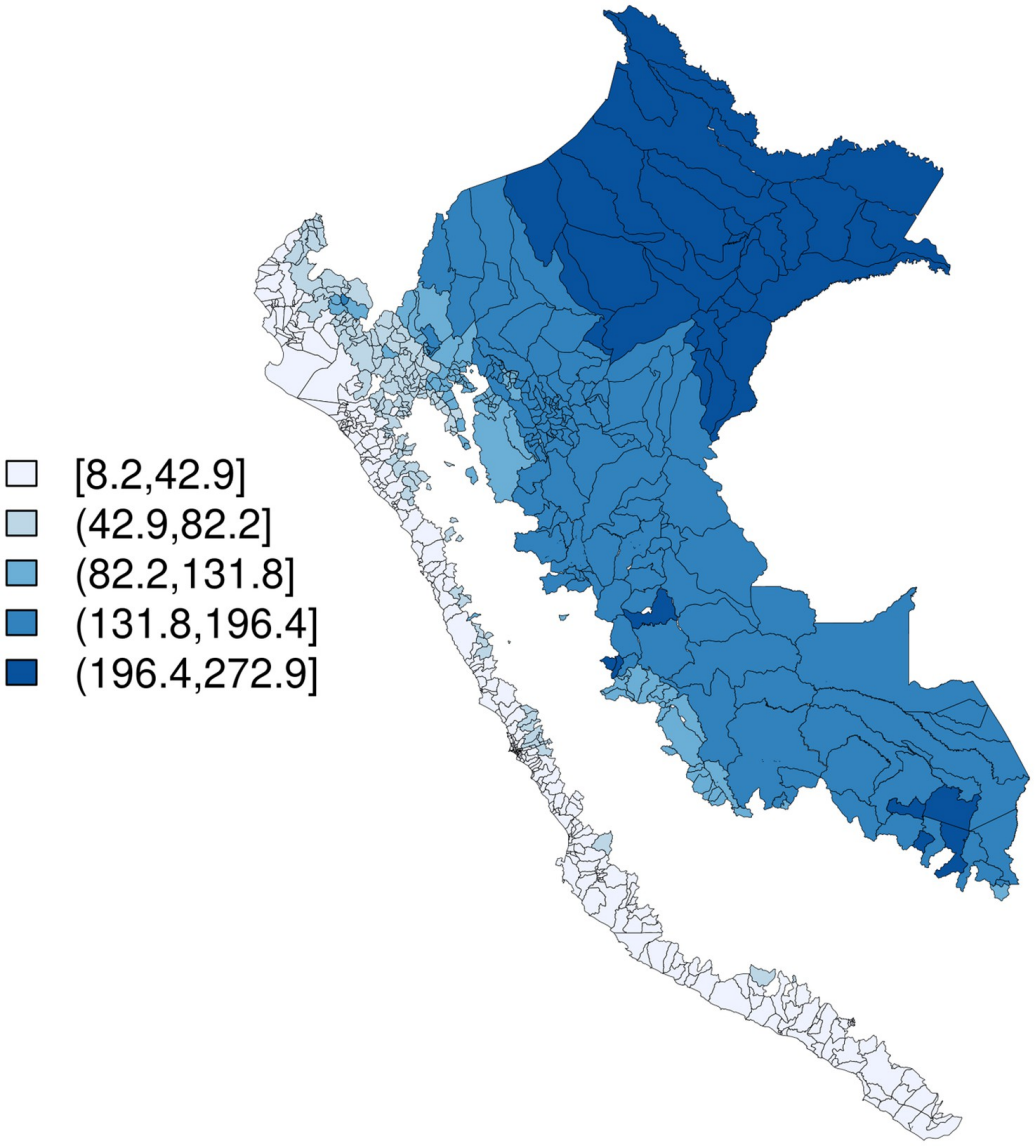
Precipitation (mean over time; kg/m^2^), merged districts in Peru (n = 1,834), January 2000-September 2018. Base map source: GEOSS Information Exchange Data Hub, archived.

**Fig 6:**
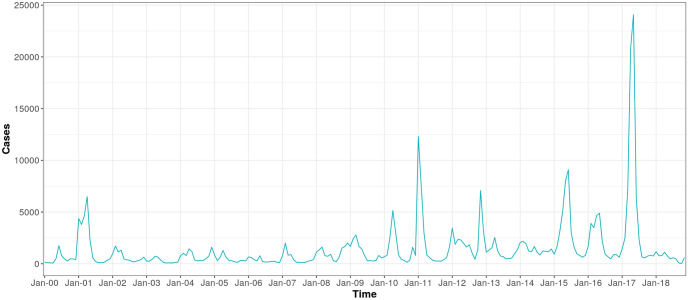
Dengue cases, summed over districts (n = 701), January 2000-September 2018.

**Fig 7:**
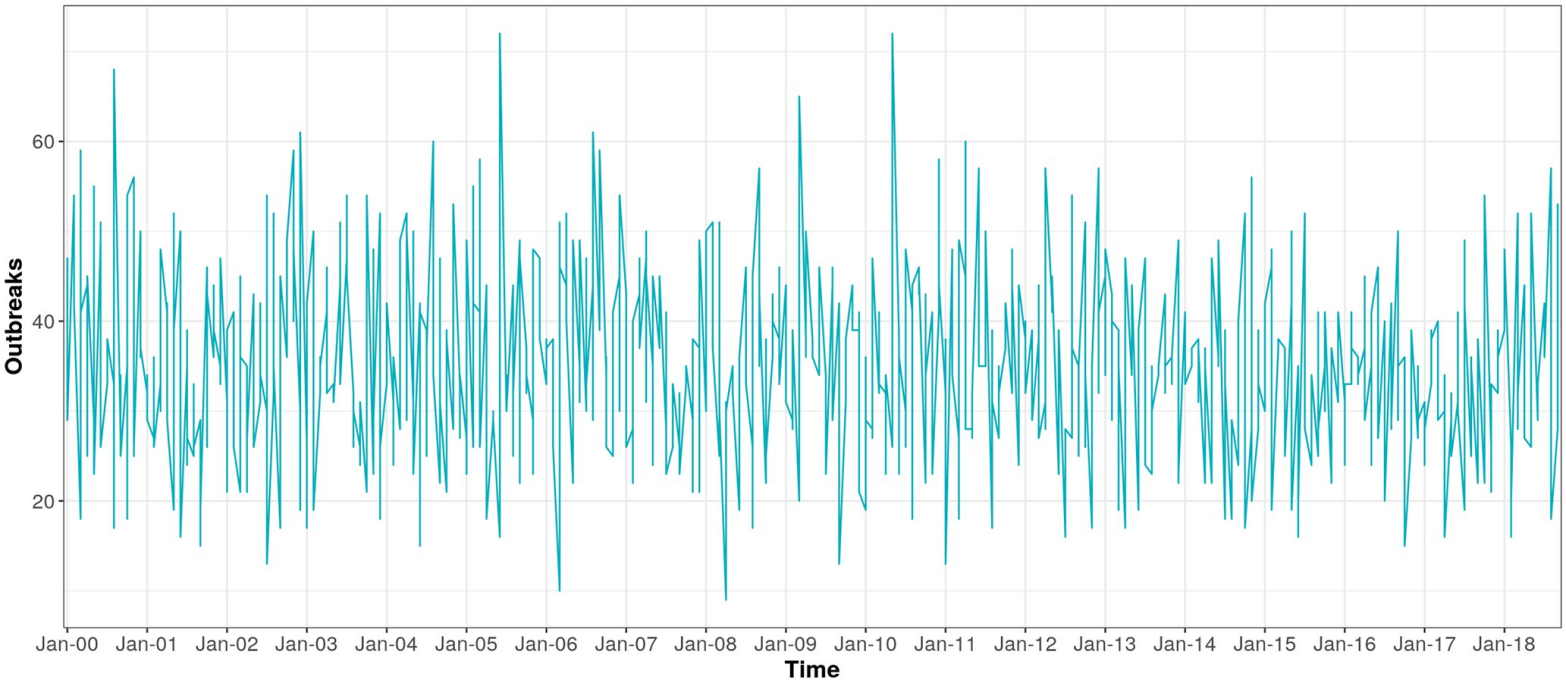
Number of outbreak weeks, summed over districts (n = 701), January 2000-September 2018.

An average of 0.16 outbreak weeks were observed across district-months, and 20–27% of months were El Niño months, depending on index used (Table 1). Districts with higher values for outbreak weeks appear to cluster together (Fig 2), temperature appears lowest in the Selva Baja and Selva Alta regions (Figs 3 and 4), and precipitation generally increases along a west to east gradient (Fig 5). The largest national outbreak during the study period occurred in 2017, with smaller outbreaks noted in 2011, 2013, and 2015, in order of decreasing size (peak cases) (Figs 6 and 7).

**Table 1:** Descriptive statistics after merging formed districts and eliminating districts with elevation >2,500 meters (n = 701) January 2000-September 2018. Mean was taken over district-months for outbreak weeks, temperature, and precipitation; over districts for region; and over months for El Niño variables.

Variable	Mean (sd)
Outbreak weeks	0.16 (0.78)
Temperature (°C)	20.04 (4.01)
Precipitation (kg/m^2^)	81.15 (90.12)
Natural region*	
Costa	341 (49%)
Selva Alta	129 (18%)
Selva Baja	137 (20%)
Sierra	93 (13%)
El Niño ONI*	46 (20%)
El_Niño ICEN*	60 (27%)

sd: standard deviation; ONI: Oceanic Niño index for Niño 3.4 region (remote impacts); ICEN: El Niño Coastal Index (local impacts).

*n(%)

### Regression models

Both natural region and season were statistically significant effect modifiers of the El Niño effect, thus only stratified results (on region or season) are presented (Table 2). All stratified effects were statistically significant; the effect of the ONI index was positive in the summer and negative in the winter, versus positive for both using the ICEN index, but stronger for summer (Table 2). Natural region results were also in general stronger and more positive for the ICEN index than the ONI index, with the exception of Selva Baja (ONI effect slightly stronger than ICEN). The effect for Sierra was on the opposite side of the null, being negative for the ONI index and strongly positive for the ICEN index (Table 2). Effect estimates were also statistically significant for temperature, with a one degree Celsius increase in temperature associated with a 14% higher rate of dengue outbreaks (95% CI 1.13, 1.15). There was no evidence of effect for precipitation (Table 2).

**Table 2:** Results from regression models, presented for each exposure of interest. Temperature and precipitation were fit in the same model, thus each was adjusted for the other; no adjustments were made in either El Niǹo model.

		RR (mean)	(95% CI)
Temperature		1.14	1.13, 1.15
Precipitation		1.00	1.00, 1.00
El Niño ONI	Summer	1.13	1.09, 1.17
	Winter	0.93	0.90, 0.96
	Selva Baja	0.90	0.86, 0.94
	Selva Alta	1.21	1.14, 1.29
	Costa	1.10	1.06, 1.13
	Sierra	0.83	0.74, 0.93
El Niño ICEN	Summer	1.76	1.71, 1.84
	Winter	1.45	1.40, 1.51
	Selva Baja (low jungle)	0.94	0.91, 0.98
	Selva Alta (high jungle)	1.58	1.48, 1.71
	Costa (coast)	1.93	1.86, 2.00
	Sierra (mountains)	2.44	1.06, 6.06

RR: rate ratio; CI: posterior credible interval; ONI: Oceanic Niño index for Niño 3.4 region; ICEN: El Niño Coastal Index.

#### Model checking

Partial autocorrelation functions for a random sample of 10 districts are presented in S1 Fig. There were no obvious temporal trends in the residuals, suggesting no residual autocorrelation in time. Similarly, Moran’s I tests produced p-values between 0.46 and 0.86 across these models, suggesting no residual autocorrelation in space. Taken together, these results support the validity of our models.

## Discussion

This study detected a significant positive effect of El Niño on dengue outbreaks in Peru, with the index of local impacts showing a stronger association than the index of remote impacts. We also found a significant positive effect of increasing temperature on dengue outbreaks in Peru.

Our results furthermore demonstrated marked effect modification by season and natural region for the ENSO-dengue relationship. In particular, ENSO events were associated with a lower risk of dengue outbreaks in Selva Baja, but a higher risk of outbreaks in Selva Alta and Costa, and this result was consistent across both ENSO indices (remote and local). For Sierra and for winter, however, results were disparate across these indices: remote ENSO effects were associated with a lower risk of dengue outbreaks, while local ENSO effects were associated with a higher risk. This result may reflect model overfitting due to the low number of outbreaks occurring in the winter and Sierra, with over 95% of district-months having no outbreaks in this season and region. However, there are several explanations for a true differential effect of ENSO on dengue outbreaks by natural region.

A 2011 study by Chowell et al. found that the timing of dengue epidemics differed significantly between natural regions (jungle, mountain, coastal) in Peru—with the jungle and mountain regions having an epidemic peak of dengue about six weeks prior to the peak in the coastal region [22]—and weather effects of ENSO events may also differ by natural region. Generally absent along the coast in non-ENSO years, rainfall increases up to 20-fold in some northern coastal regions during ENSO events [45], with similarly marked increases in rainfall in the northwestern Sierra region furthermore bringing even more water into rivers and streams located at lower altitudes [46]. Dengue transmission potential is known to be influenced by temperature-humidity coupling [23], and increases in river discharge and flooding subsequent to this heavy rainfall in the Sierra region create a favorable environment for vector breeding in both the Sierra and Costa regions. With regards to the jungle region, on the eastern slope of the Andes there is a temperature gradient from Selva Baja to Selva Alta [47]. During ENSO events, rising temperatures move the lower threshold for mosquito development to a higher altitude, allowing mosquitoes to breed in a larger area of the Selva Alta region [9]. Conversely, in Selva Baja, ENSO events lead to warmer temperatures and drier conditions which are less favorable for vector breeding [46, 48, 49].

Our study has several strengths. We used nearly twenty years of surveillance data corresponding to a sample size of nearly 160,000 district-months, and parameterized outcome as dengue outbreaks to mitigate the effects of reporting fatigue and measurement error arising from undetected asymptomatic cases. We also used a robust spatial model to handle spatial autocorrelation, using principled priors and a spatial random effect well-suited to a study region comprised largely of areas (districts) with many neighbors. We adopted a causal inference approach to identifying confounders, mitigating the risk of overadjustment bias. While prior authors have detected associations between local weather variables and dengue epidemics in Peru [22, 23], studies on the relationship with El Niño events have been limited to descriptive statistics in a single department each (Ica, a southern coastal department, and Huanuco, a central eastern department) [24, 25]. Our study extends these previous efforts by estimating the magnitude of the ENSO effect on dengue outbreaks across all districts in Peru, facilitating estimation of effect modification by natural region and improving the statistical precision of our effect estimates.

However, our study also has several limitations. Namely, use of surveillance data is subject to bias arising from health-seeking behavior, successful identification of dengue cases by healthcare providers, and intact surveillance infrastructure. We assumed any district-months not appearing in the surveillance data had 0 cases, however it is possible such units experienced unrecognized or unreported dengue cases or even outbreaks. If such underreporting is random in space and time we expect our effect estimates to be attenuated (closer to 1), however if there is a spatial or temporal pattern to this underreporting, unpredictable bias can result. For instance, during the 2017 El Niño costero event, 64 healthcare centers were destroyed and 1,044 were damaged; this event may have precipitated both dengue transmission and underreporting [50]. Furthermore, our choice of month as the temporal scale of analysis, dictated by granularity of the climate data, may not be the appropriate timescale to capture the effect of local weather and ENSO events on dengue outbreak risk.

We did not adjust for dengue prevention activities during the study period as we did not have access to these data; Peru has not implemented dengue vaccination at this time. Prevention measures other than vaccination may represent effect modifiers if ENSO or weather effects are different in their presence versus absence, in which case effect estimates should be stratified on intervention status.

Our study is furthermore an ecological study, conducted at the level of the district-month. While the exposures of interest are group-level without inherent individual-level analogs, cross-level bias may nevertheless arise from individual-level effect modifiers whose distribution or effect differs across districts or time [51]. For instance, if individual-level variables such as access to air conditioning modifies the effect of ENSO events on dengue risk, and the distribution of these variables is different in different district-months, bias will result. However, an individual- or multi-level study on the scale of this analysis would not be feasible, and wealth data would not rectify the limitations of an ecological design.

Though rainfall affects mosquito populations via breeding site creation, our study did not find a positive association between precipitation and dengue outbreaks. This result could be due to a highly local effect of precipitation that is no longer appreciable at the district-level, or failure of our models to capture the complex and non-linear relationship between precipitation and mosquito ecology. While reduced precipitation limits reproductive habitat in theory, during times of drought people may store water and inadvertently create suitable habitat for mosquitoes near their homes [12]. Furthermore, intense precipitation can wash out mosquito habitats, reducing the population. Future studies to examine the effect of rainfall on dengue outbreaks in Peru may warrant a more localized or flexible approach, including lags.

Finally, our modeling choice has attendant limitations. Effect estimates from ICAR models must be interpreted conditionally, that is for an “average” district month with a spatial random effect of 0. We chose splines to handle temporal autocorrelation and to adjust for longer-term temporal effects than those relevant to our research question. We made this decision due to the computational challenge of modeling nearly 160,000 temporal random effects (225 months x 701 study districts), and to facilitate interpretation of resulting effect estimates; use of random walk or autoregressive random effect on time would render our estimates conditional on both spatial and temporal random effects, but the simple interpretation of “an average month with a temporal random effect of 0” does not readily follow. Inspection of residual partial autocorrelation functions in a random sample of districts, included in S1 Fig, indicates no residual temporal autocorrelation. Lastly, while we are confident that the wide priors we have specified for our Bayesian hierarchical models combined with the richness of our dataset protect against undue influence of these priors on our findings, we did not conduct sensitivity analyses in this regard.

## Conclusions

Despite its limitations, our study utilizes a principled approach and a rich dataset to examine the effect of local weather and climactic factors on the risk of dengue outbreaks in Peru. Namely, we have demonstrated the potential use and importance of temperature and ENSO on dengue outbreak risk, suggesting these variables could be important predictors in outbreak prediction models, particularly in the summer and in the Selva Alta and Costa regions. Furthermore, the relationships characterized here may hold for other *Aedes*-vectored diseases. In light of the expected increase in frequency and severity of El Niño events in coming decades due to climate change [52, 53], and limited utility of currently-licensed vaccines [54, 55], strengthening capacity to predict and mitigate arbovirus outbreaks is all the more urgent.

## Supporting information

S1 Fig Partial autocorrelation functions for temperature and precipitation model and for all El Niño models for a random sample of 10 districts. Autocorrelation is indicated by taller bars at shorter lags (i.e., decreasing bar heights from left to right)].
(PDF)
