# Supplementary material for: The effect of weather and climate on dengue outbreak risk in Peru, 2000-2018: A time-series analysis
Source: PLoS Negl Trop Dis. 2022 Jun 30;16(6):e0010479. doi: 10.1371/journal.pntd.0010479 (PMC9278784; doi:10.1371/journal.pntd.0010479)
Supplement: S1 Fig — Autocorrelation is indicated by taller bars at shorter lags (i.e., decreasing bar heights from left to right)]. (PDF) [file pntd.0010479.s001.pdf]

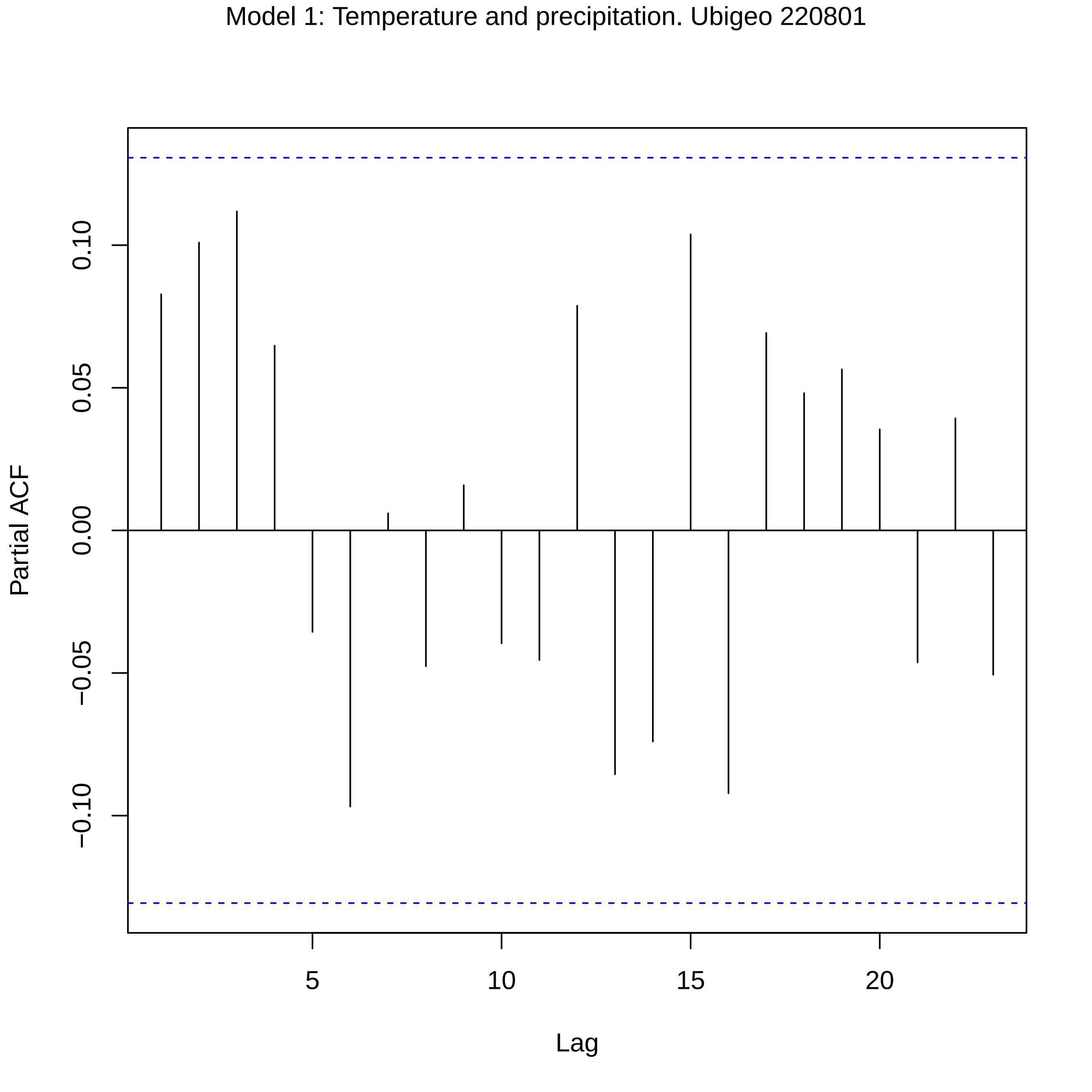

Model 1: Temperature and precipitation. Ubigeo 220801
Partial ACF
0.10
0.05
0.00
−0.05
−0.10
5
10
15
20
Lag

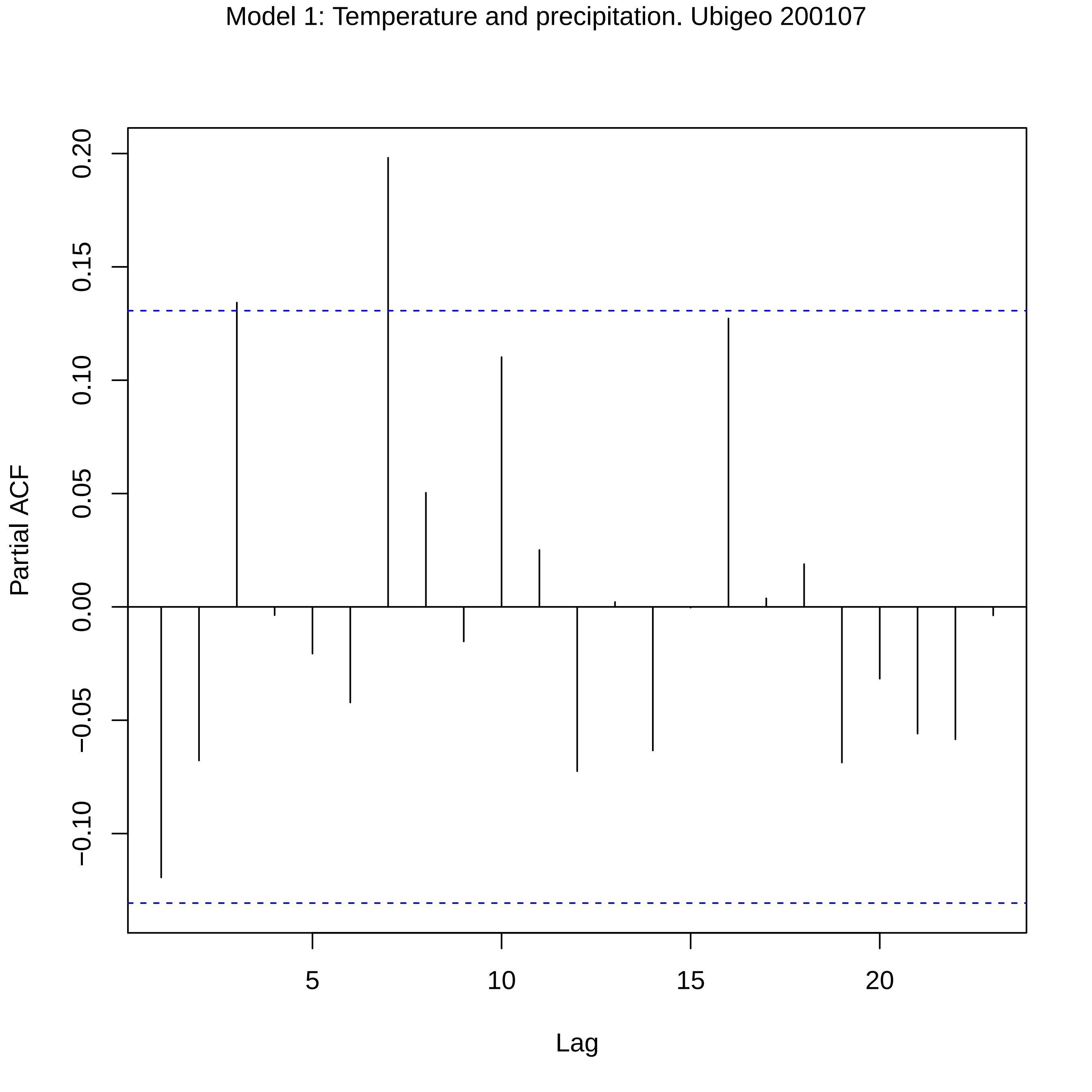
Model 1: Temperature and precipitation. Ubigeo 200107
Partial ACF
0.20
0.15
0.10
0.05
0.00
−0.05
−0.10
5
10
15
20
Lag

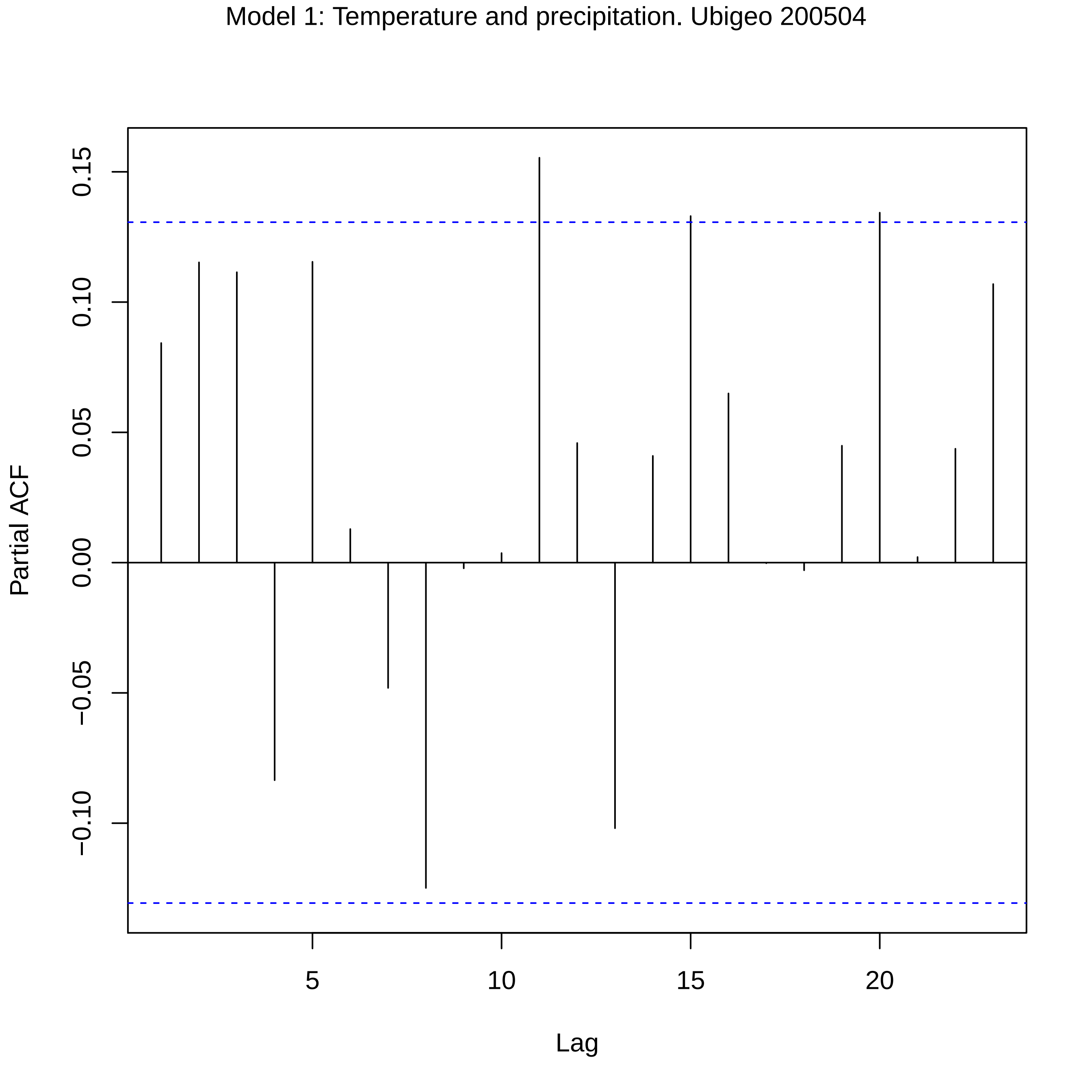

Model 1: Temperature and precipitation. Ubigeo 200504
Partial ACF
0.15
0.10
0.05
0.00
−0.05
−0.10
5
10
15
20
Lag

Model 1: Temperature and precipitation. Ubigeo 220603

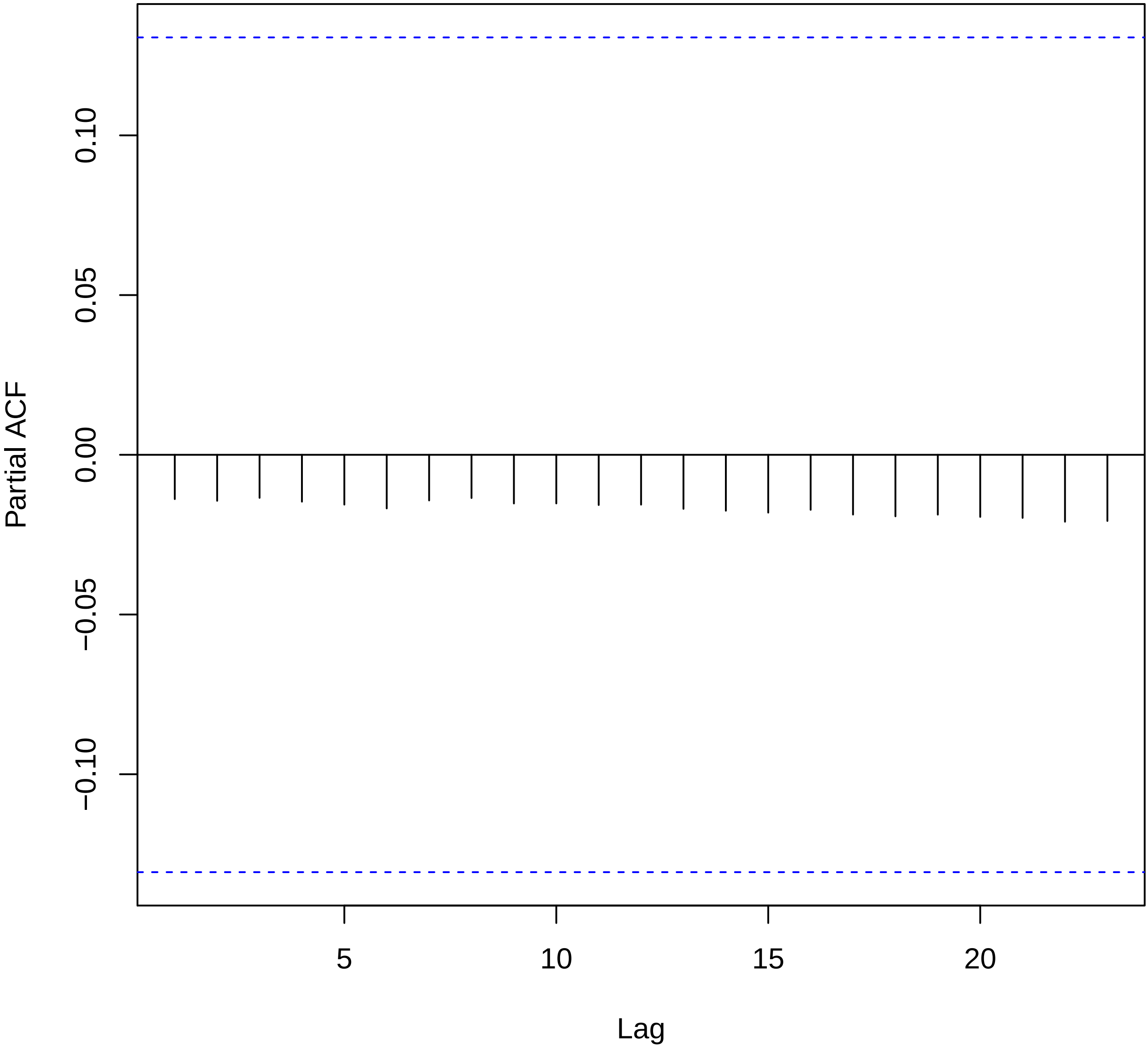

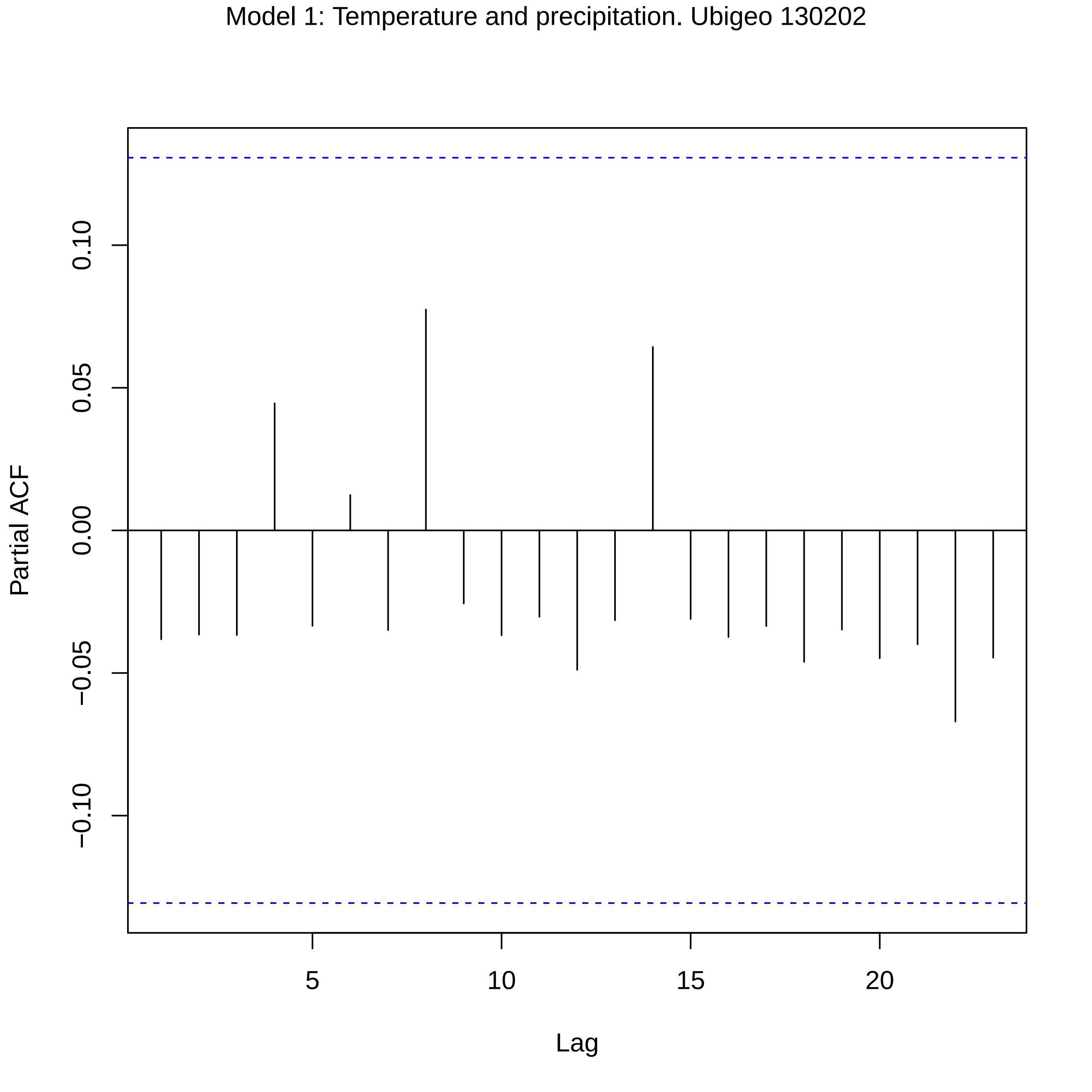

Model 1: Temperature and precipitation. Ubigeo 130202
Partial ACF
0.10
0.05
0.00
−0.05
−0.10
5
10
15
20
Lag

Model 1: Temperature and precipitation. Ubigeo 110105

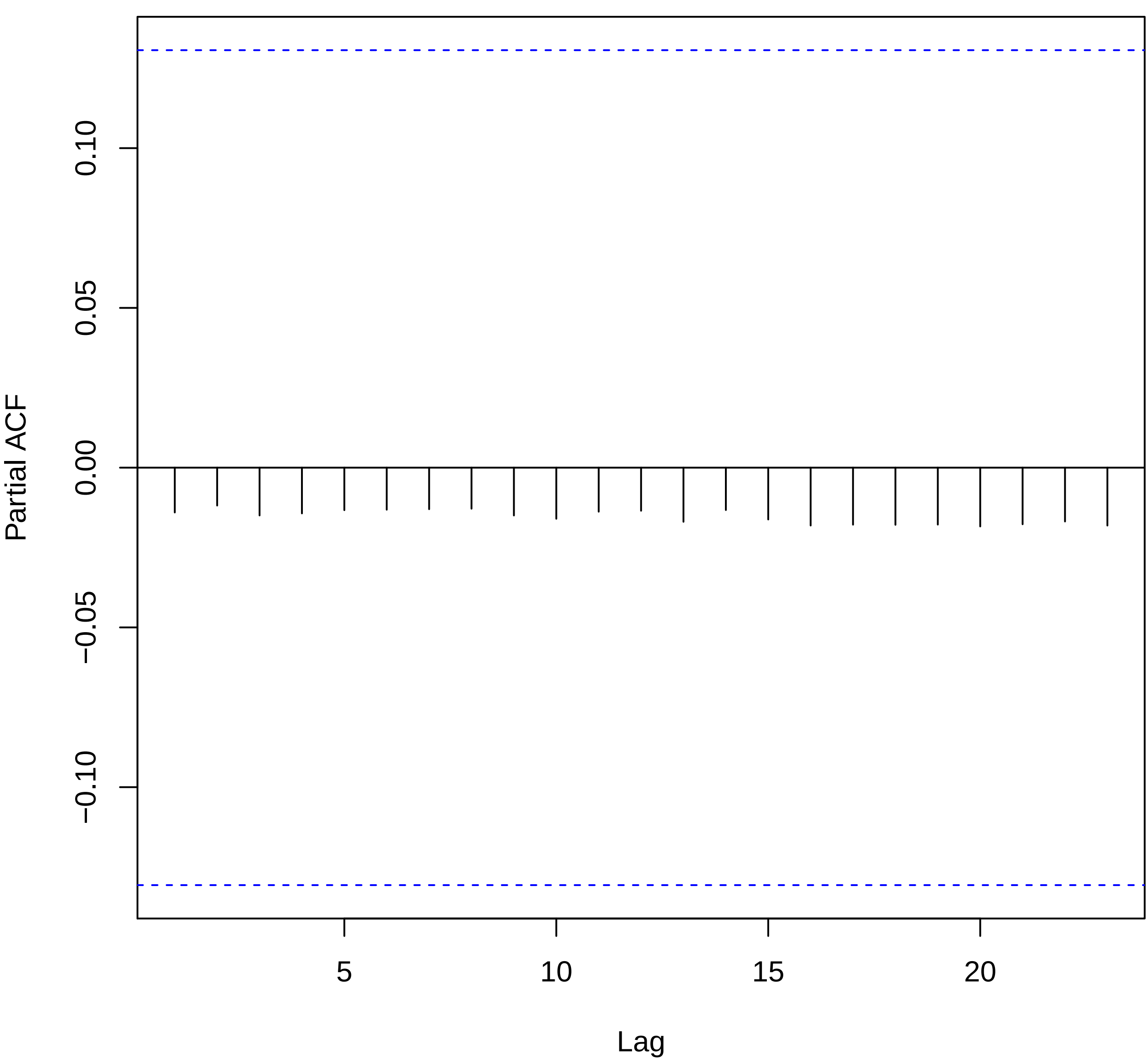

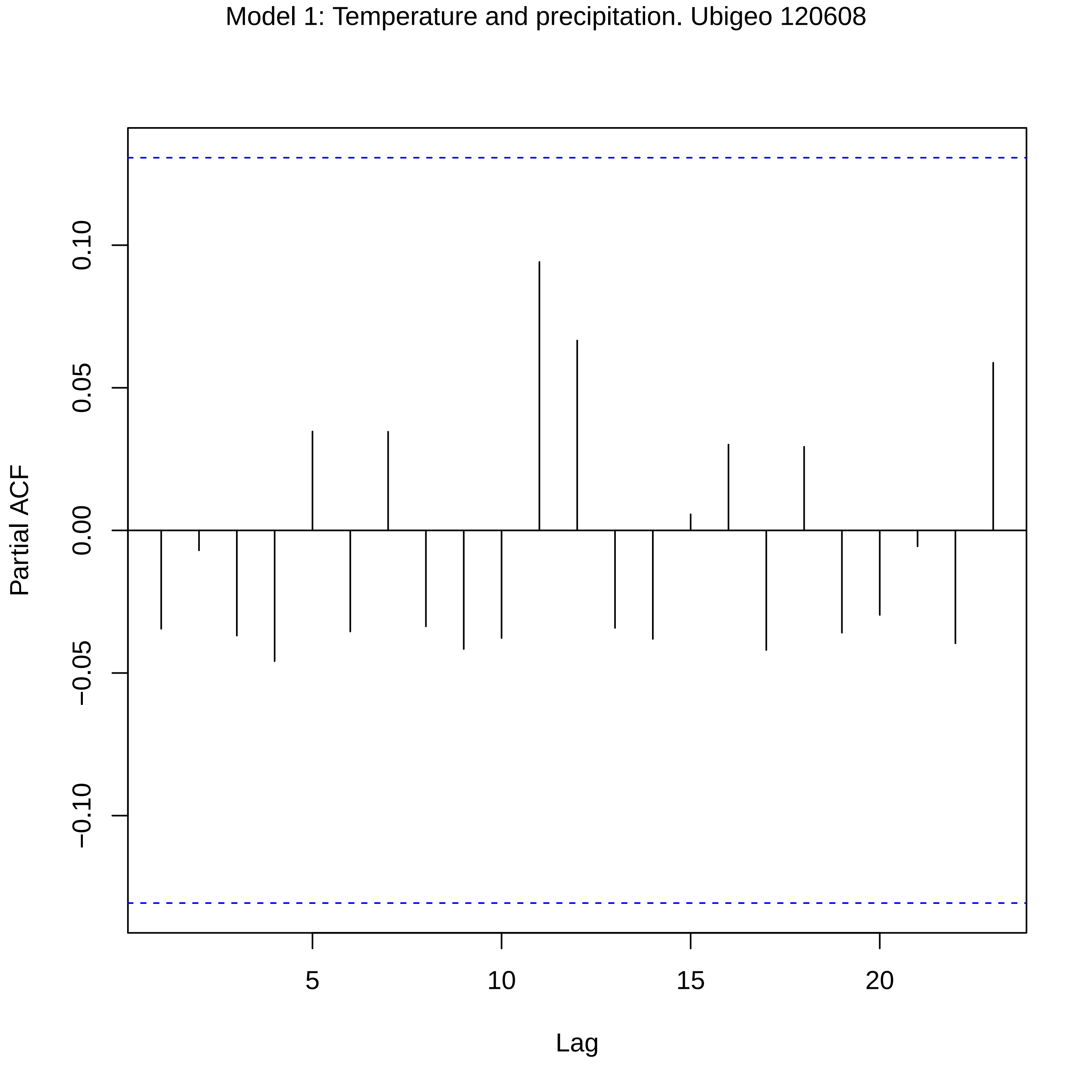
Model 1: Temperature and precipitation. Ubigeo 120608
Partial ACF
0.10
0.05
0.00
−0.05
−0.10
5
10
15
20
Lag

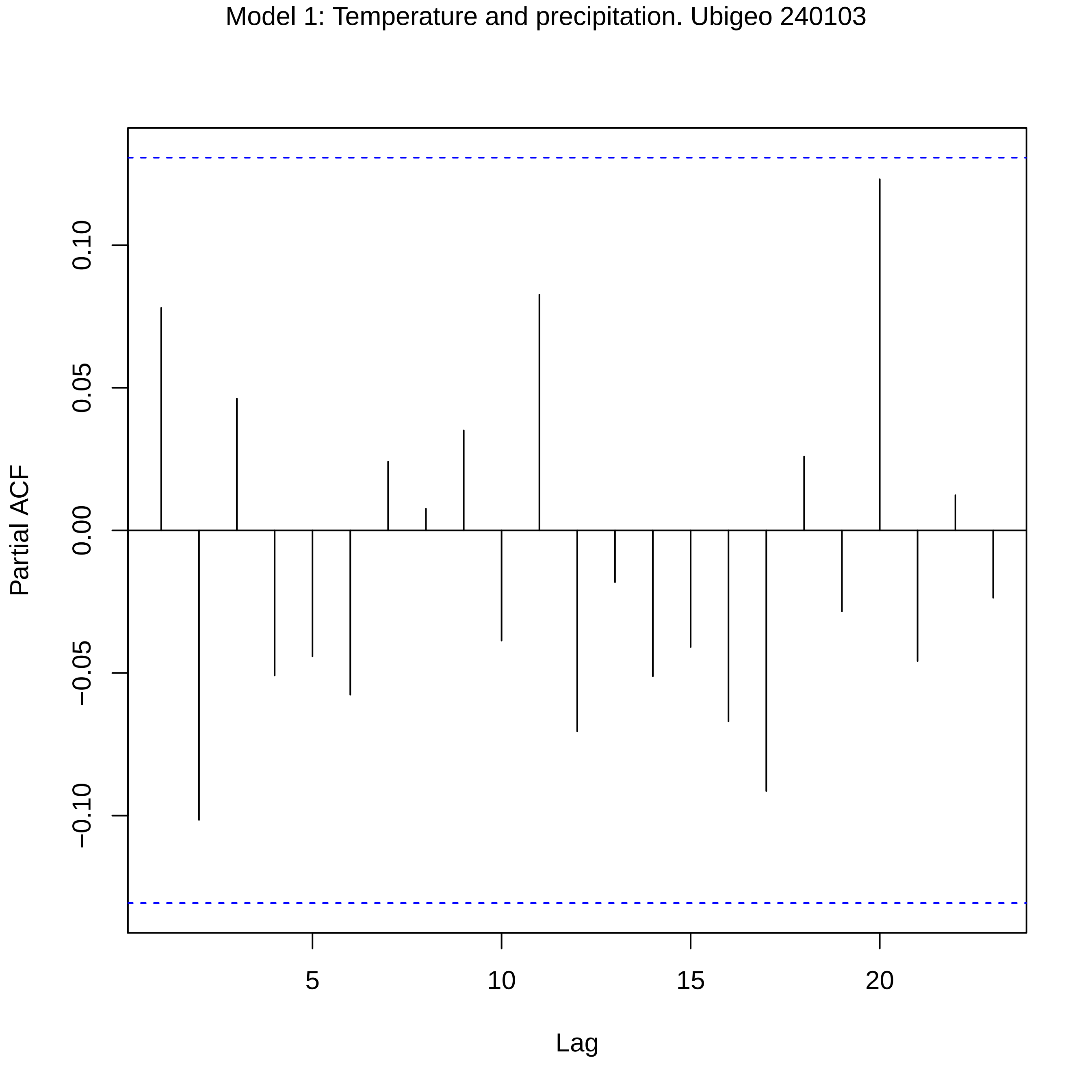

Model 1: Temperature and precipitation. Ubigeo 240103
Partial ACF
0.10
0.05
0.00
−0.05
−0.10
5
10
15
20
Lag

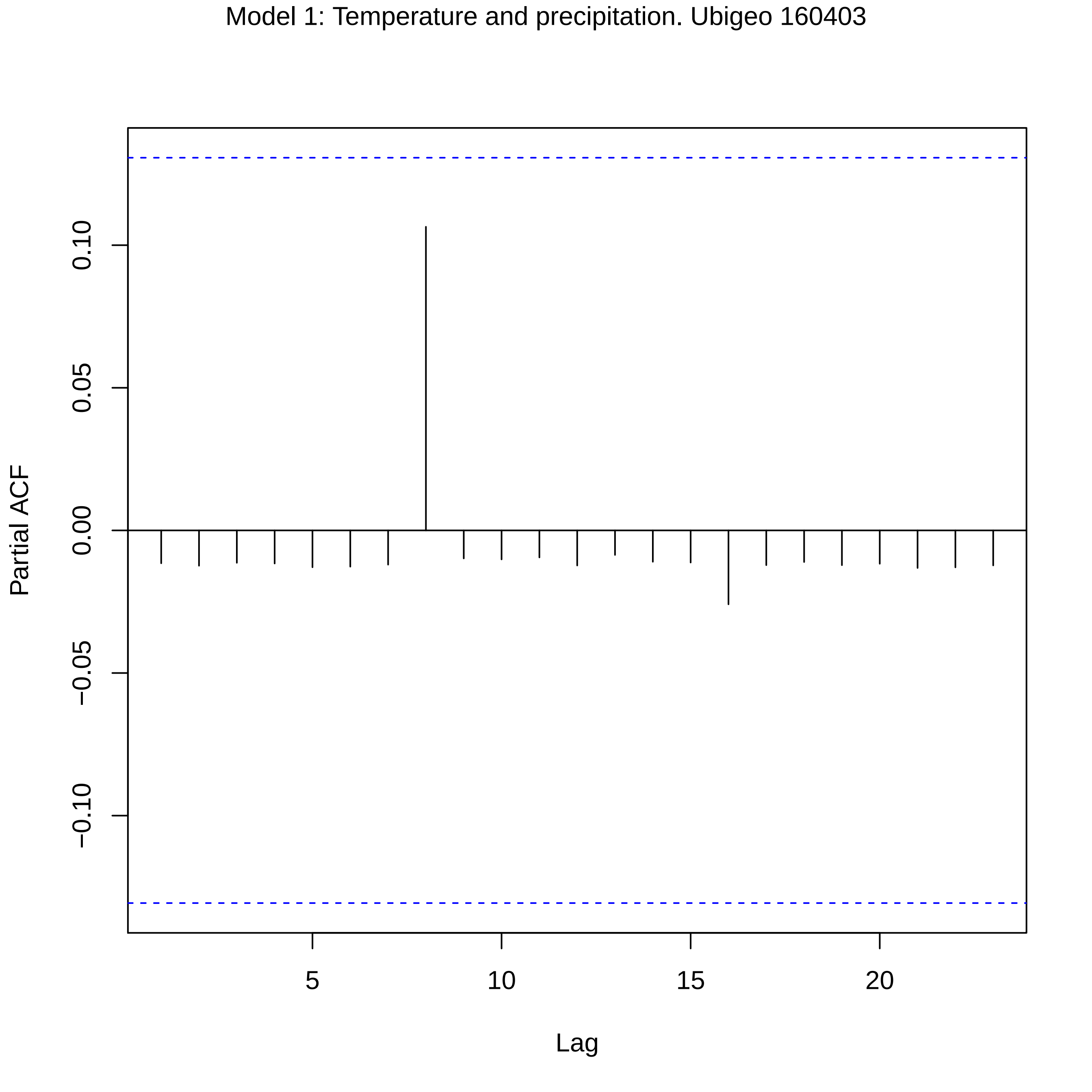
Model 1: Temperature and precipitation. Ubigeo 160403
Partial ACF
0.10
0.05
0.00
−0.05
−0.10
5
10
15
20
Lag

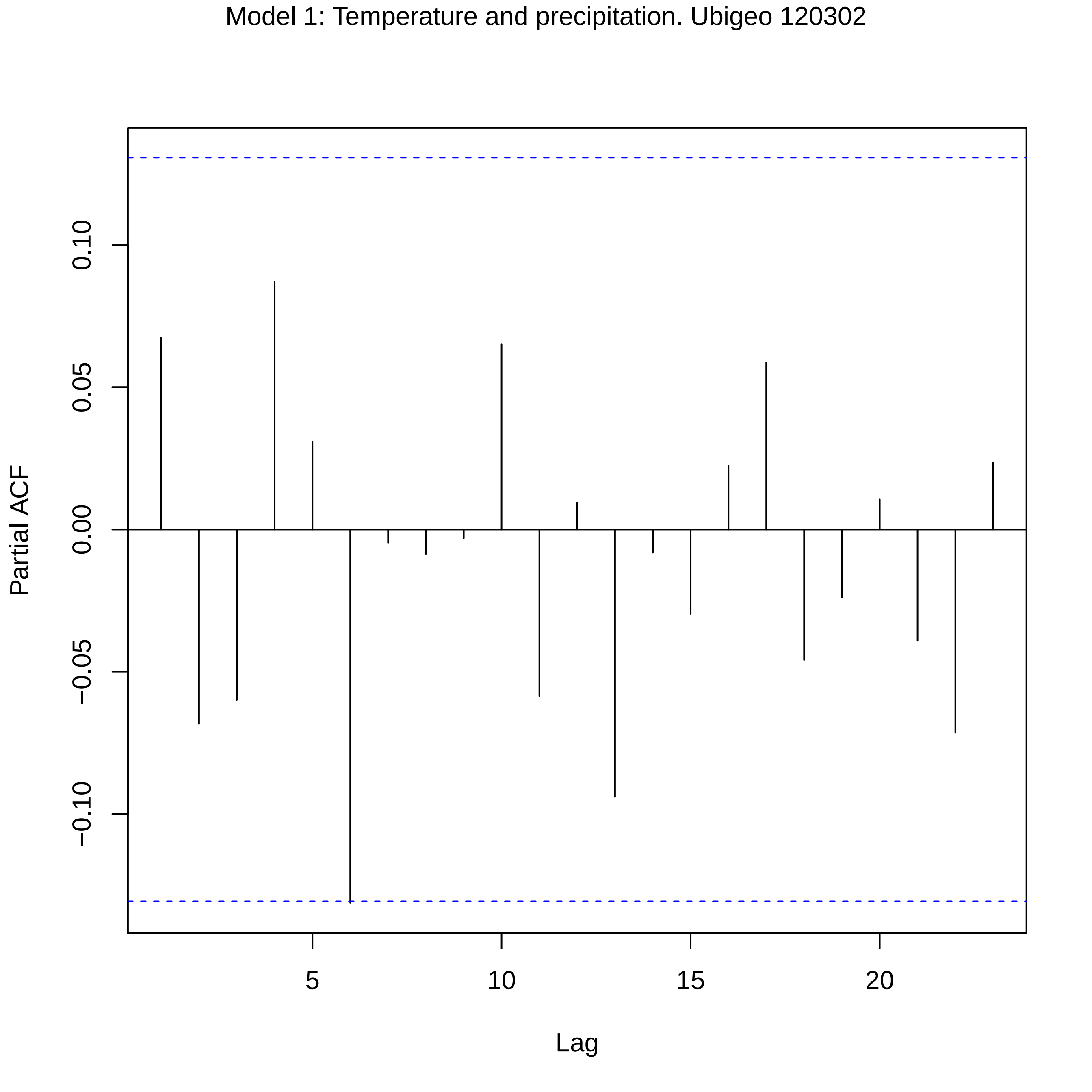

Model 1: Temperature and precipitation. Ubigeo 120302
Partial ACF
0.10
0.05
0.00
−0.05
−0.10
5
10
15
20
Lag

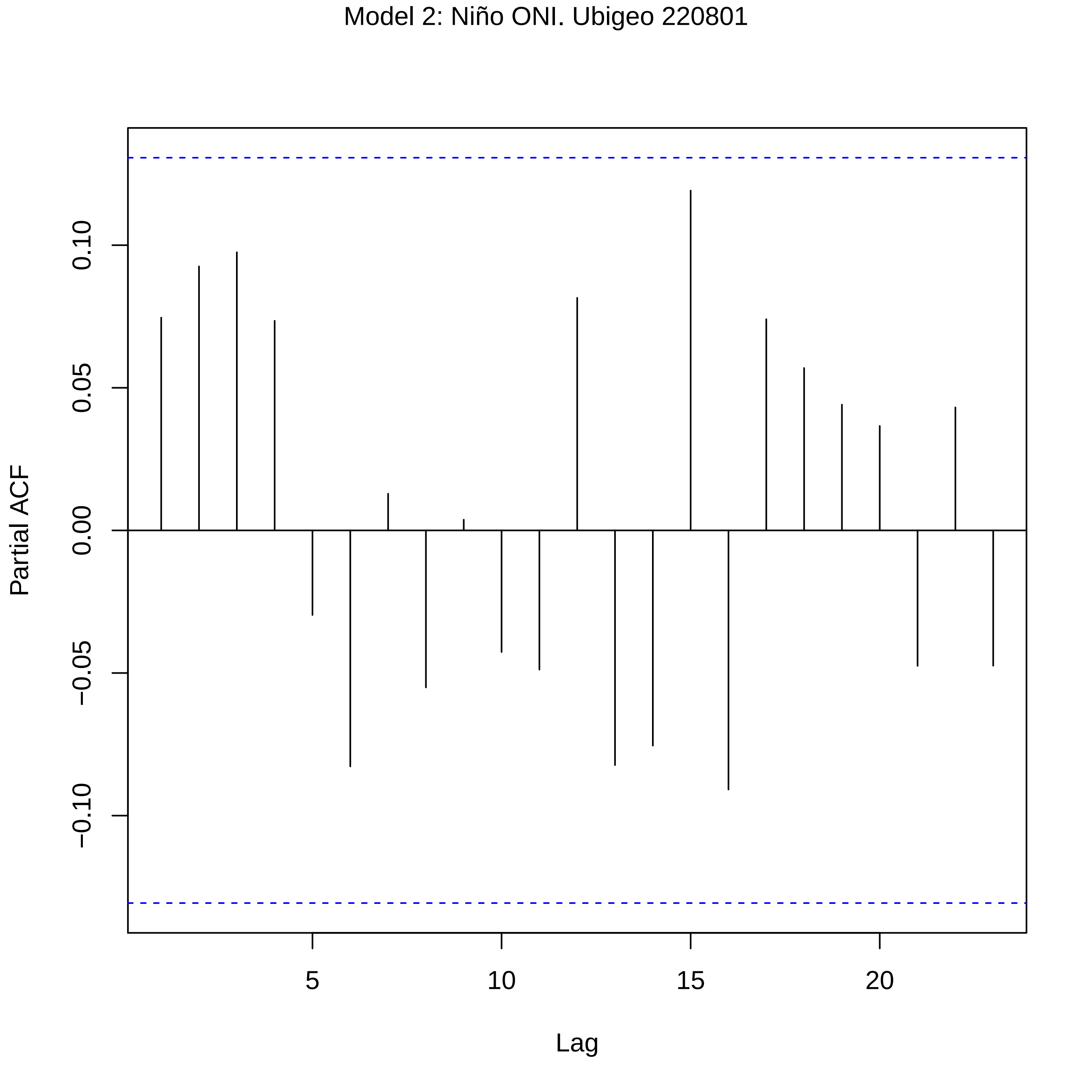

Model 2: Niño ONI. Ubigeo 220801
Partial ACF
0.10
0.05
0.00
−0.05
−0.10
5
10
15
20
Lag

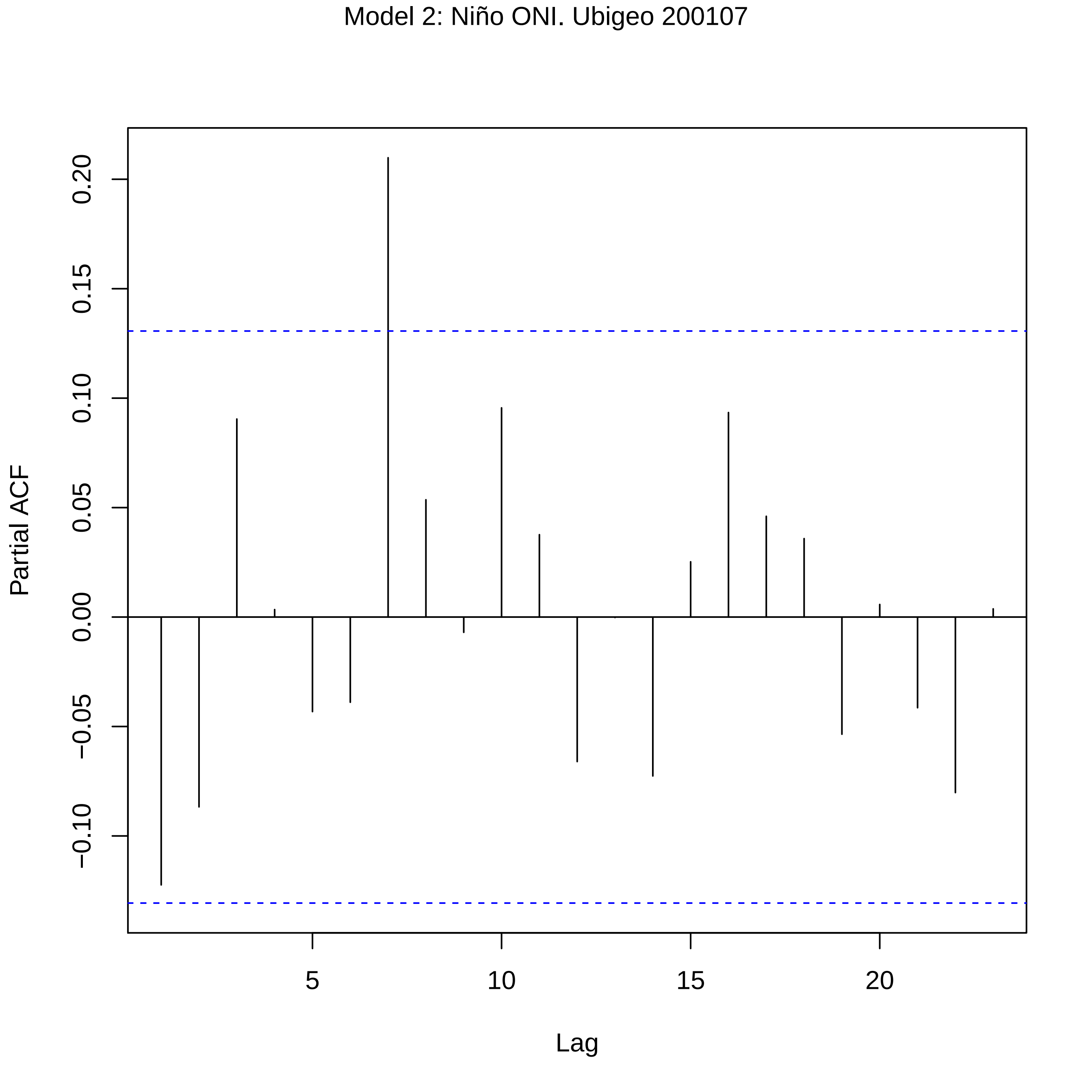
Model 2: Niño ONI. Ubigeo 200107
Partial ACF
Lag
0.20
0.15
0.10
0.05
0.00
−0.05
−0.10
5
10
15
20

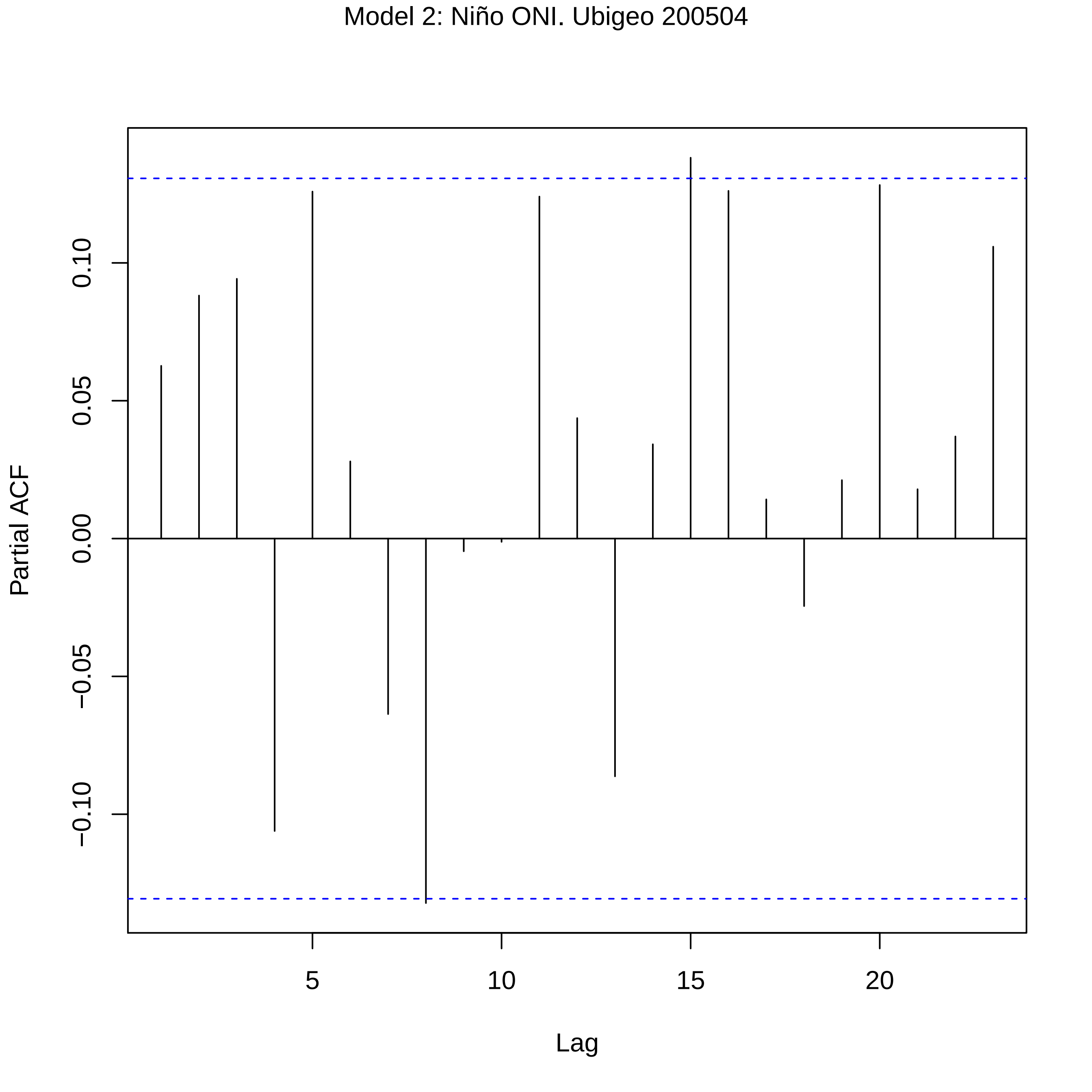

Model 2: Niño ONI. Ubigeo 200504
Partial ACF
0.10
0.05
0.00
−0.05
−0.10
5
10
15
20
Lag

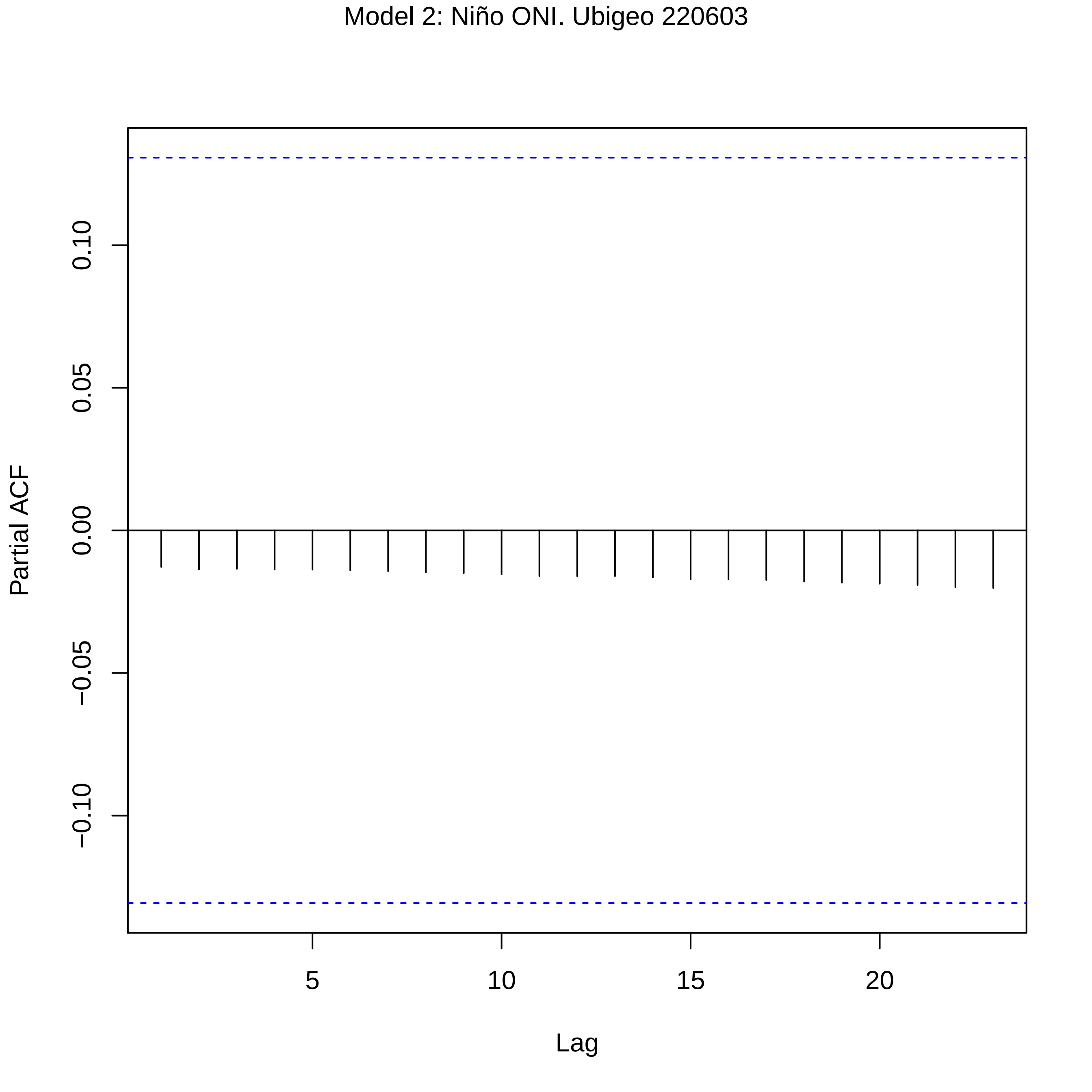
Model 2: Niño ONI. Ubigeo 220603
Partial ACF
0.10
0.05
0.00
−0.05
−0.10
5
10
15
20
Lag

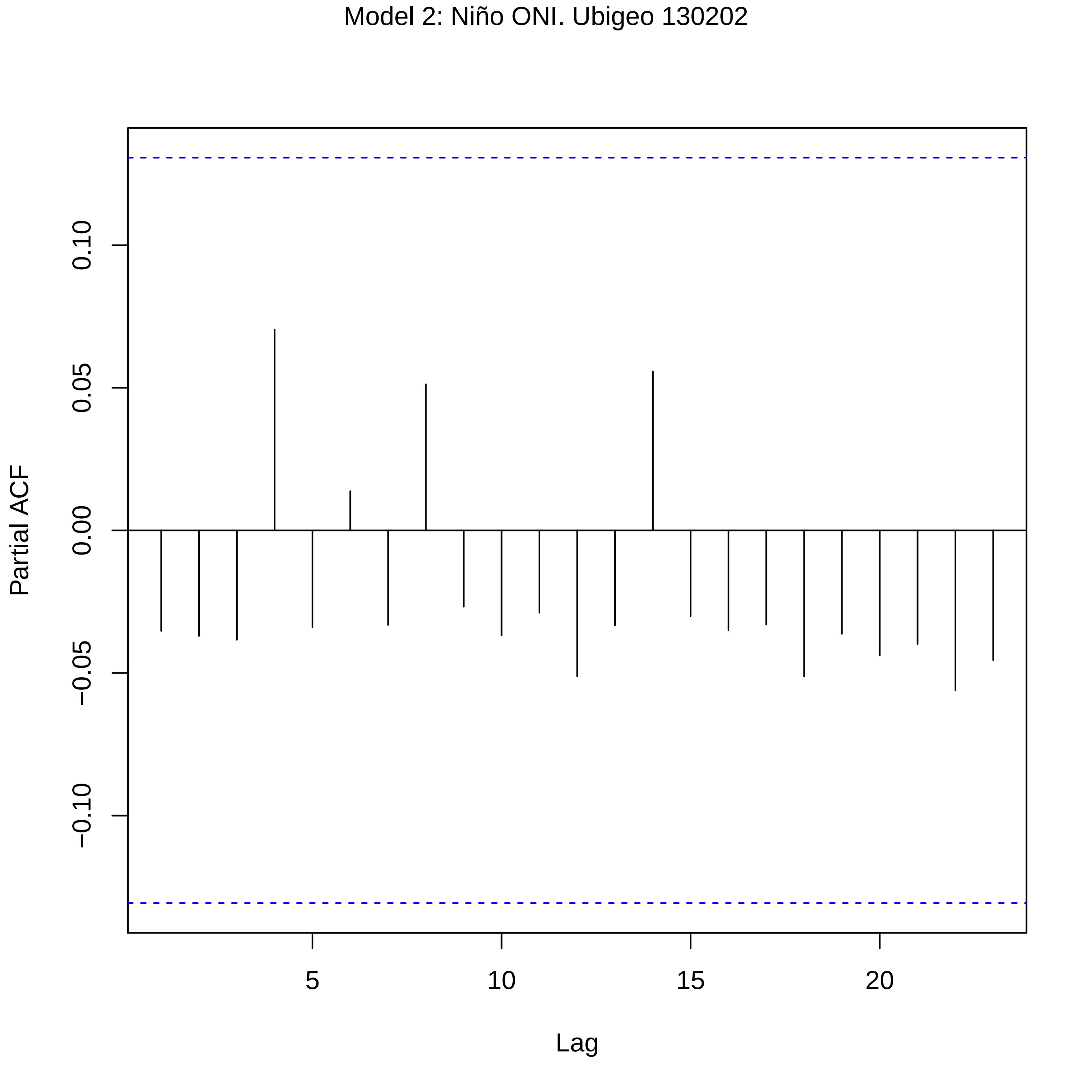
Model 2: Niño ONI. Ubigeo 130202
Partial ACF
0.10
0.05
0.00
−0.05
−0.10
5
10
15
20
Lag

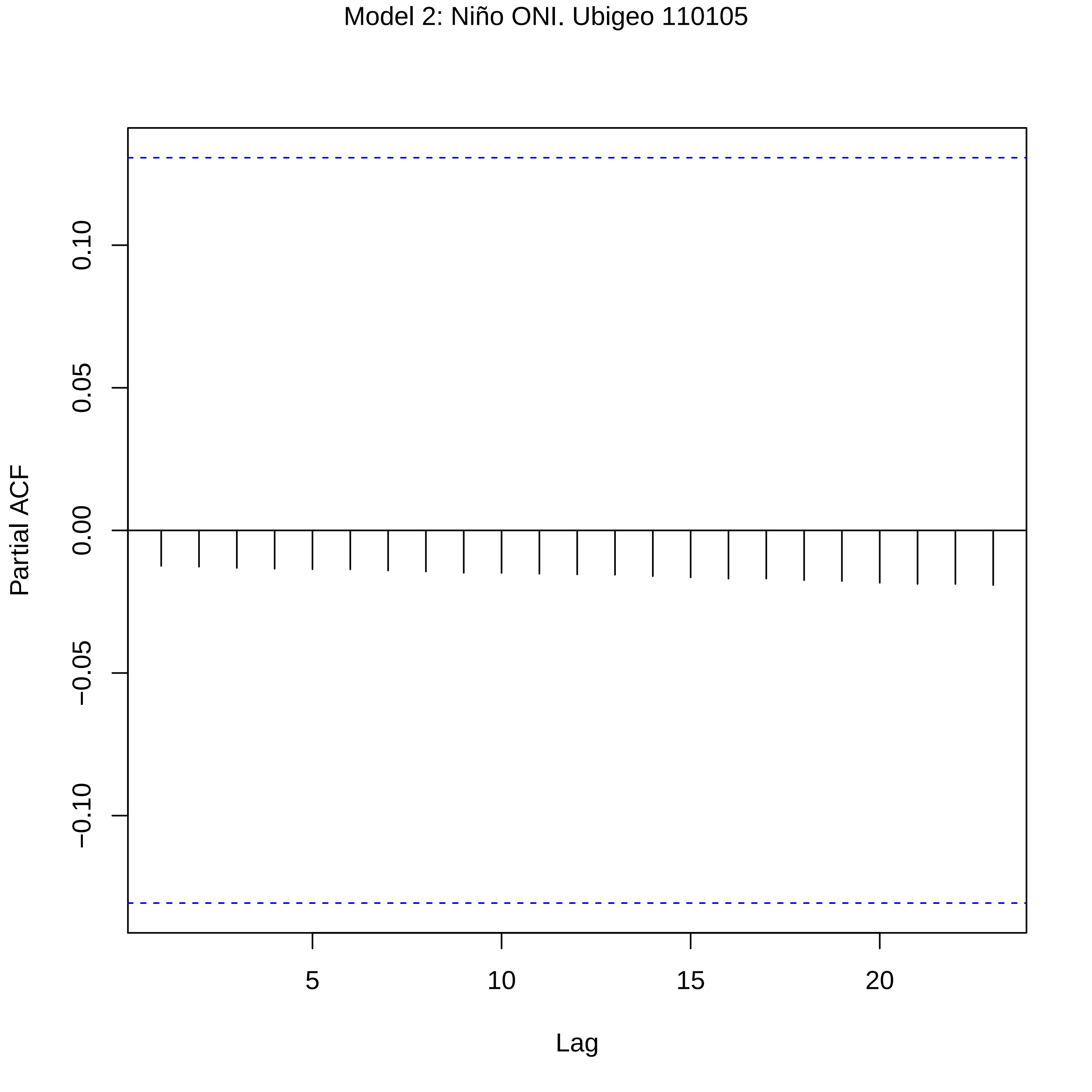

Model 2: Niño ONI. Ubigeo 110105
Partial ACF
0.10
0.05
0.00
−0.05
−0.10
5
10
15
20
Lag

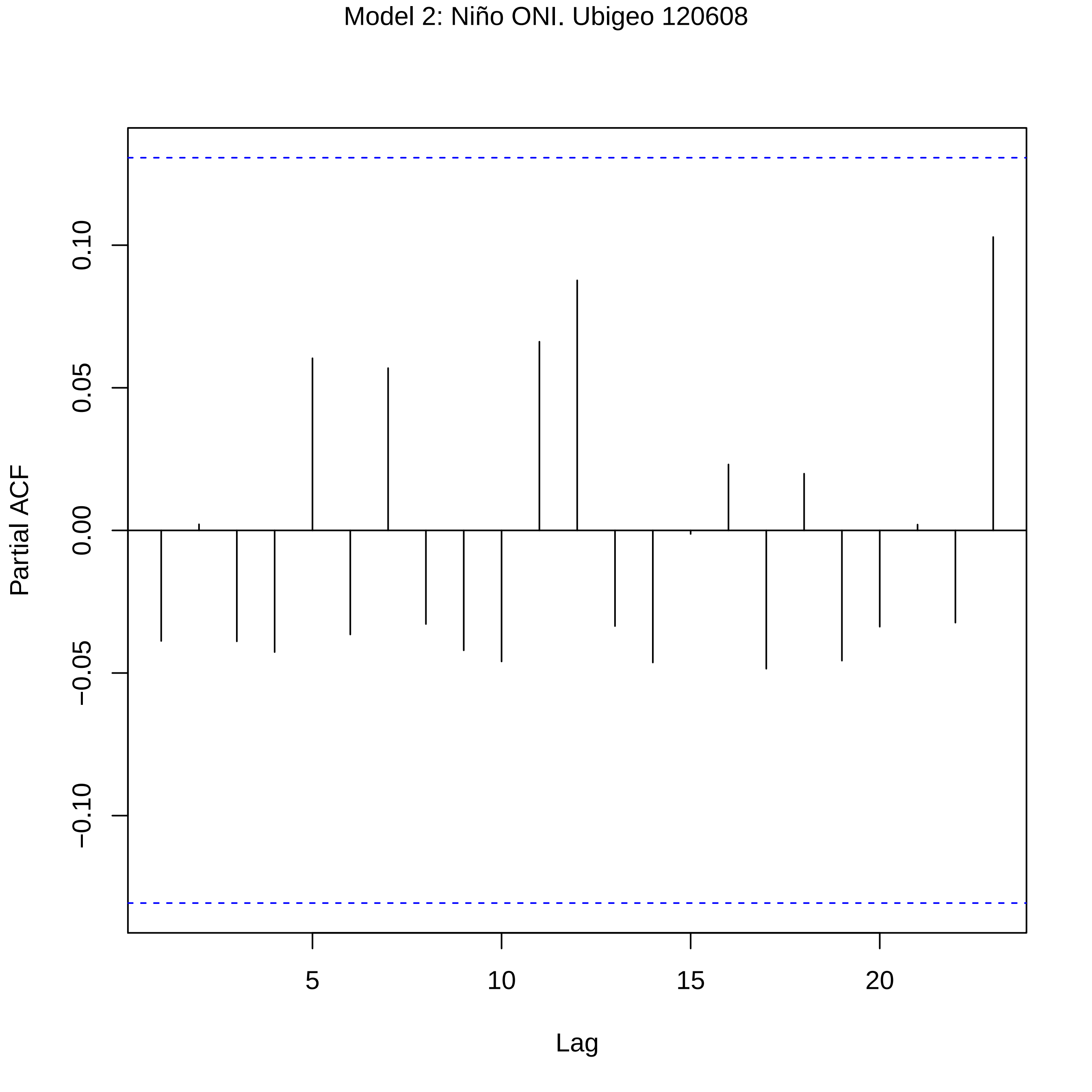

Model 2: Niño ONI. Ubigeo 120608
Partial ACF
0.10
0.05
0.00
−0.05
−0.10
5
10
15
20
Lag

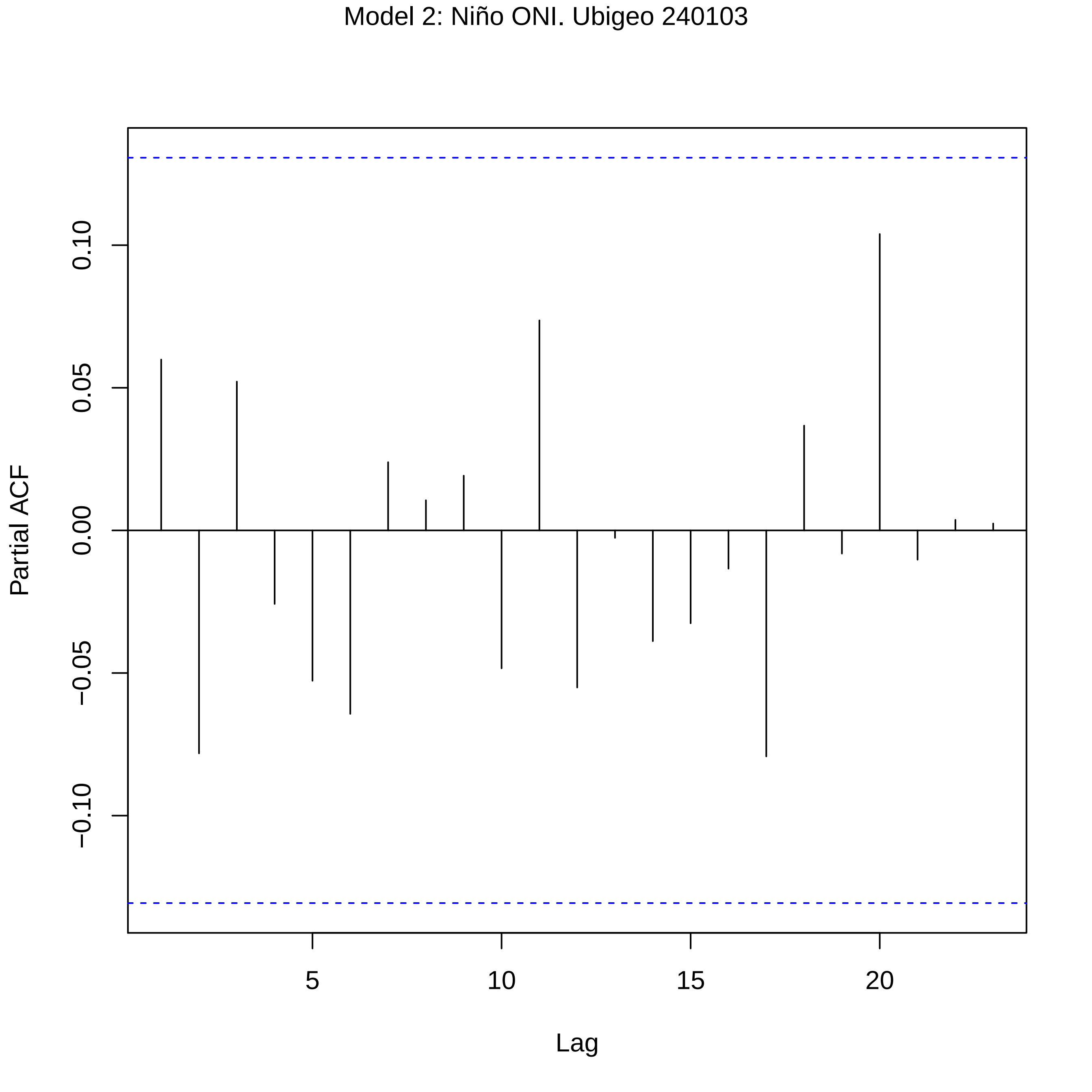
Model 2: Niño ONI. Ubigeo 240103
Partial ACF
0.10
0.05
0.00
−0.05
−0.10
5
10
15
20
Lag

Model 2: Niño ONI. Ubigeo 160403

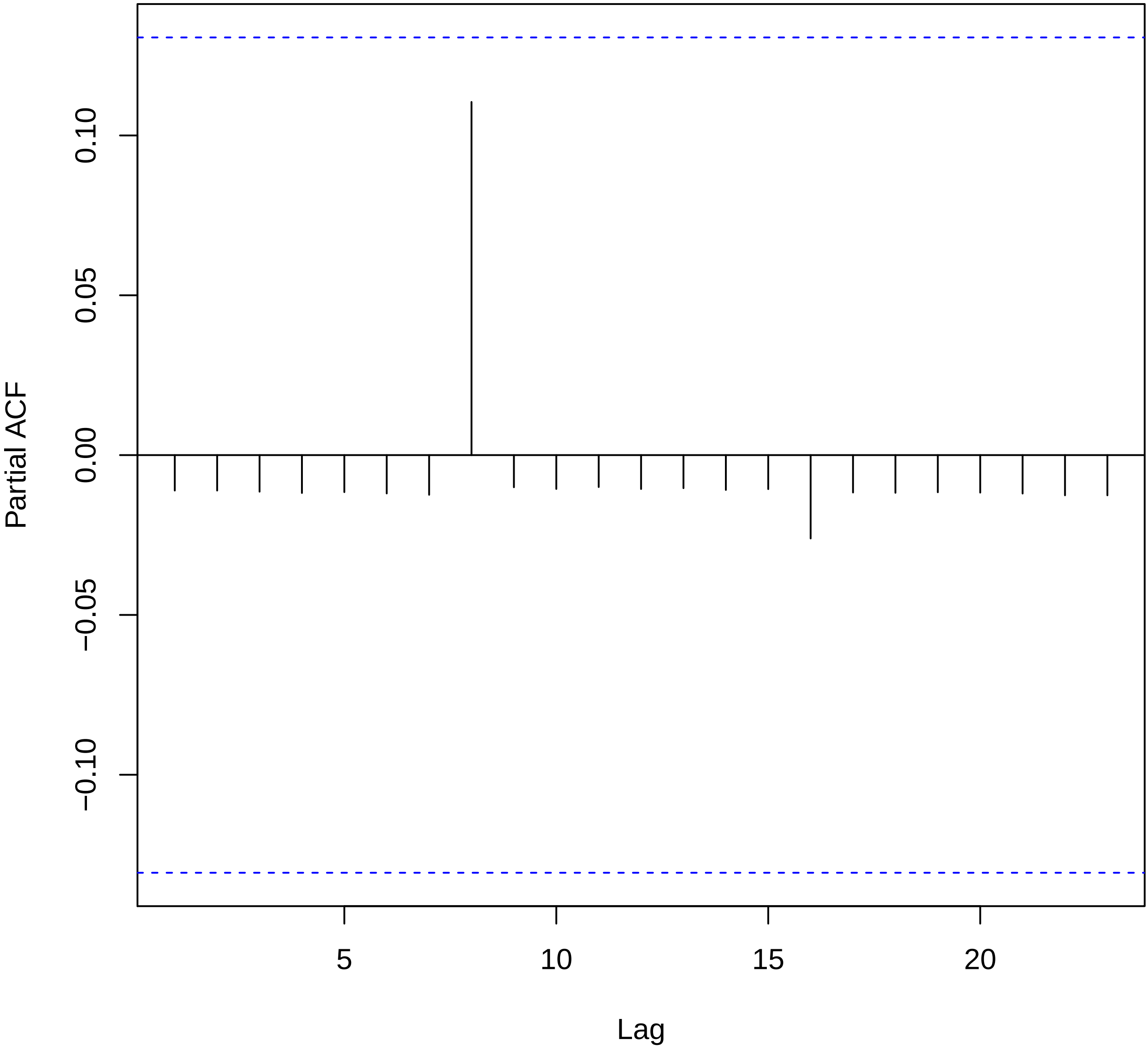

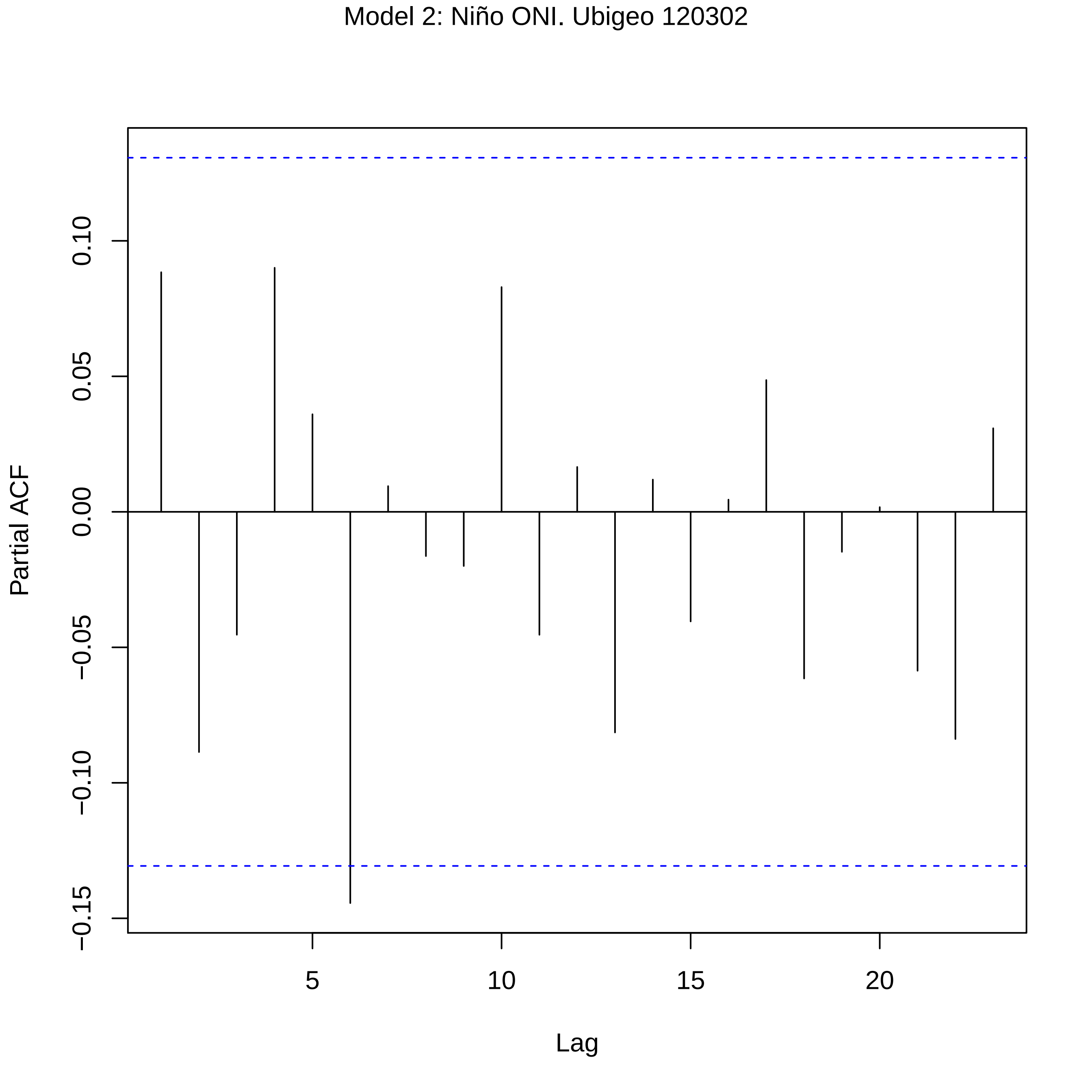

Model 2: Niño ONI. Ubigeo 120302
Partial ACF
0.10
0.05
0.00
−0.05
−0.10
−0.15
5
10
15
20
Lag

Model 3: Niño ICEN. Ubigeo 220801

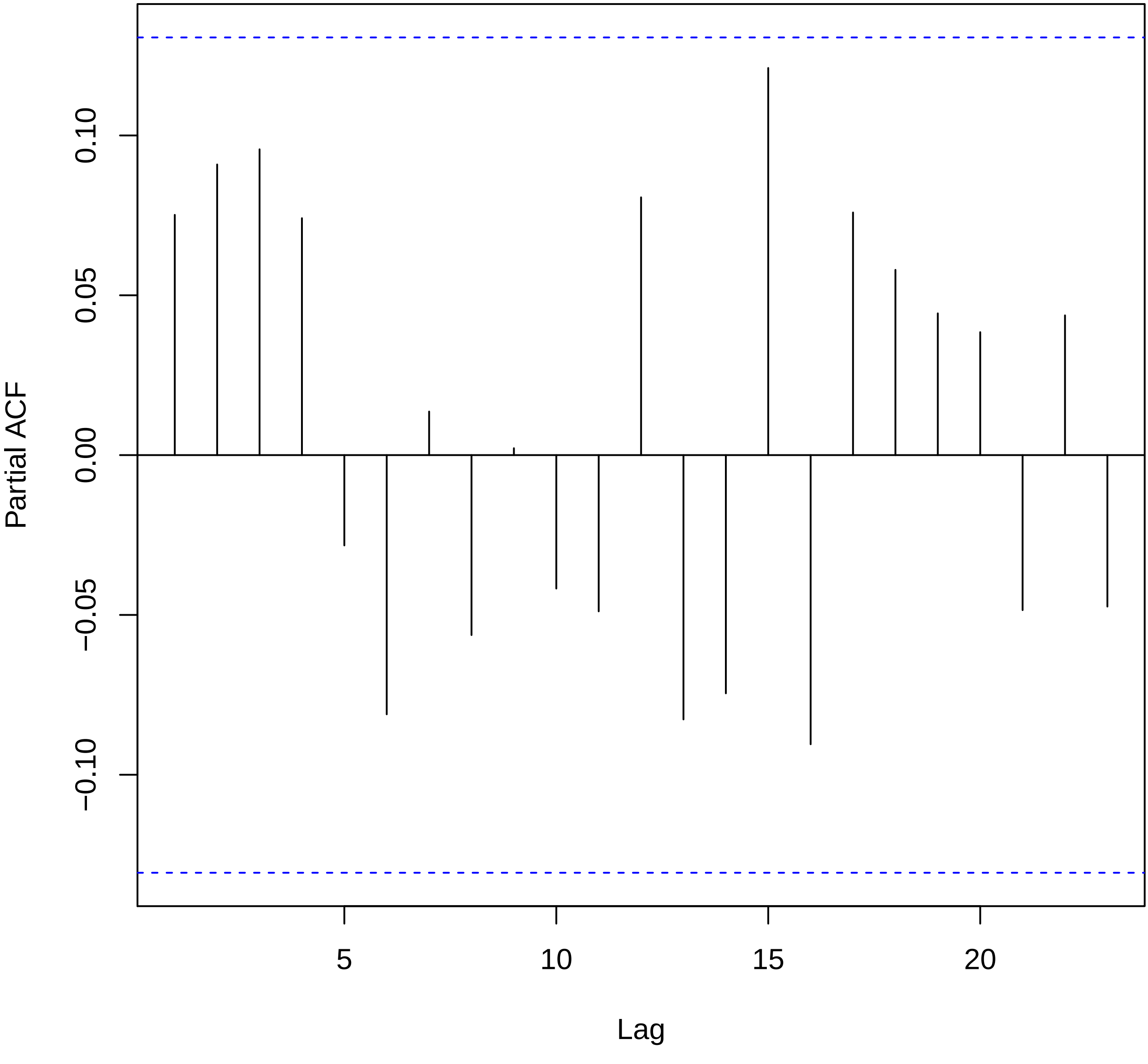

Model 3: Niño ICEN. Ubigeo 200107

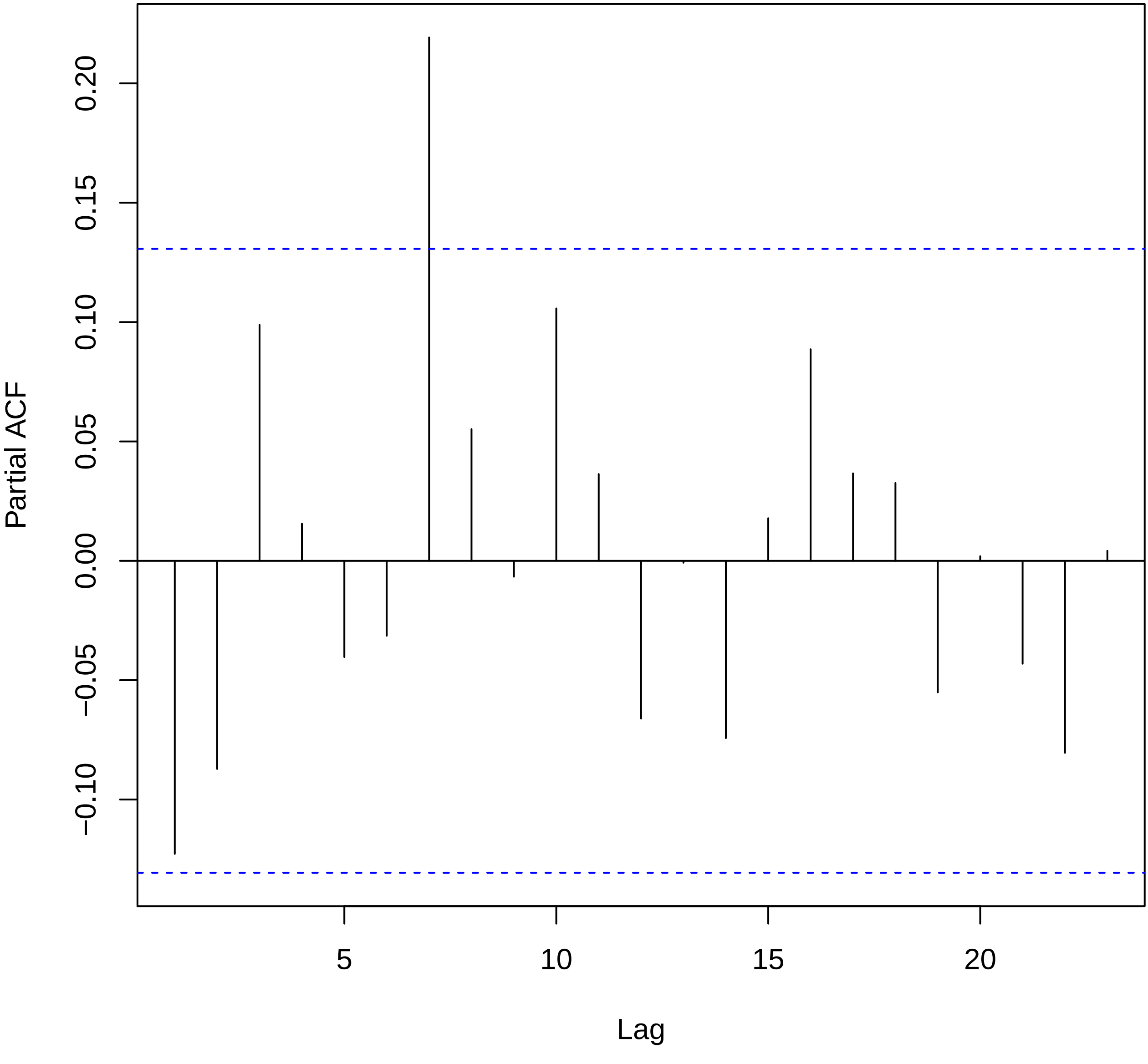

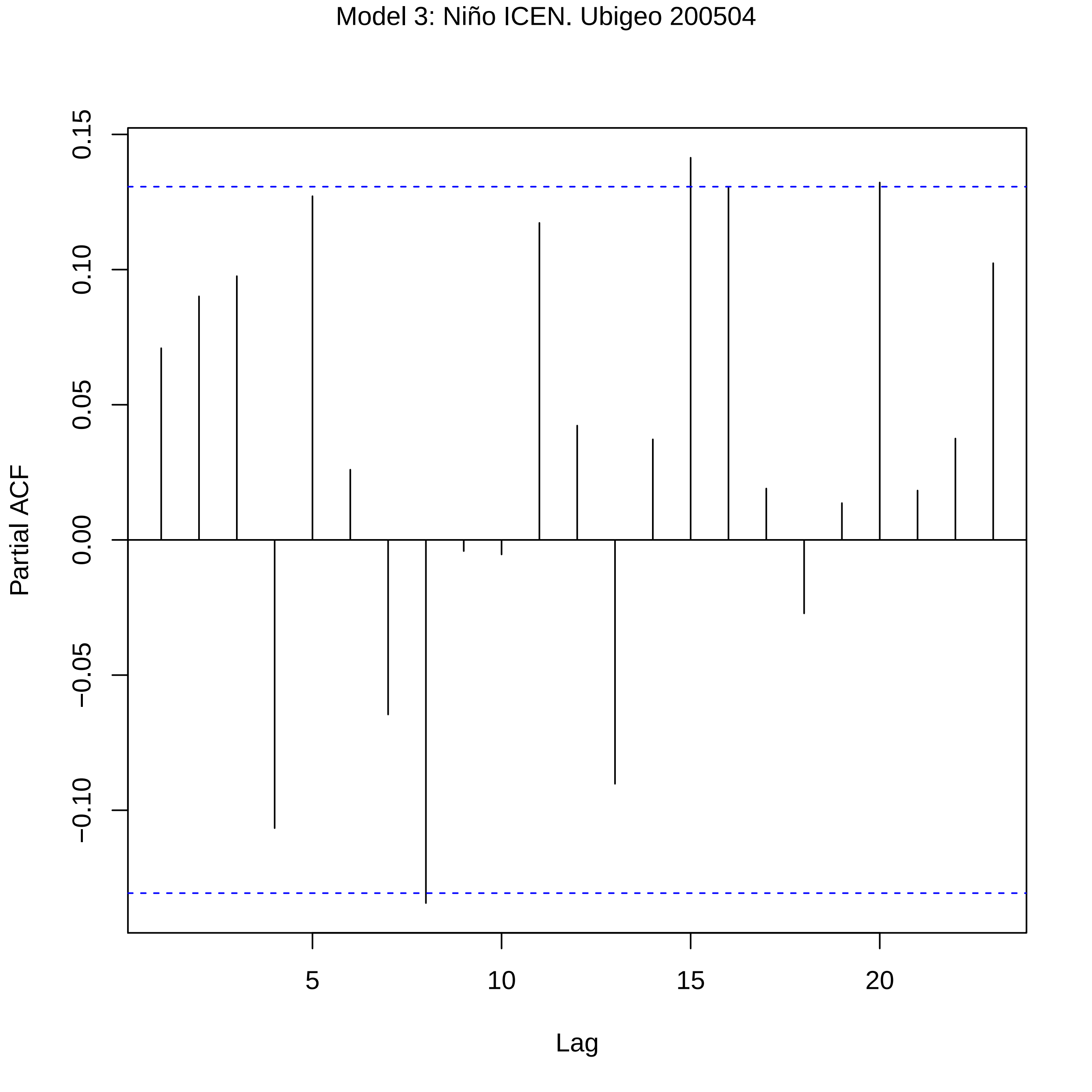
Model 3: Niño ICEN. Ubigeo 200504
Partial ACF
0.15
0.10
0.05
0.00
−0.05
−0.10
5
10
15
20
Lag

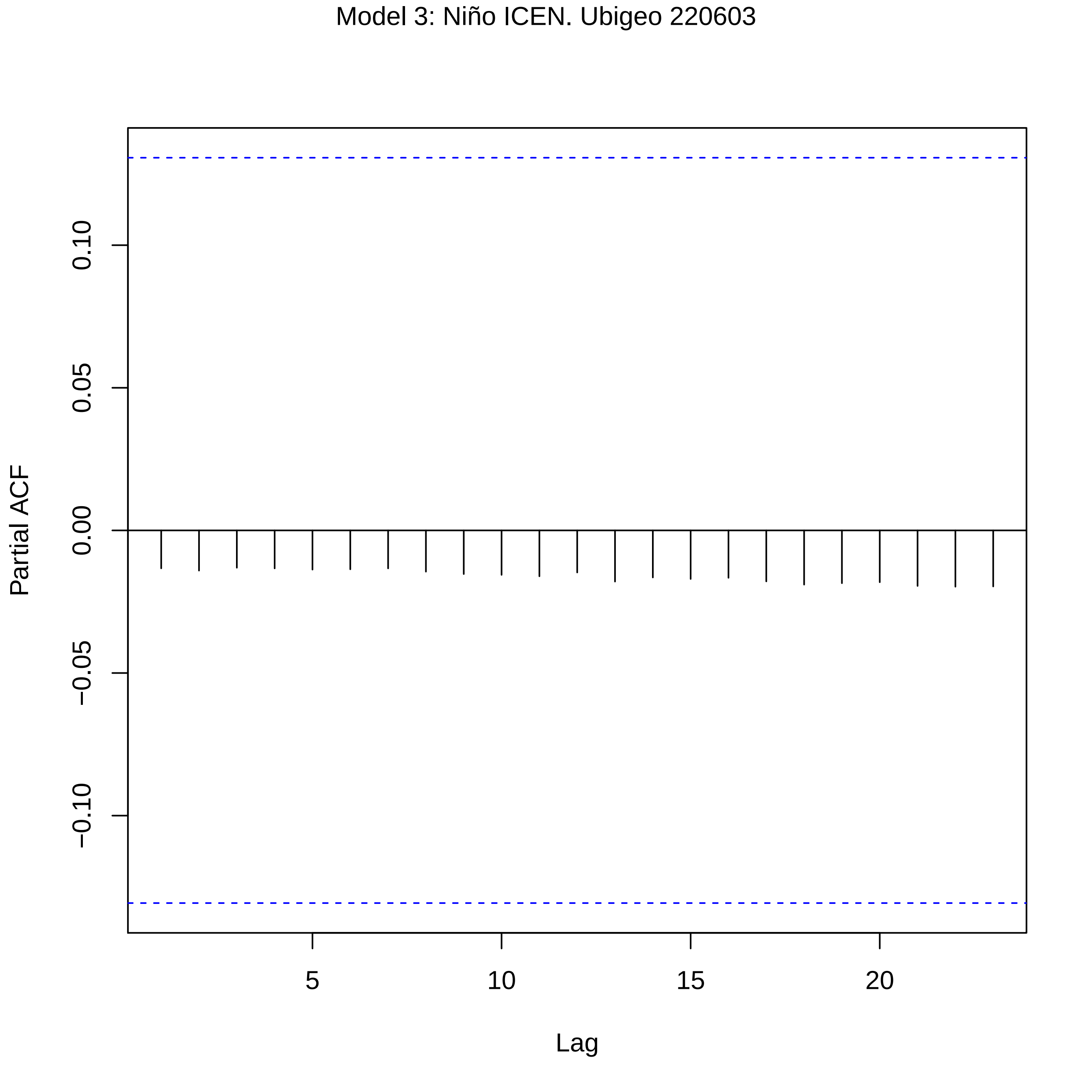

Model 3: Niño ICEN. Ubigeo 220603
Partial ACF
0.10
0.05
0.00
−0.05
−0.10
5
10
15
20
Lag

Model 3: Niño ICEN. Ubigeo 130202

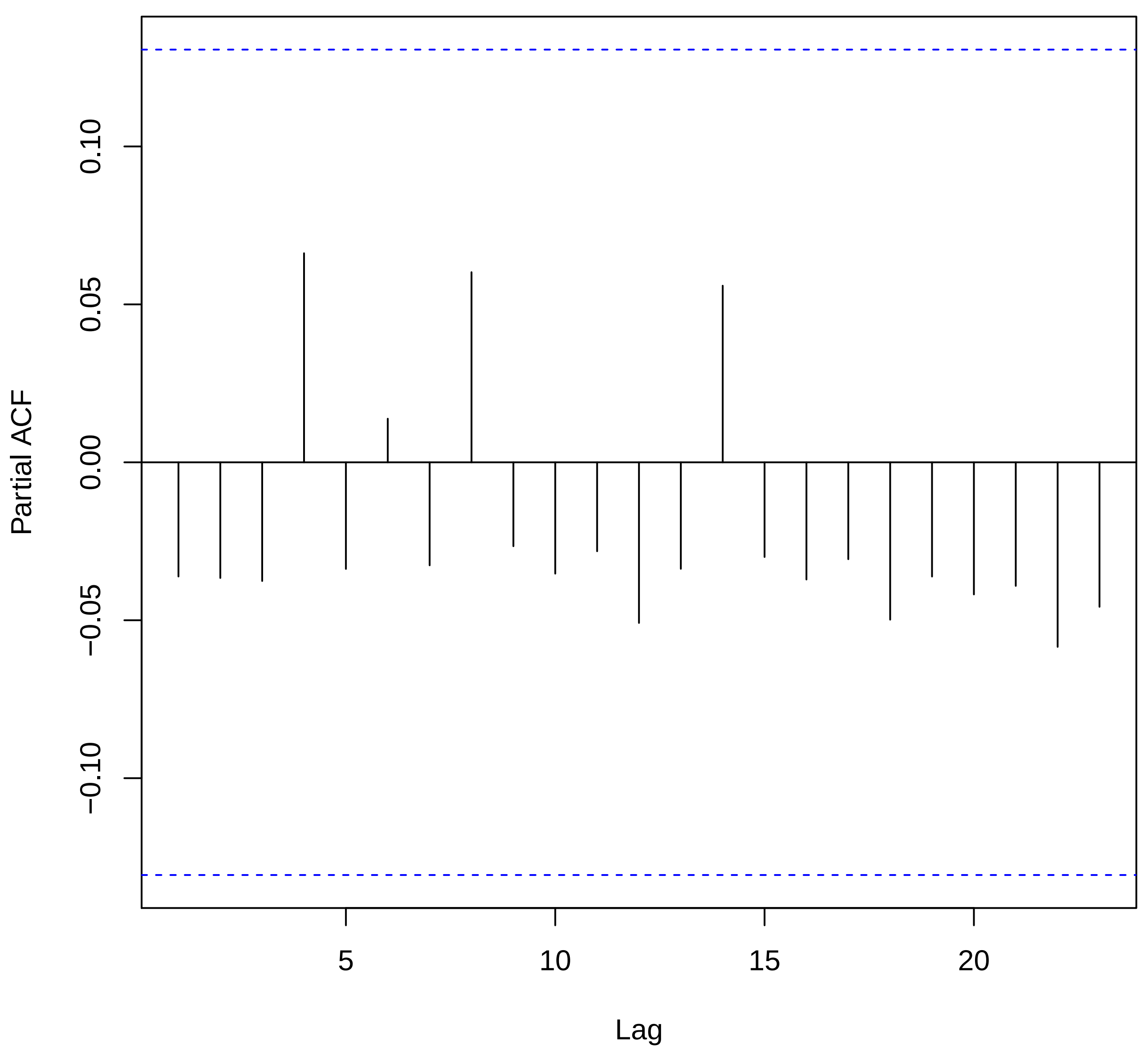

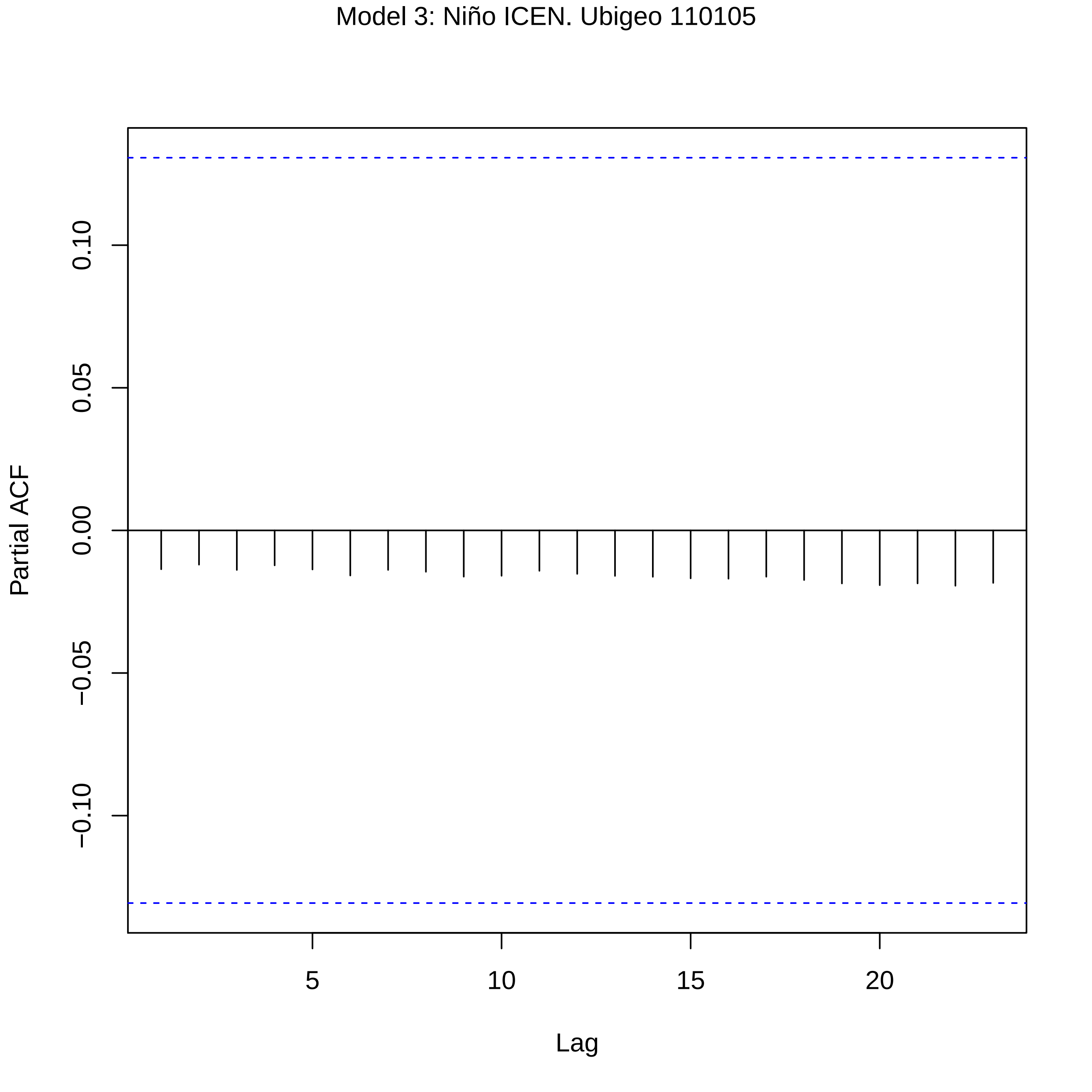

Model 3: Niño ICEN. Ubigeo 110105
Partial ACF
0.10
0.05
0.00
−0.05
−0.10
5
10
15
20
Lag

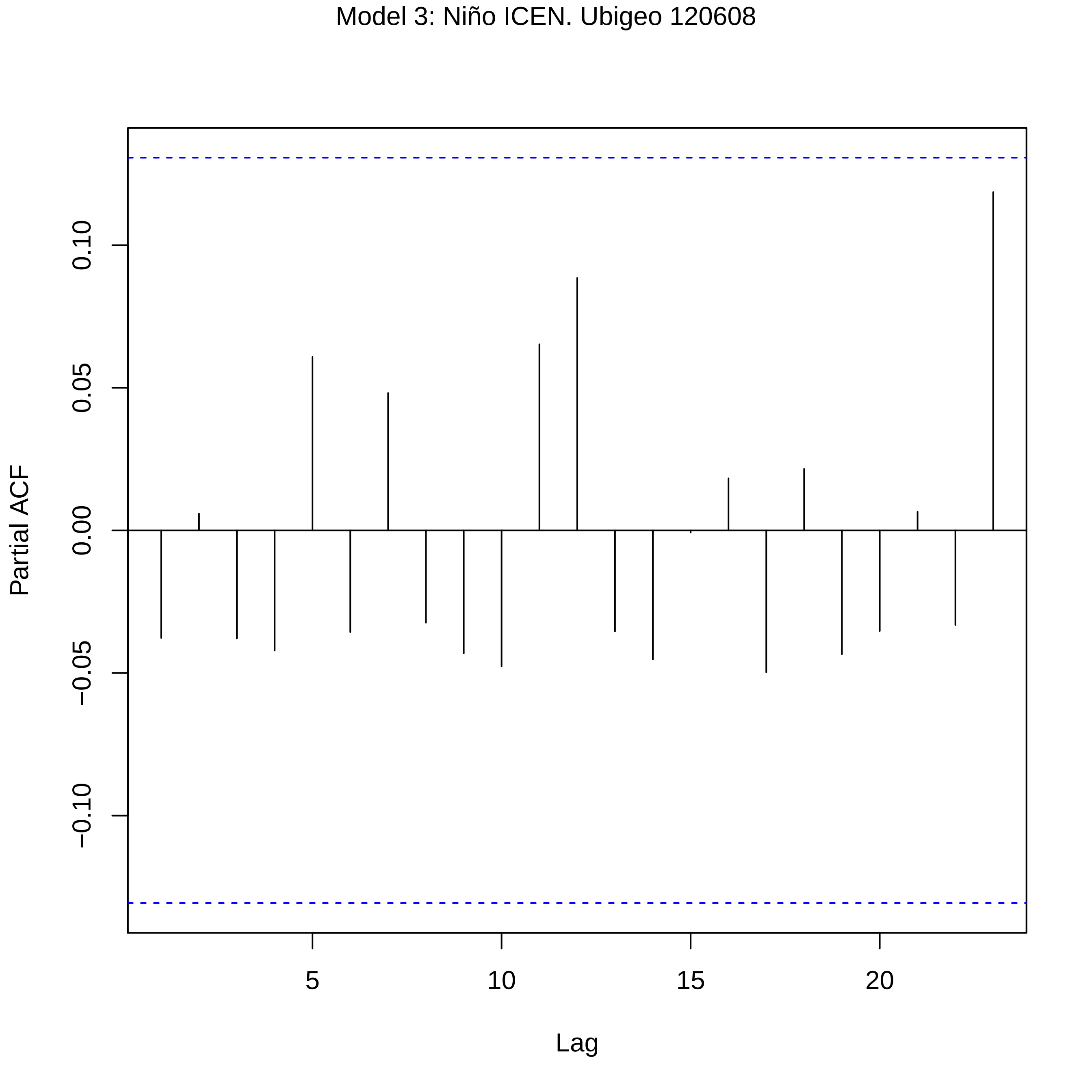

Model 3: Niño ICEN. Ubigeo 120608
Partial ACF
0.10
0.05
0.00
−0.05
−0.10
5
10
15
20
Lag

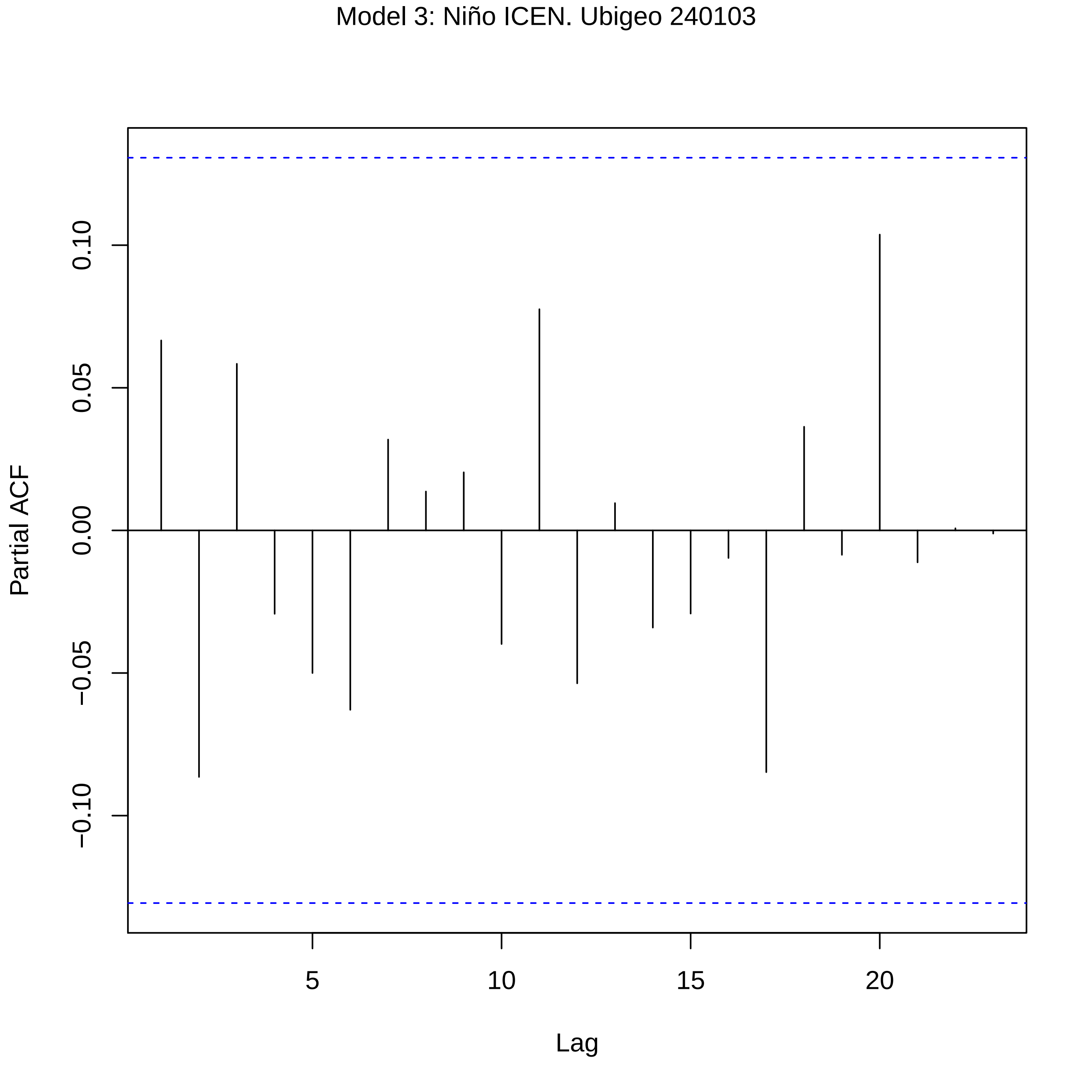
Model 3: Niño ICEN. Ubigeo 240103
Partial ACF
0.10
0.05
0.00
−0.05
−0.10
5
10
15
20
Lag

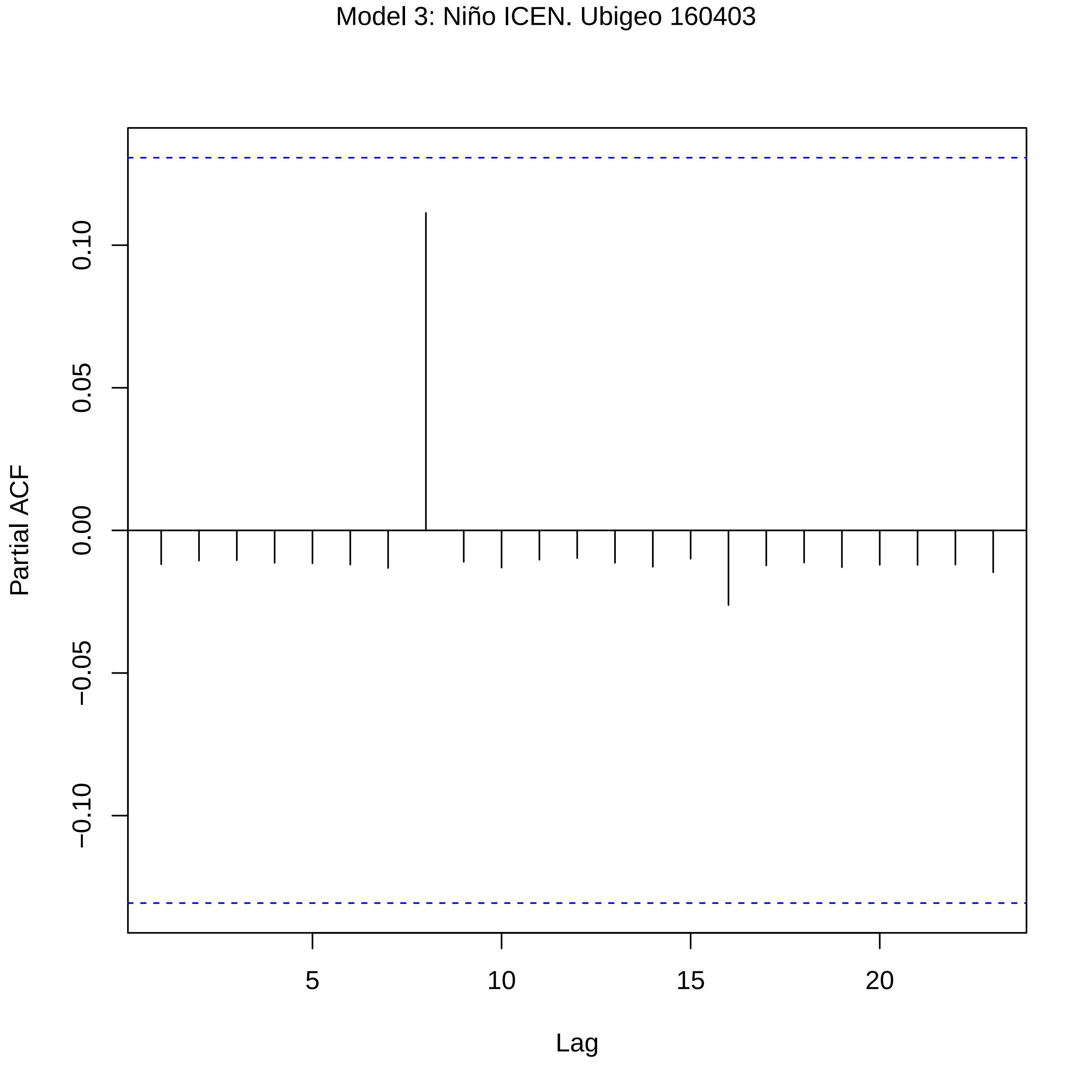
Model 3: Niño ICEN. Ubigeo 160403
Partial ACF
0.10
0.05
0.00
−0.05
−0.10
5
10
15
20
Lag

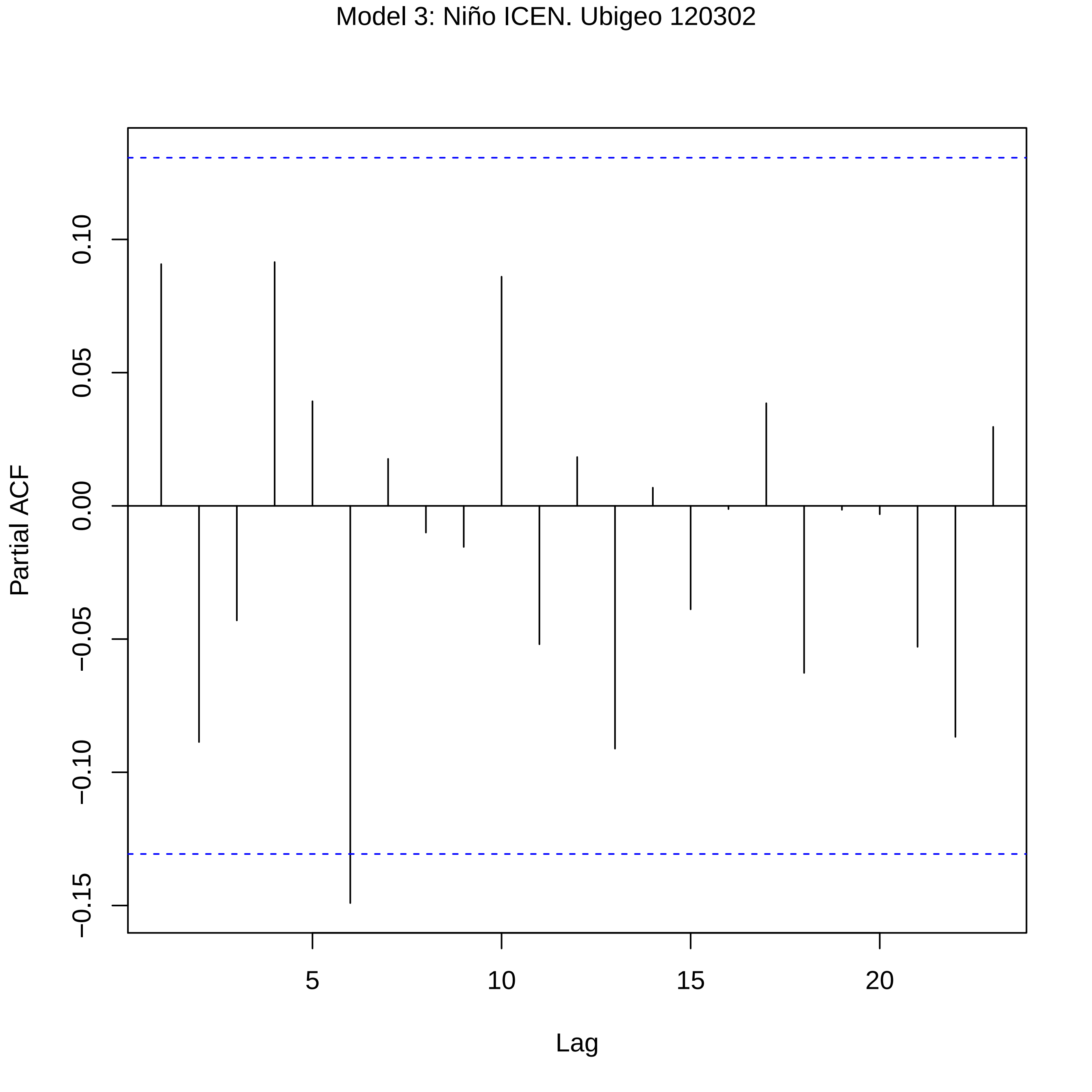
Model 3: Niño ICEN. Ubigeo 120302
Partial ACF
0.10
0.05
0.00
−0.05
−0.10
−0.15
5
10
15
20
Lag

Model 4: Niño ONI with region interaction. Ubigeo 220801

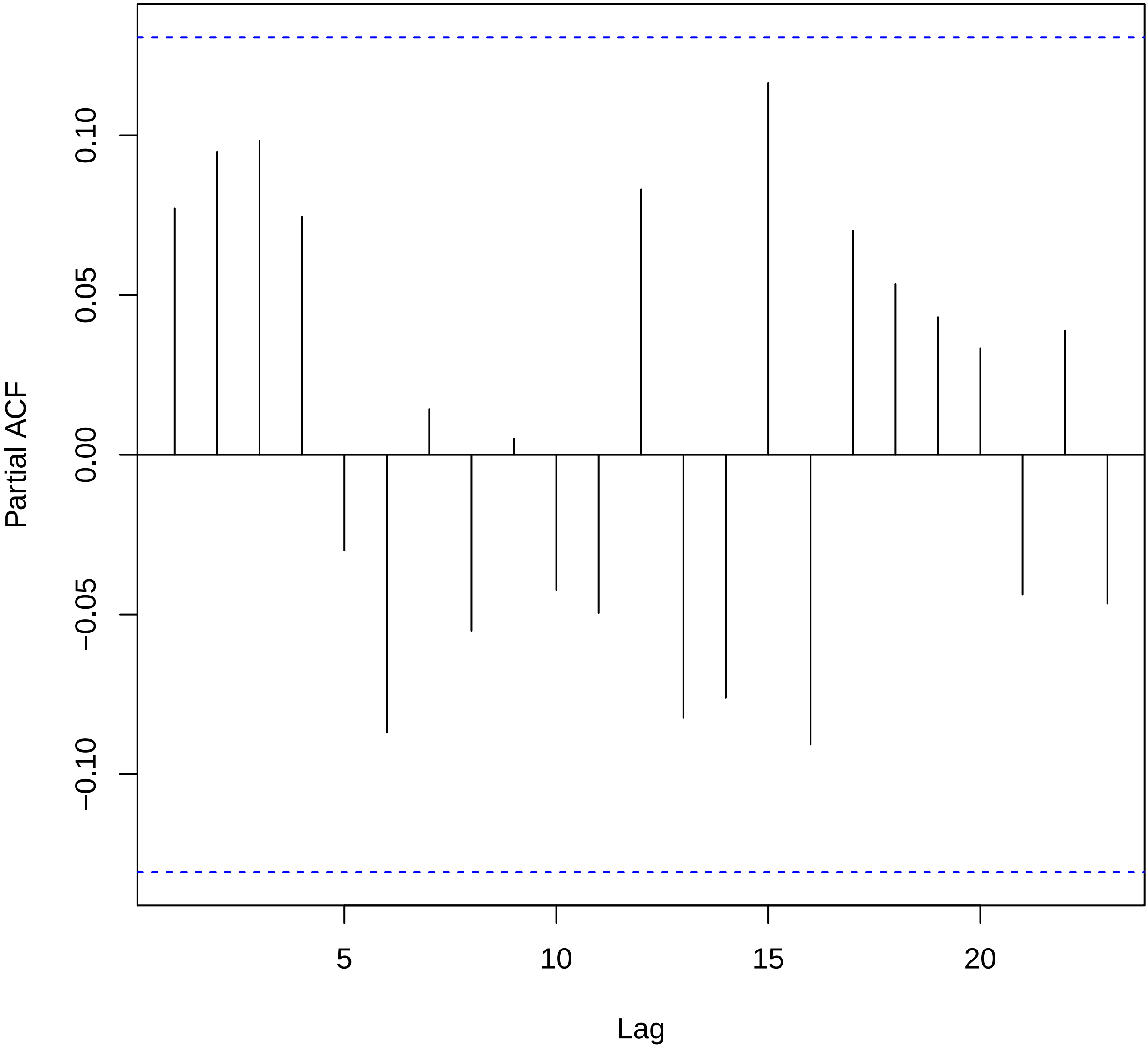

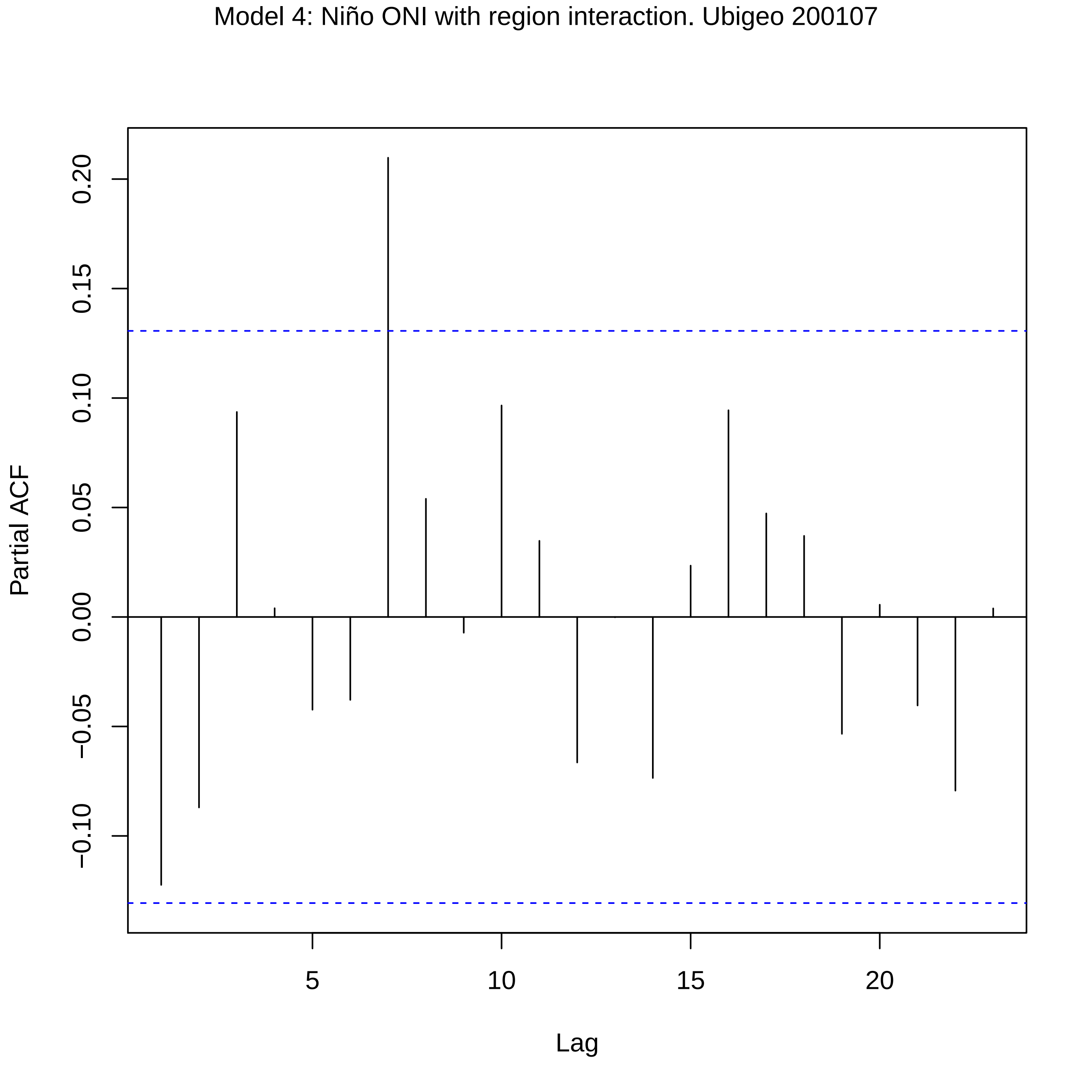
Model 4: Niño ONI with region interaction. Ubigeo 200107
Partial ACF
Lag
0.20
0.15
0.10
0.05
0.00
−0.05
−0.10
5
10
15
20

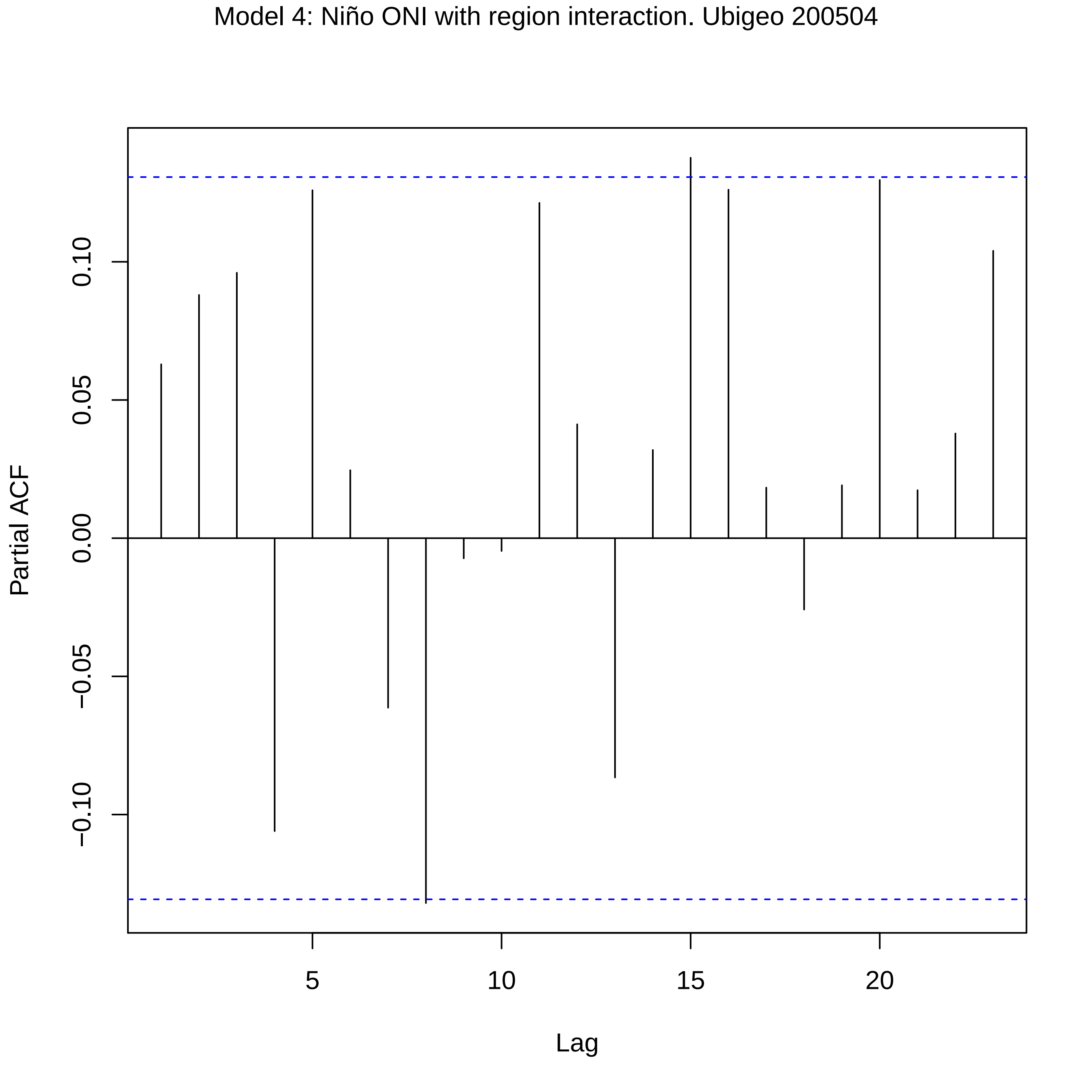

Model 4: Niño ONI with region interaction. Ubigeo 200504
Partial ACF
−0.10
−0.05
0.00
0.05
0.10
5
10
15
20
Lag

Model 4: Niño ONI with region interaction. Ubigeo 220603

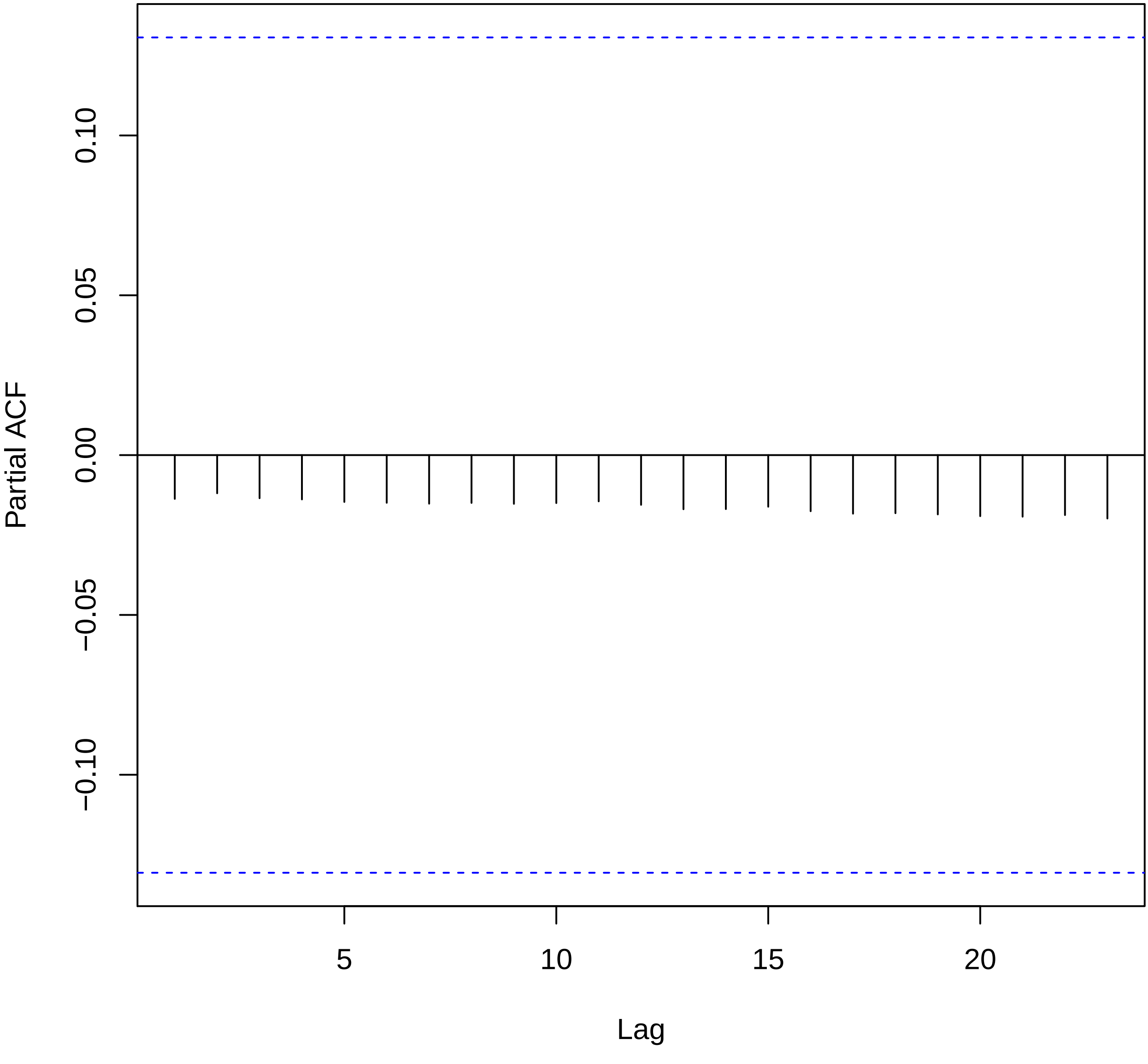

Model 4: Niño ONI with region interaction. Ubigeo 130202

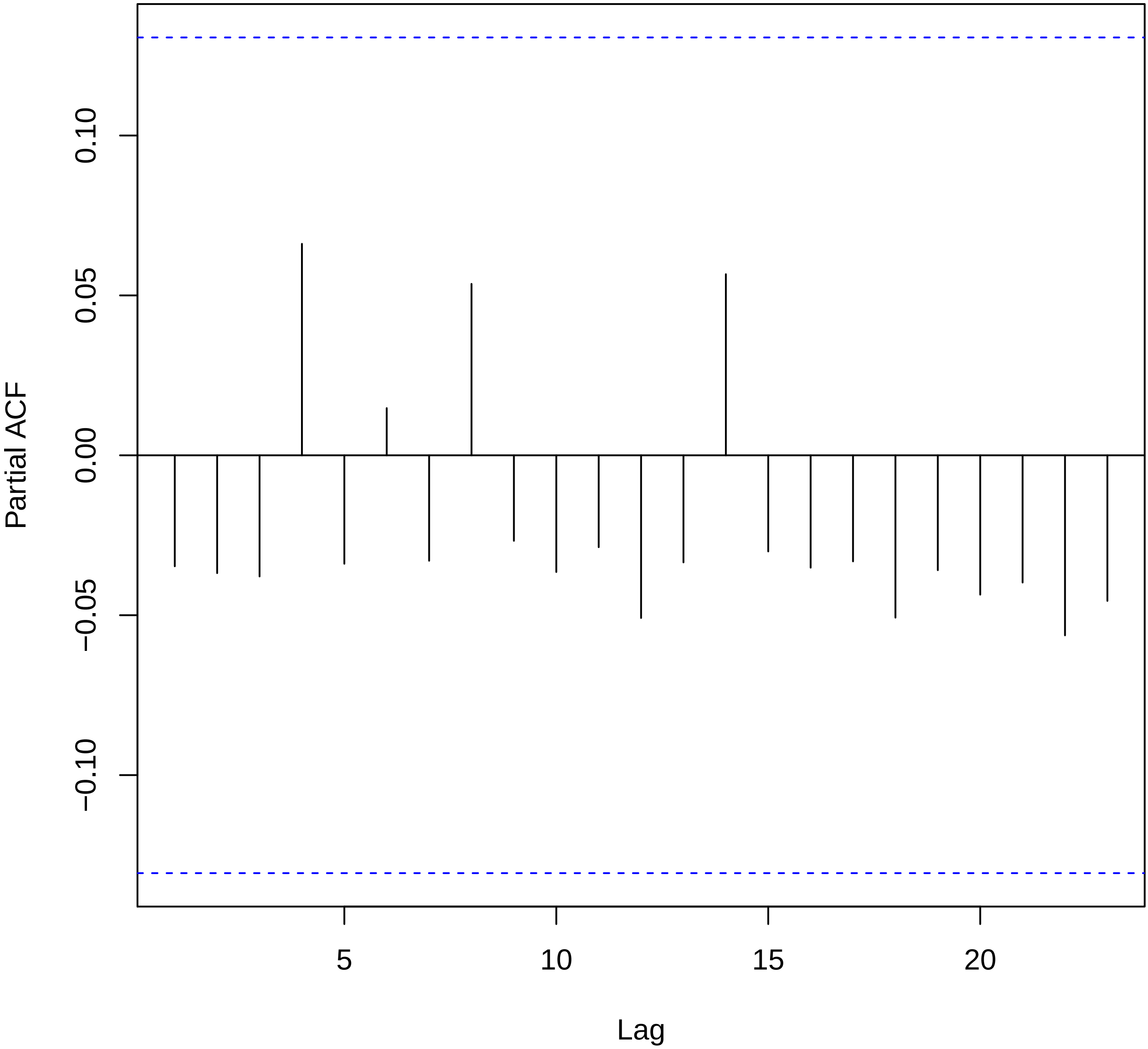

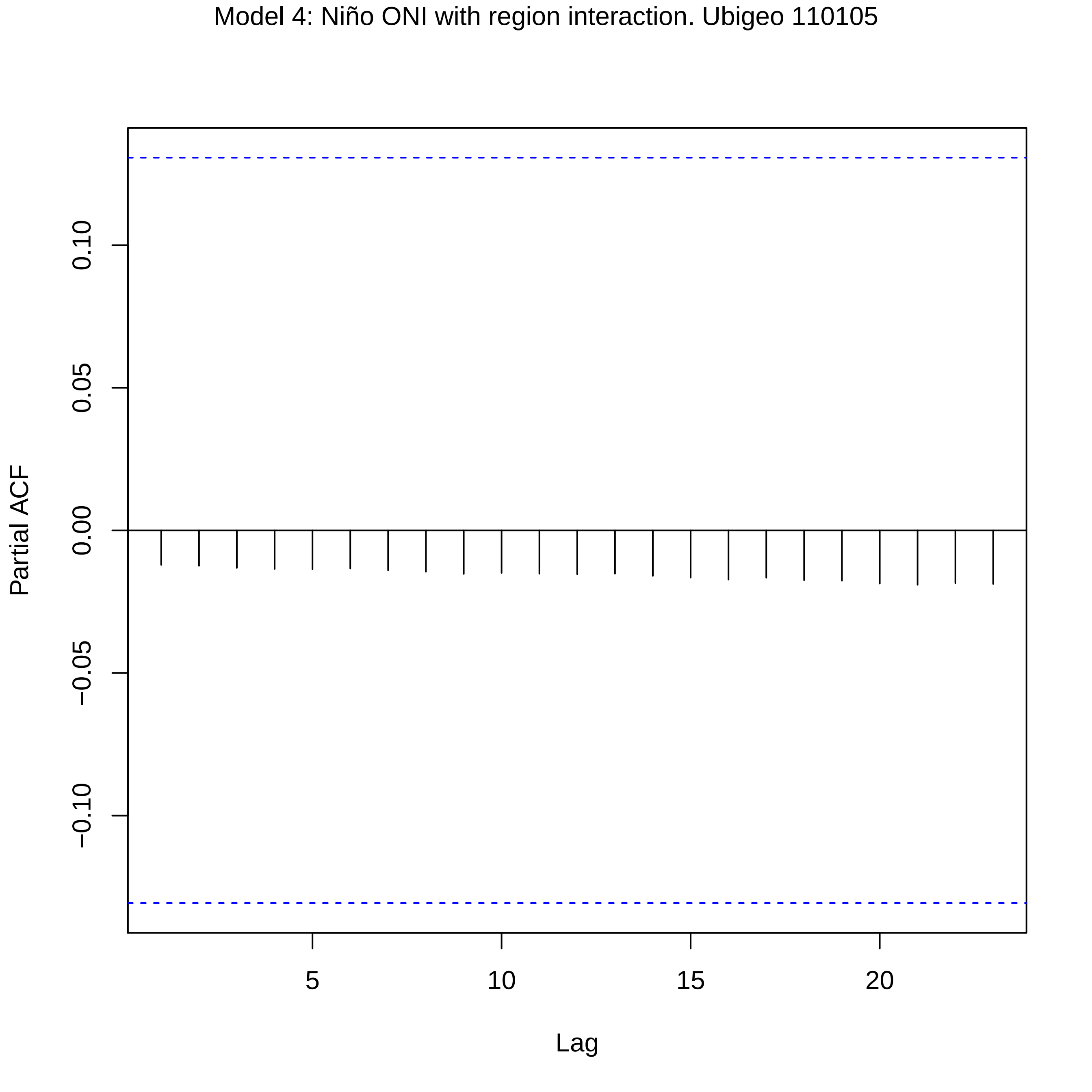
Model 4: Niño ONI with region interaction. Ubigeo 110105
Partial ACF
0.10
0.05
0.00
−0.05
−0.10
5
10
15
20
Lag

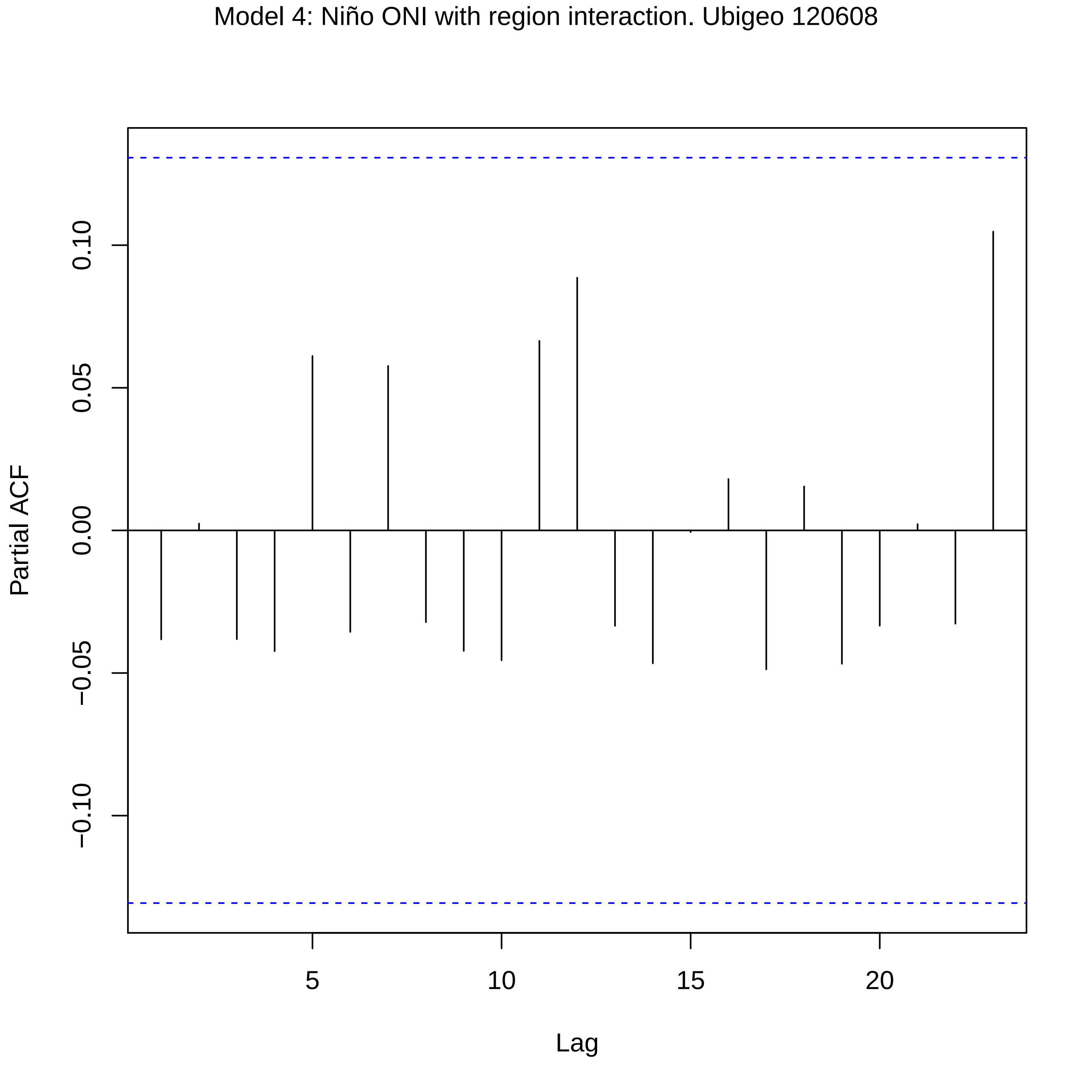
Model 4: Niño ONI with region interaction. Ubigeo 120608
Partial ACF
0.10
0.05
0.00
−0.05
−0.10
5
10
15
20
Lag

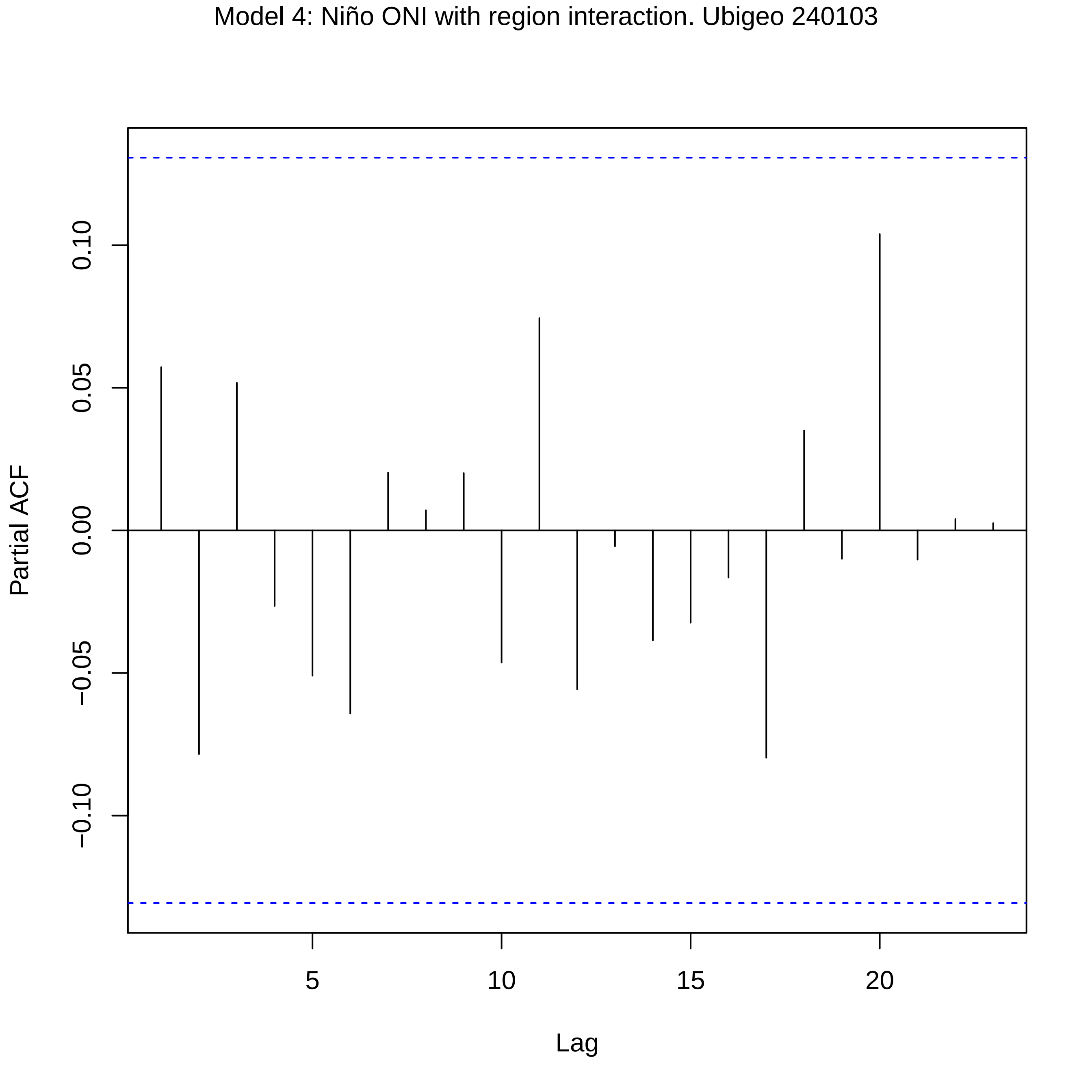
Model 4: Niño ONI with region interaction. Ubigeo 240103
Partial ACF
0.10
0.05
0.00
−0.05
−0.10
5
10
15
20
Lag

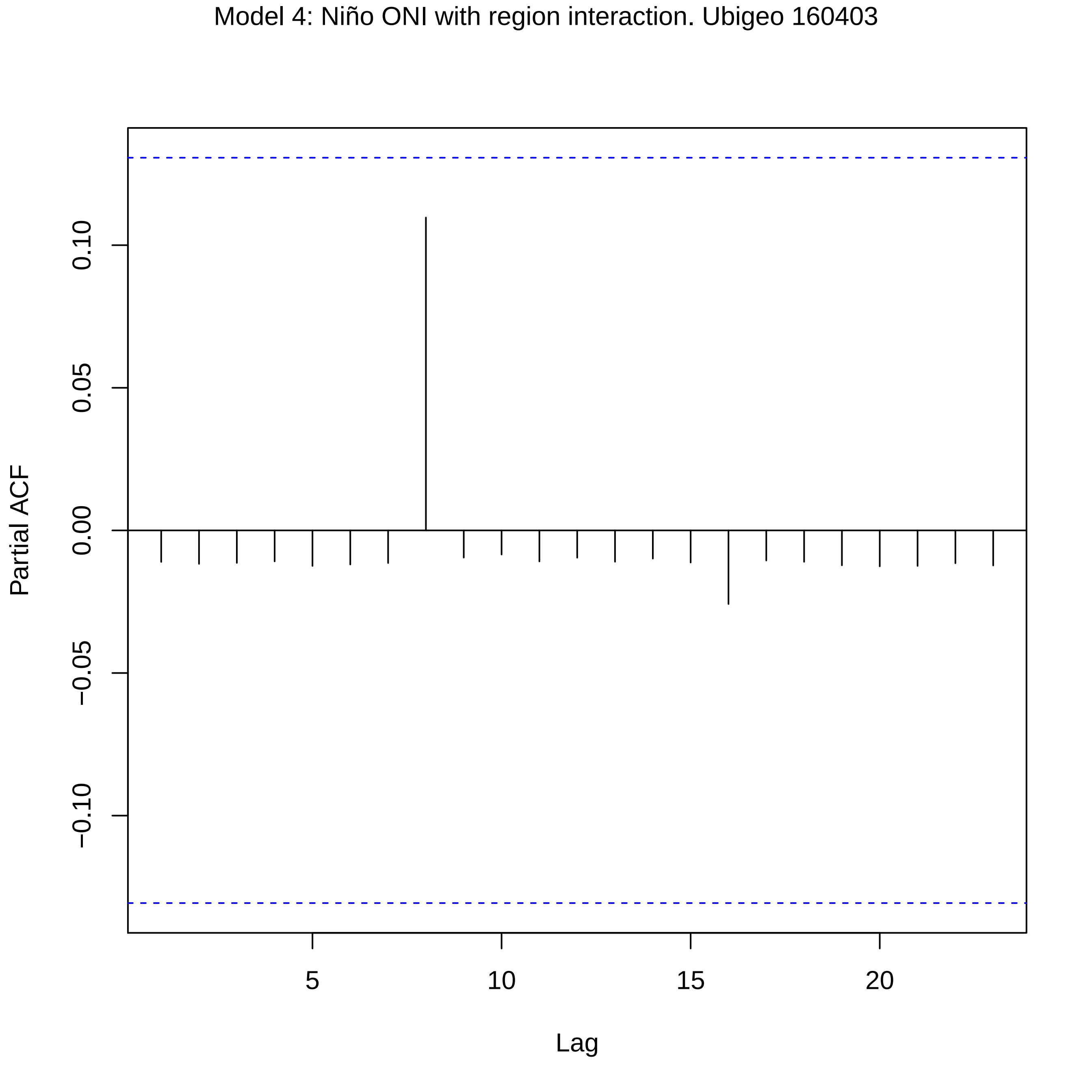
Model 4: Niño ONI with region interaction. Ubigeo 160403
Partial ACF
0.10
0.05
0.00
−0.05
−0.10
5
10
15
20
Lag

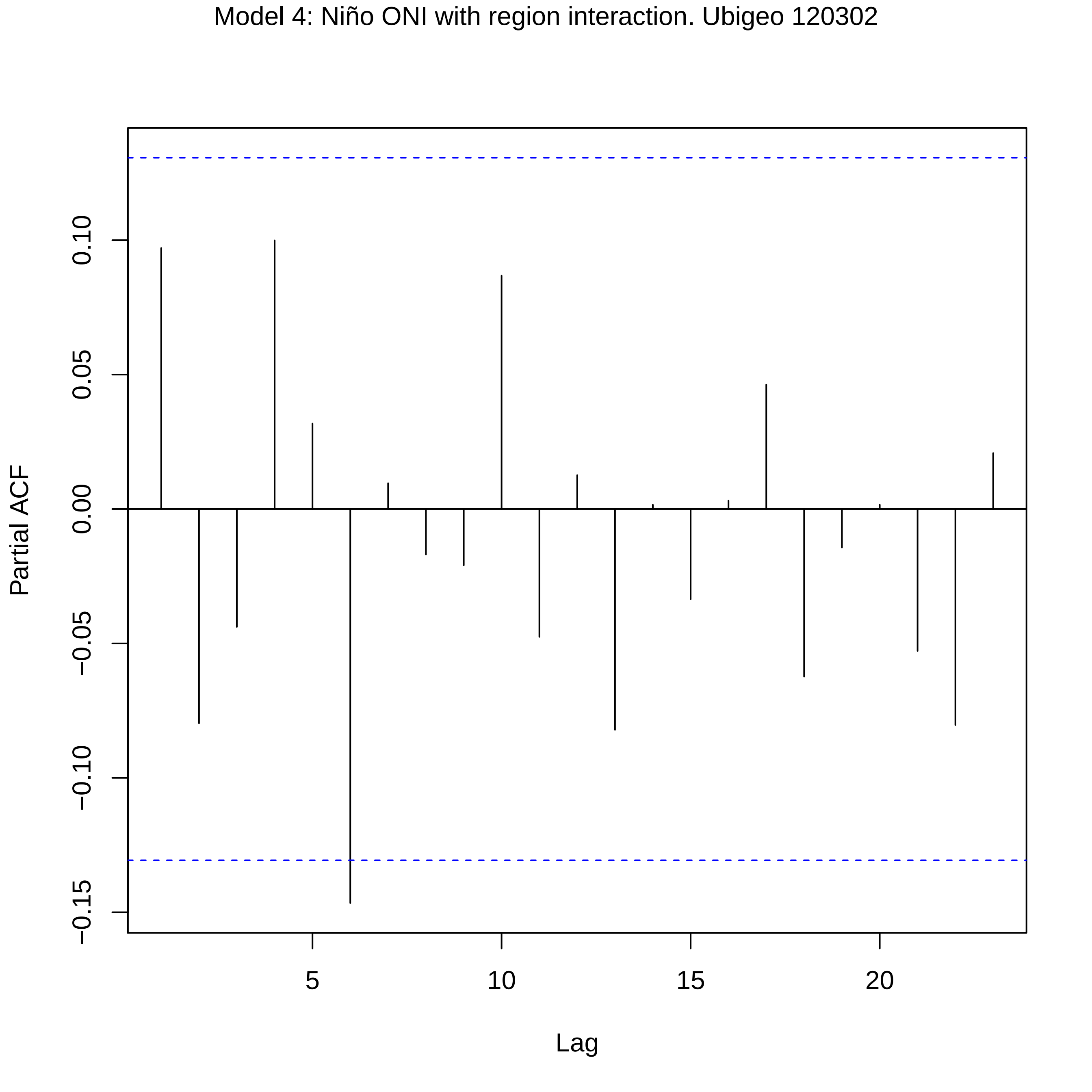
Model 4: Niño ONI with region interaction. Ubigeo 120302
Partial ACF
0.10
0.05
0.00
−0.05
−0.10
−0.15
5
10
15
20
Lag

Model 5: Niño ICEN with region interaction. Ubigeo 220801

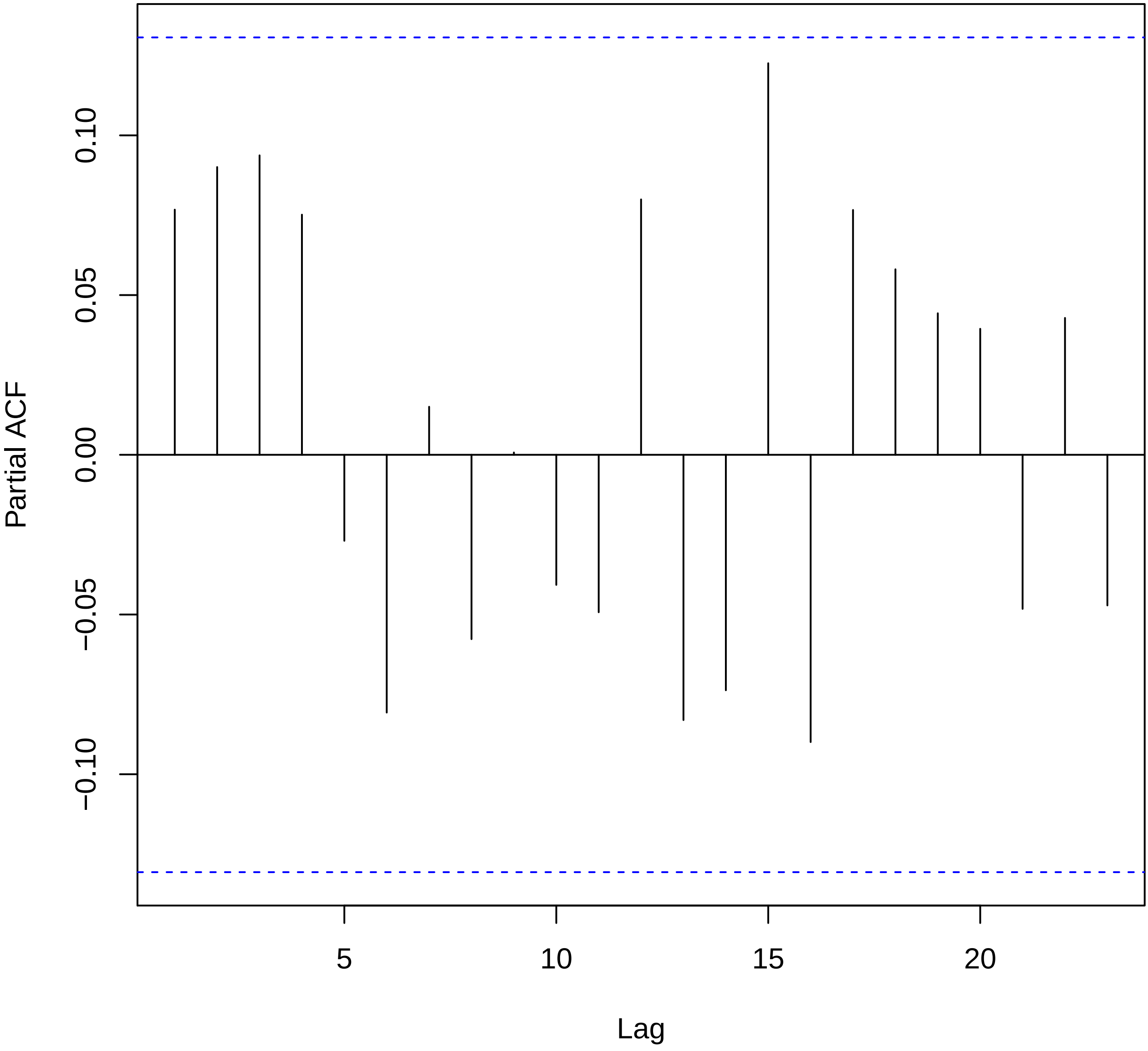

Model 5: Niño ICEN with region interaction. Ubigeo 200107

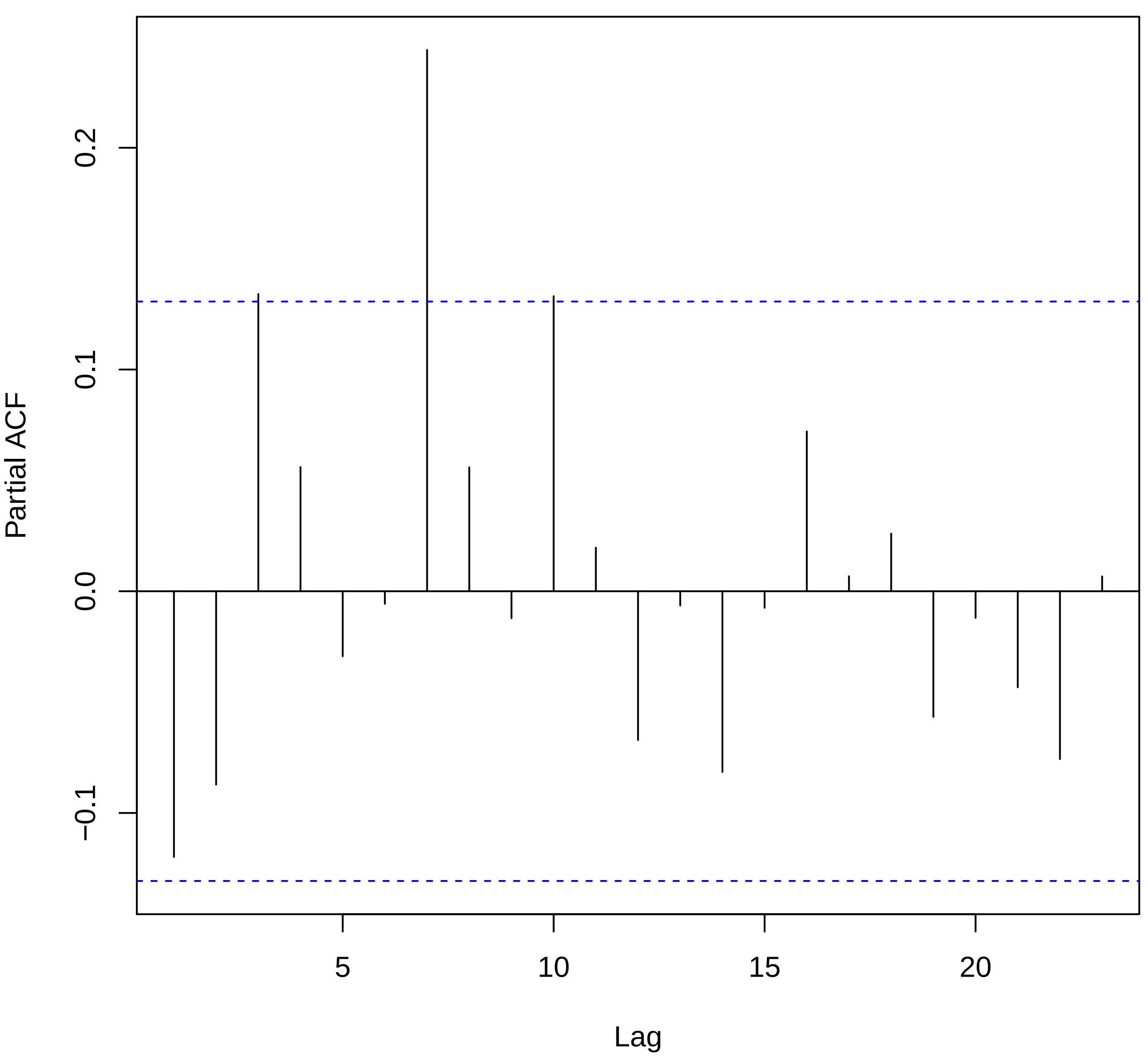

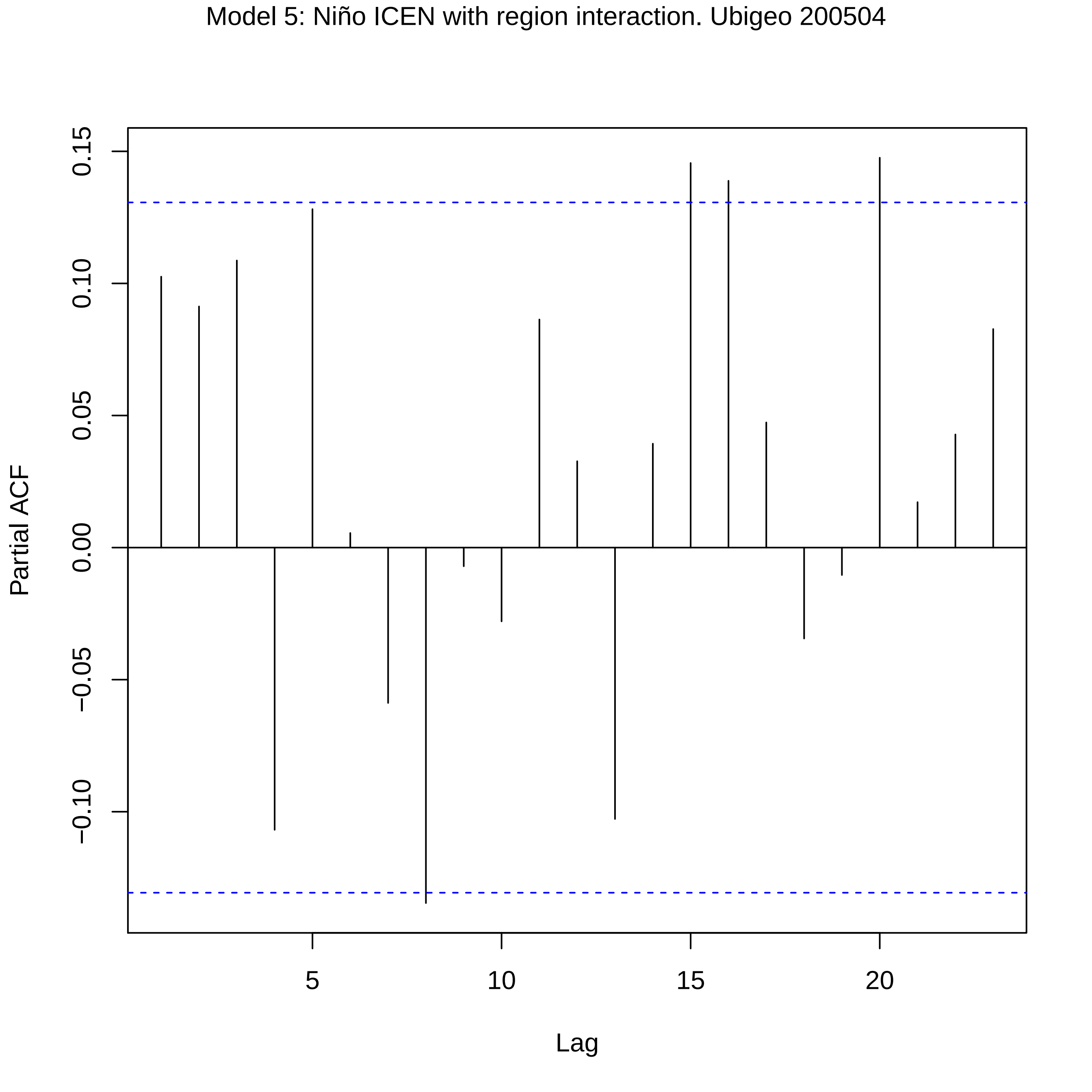
Model 5: Niño ICEN with region interaction. Ubigeo 200504
Partial ACF
0.15
0.10
0.05
0.00
−0.05
−0.10
5
10
15
20
Lag

Model 5: Niño ICEN with region interaction. Ubigeo 220603

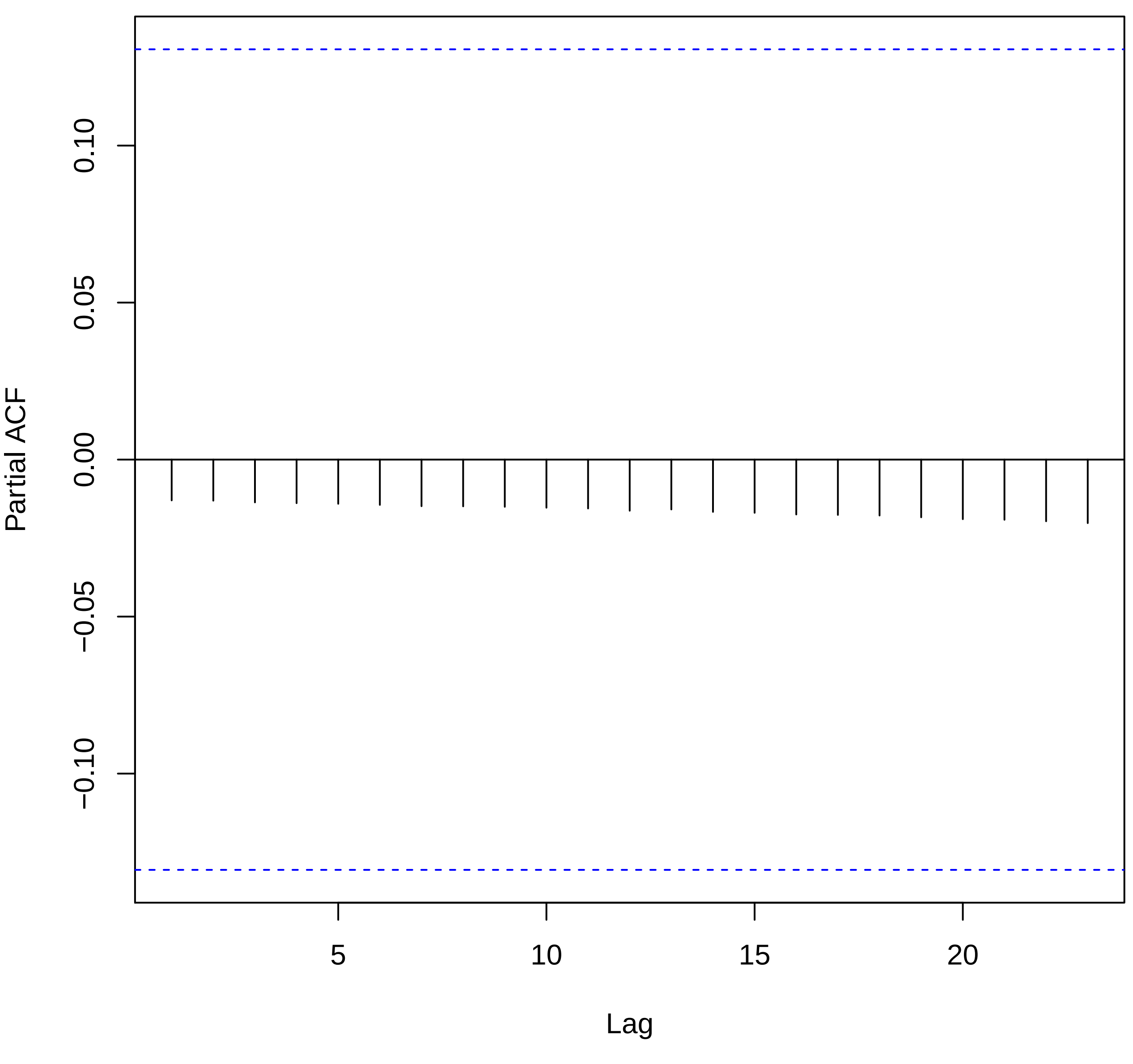

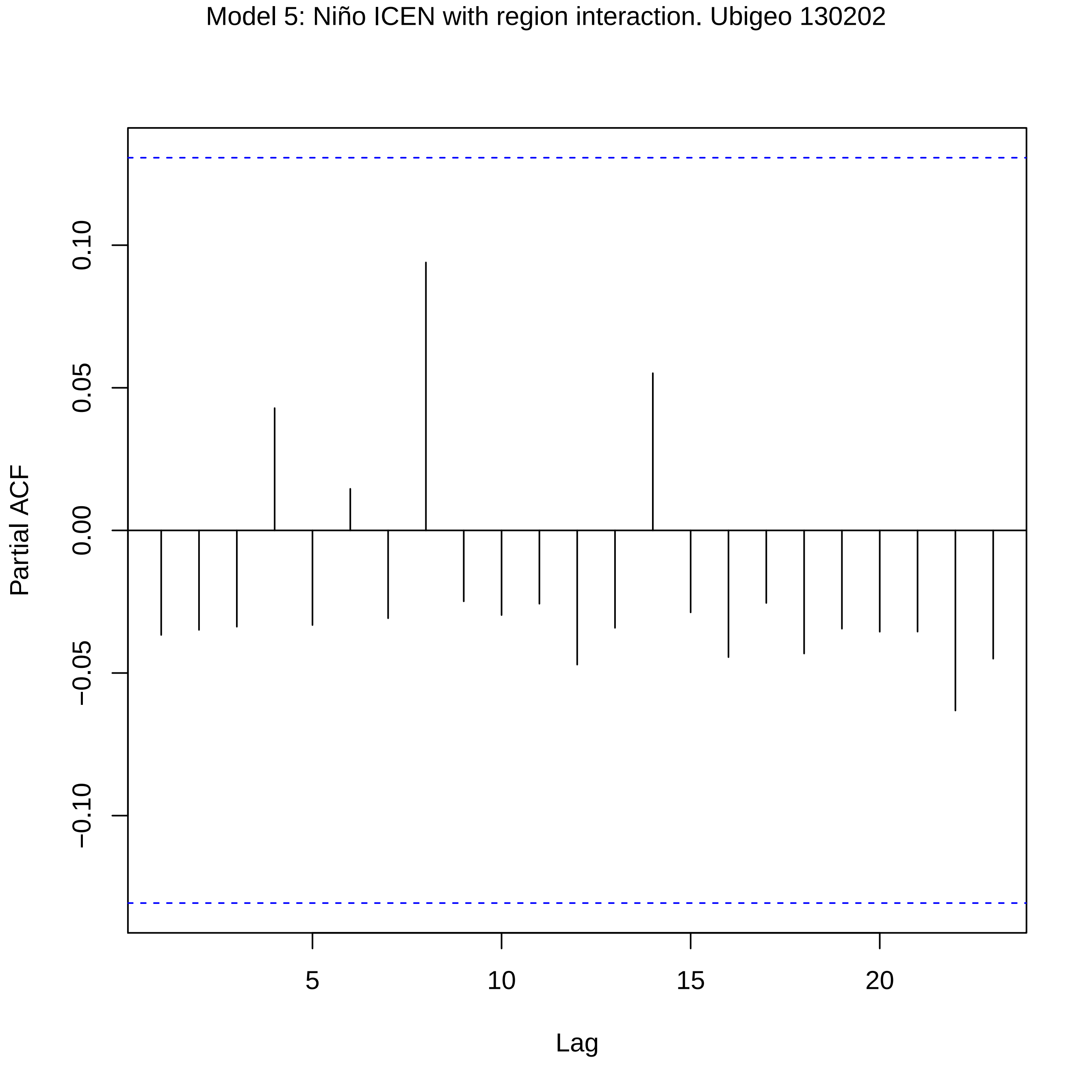
Model 5: Niño ICEN with region interaction. Ubigeo 130202
Partial ACF
0.10
0.05
0.00
−0.05
−0.10
5
10
15
20
Lag

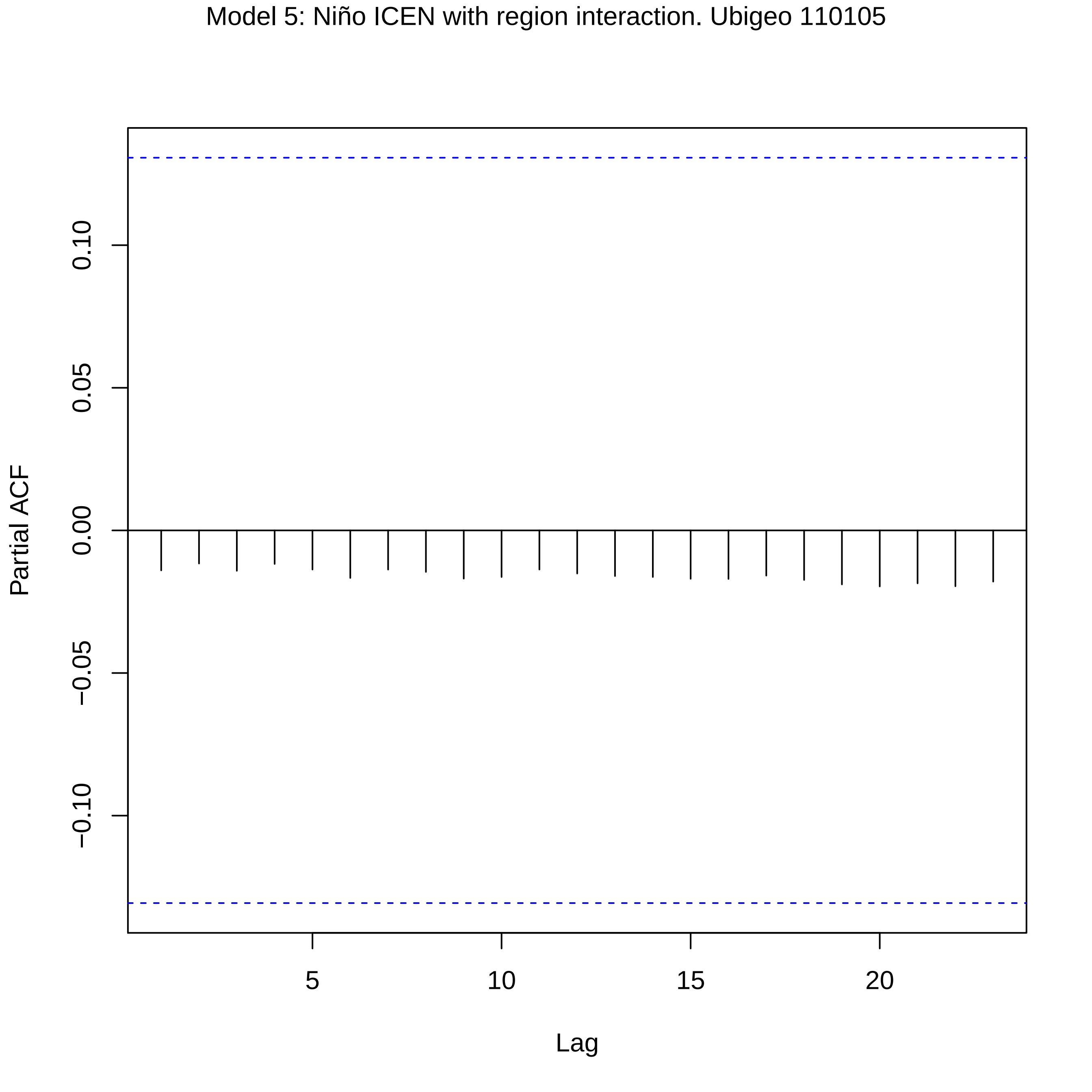
Model 5: Niño ICEN with region interaction. Ubigeo 110105
Partial ACF
0.10
0.05
0.00
−0.05
−0.10
5
10
15
20
Lag

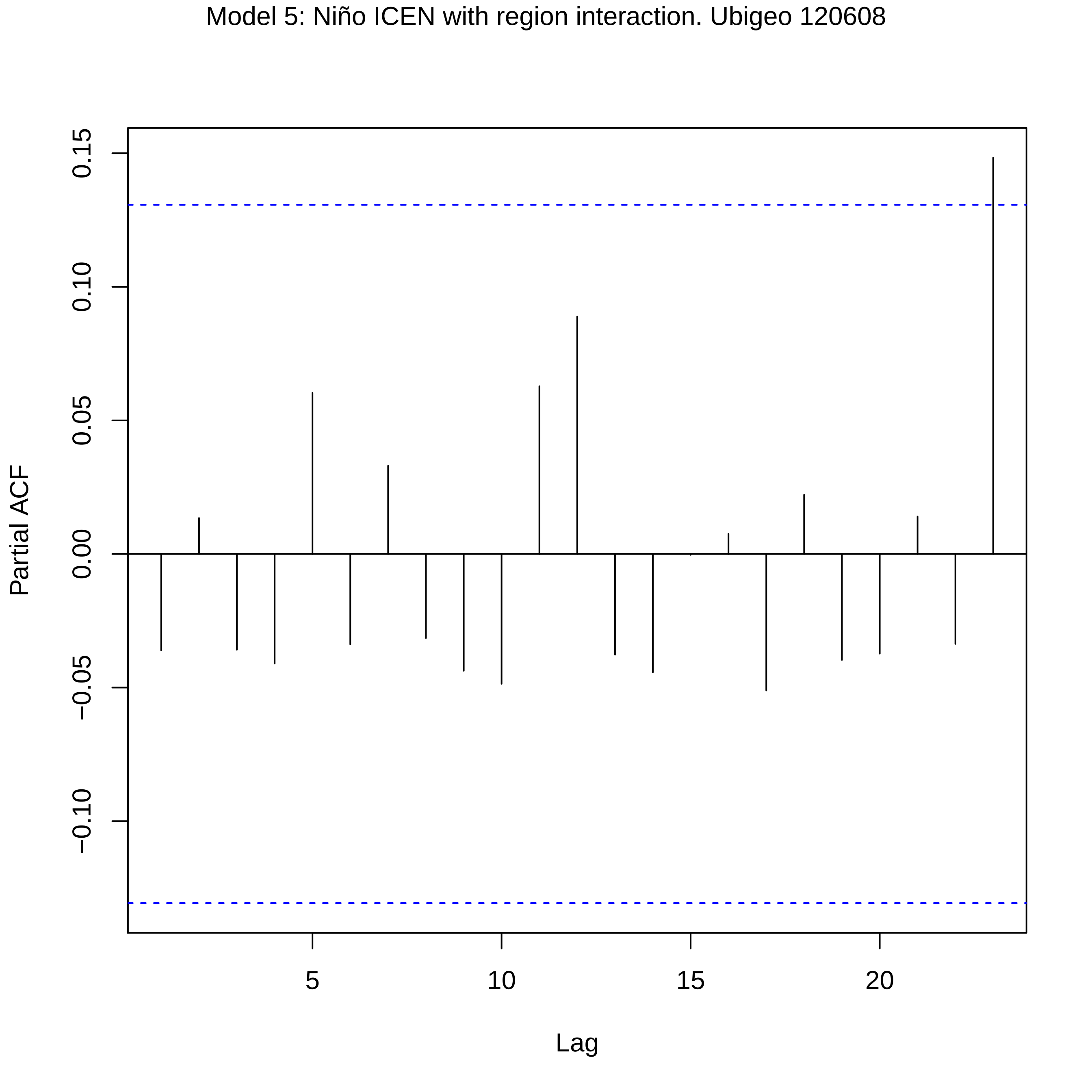

Model 5: Niño ICEN with region interaction. Ubigeo 120608
Partial ACF
0.15
0.10
0.05
0.00
−0.05
−0.10
5
10
15
20
Lag

Model 5: Niño ICEN with region interaction. Ubigeo 240103

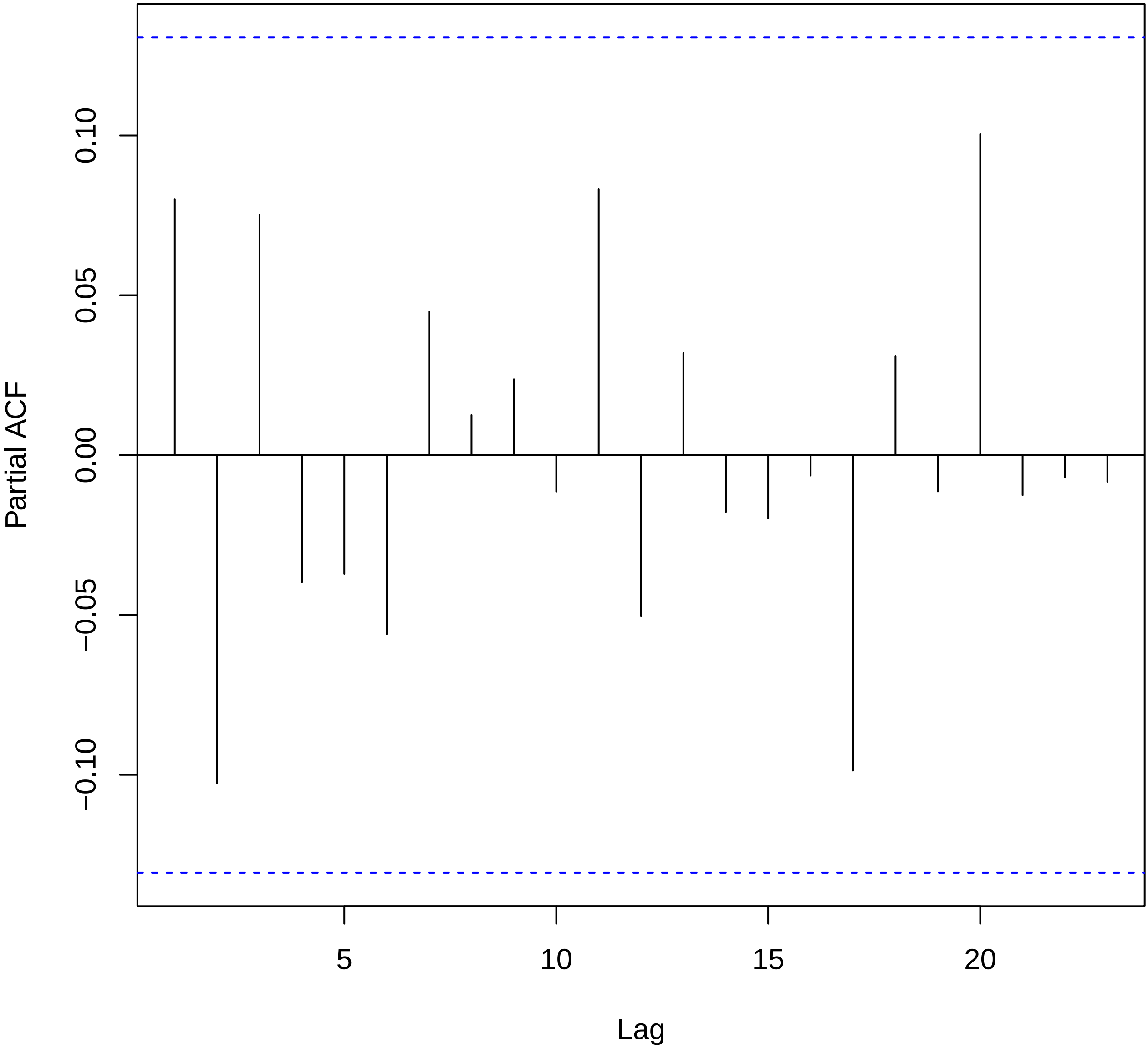

Model 5: Niño ICEN with region interaction. Ubigeo 160403

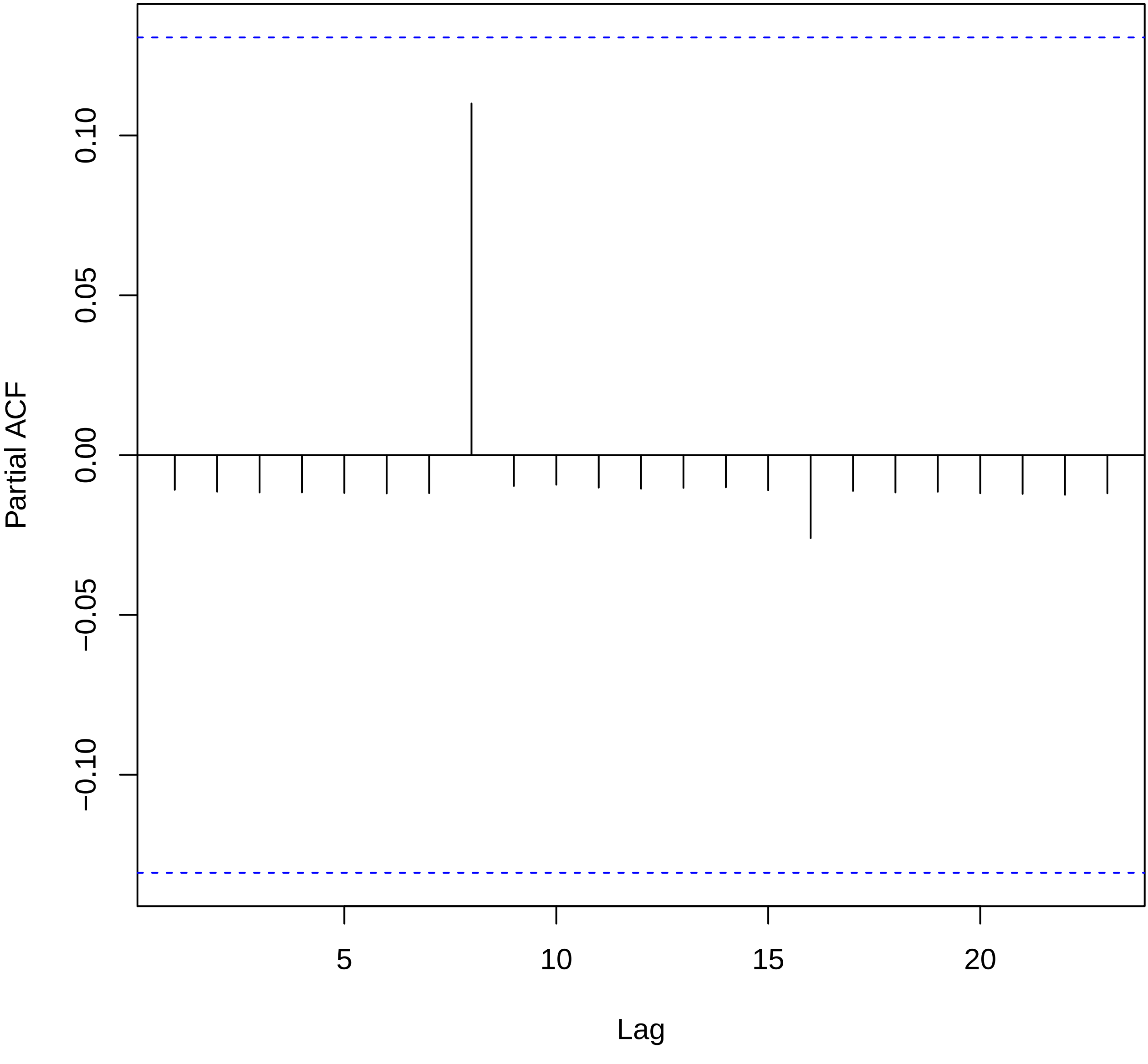

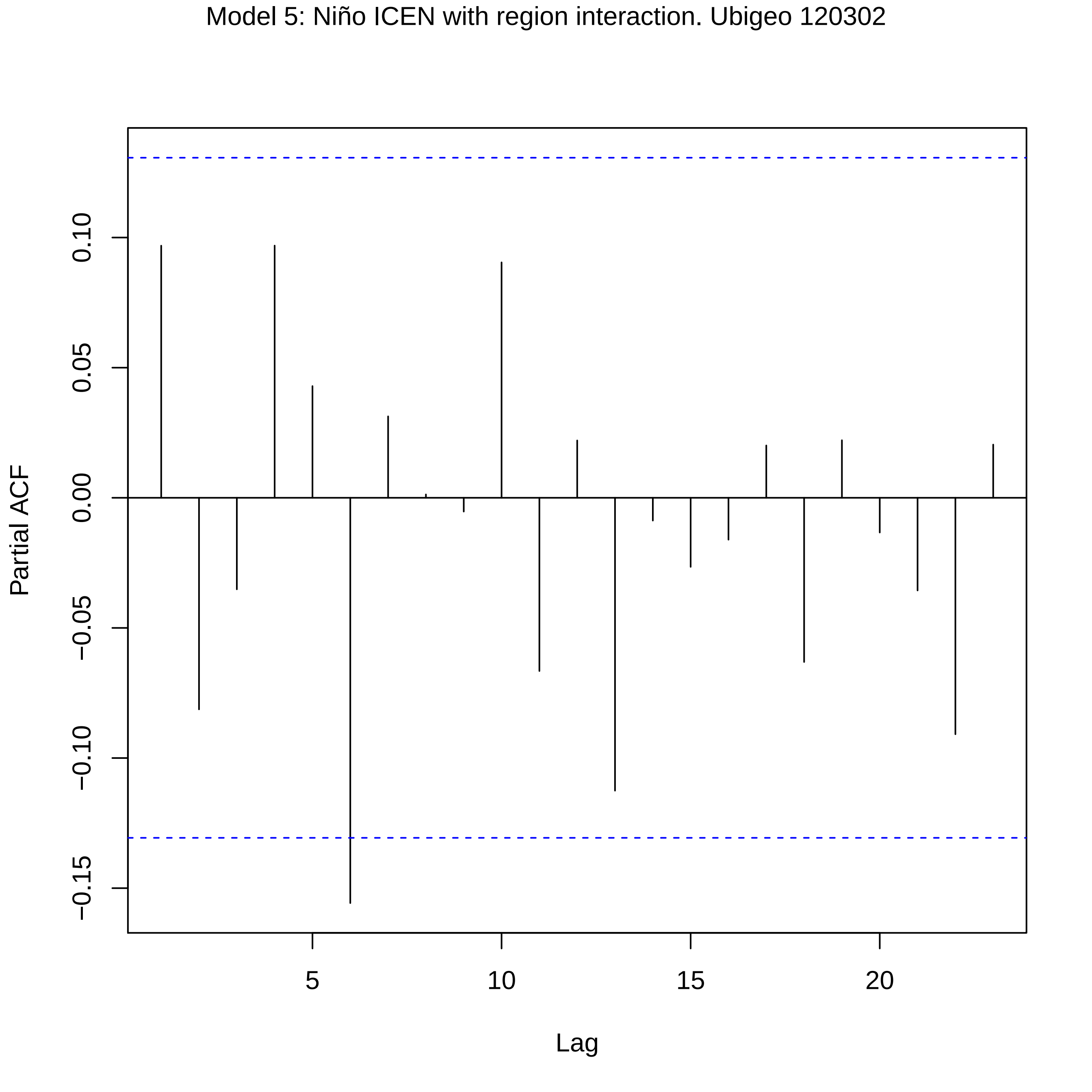

Model 5: Niño ICEN with region interaction. Ubigeo 120302
Partial ACF
0.10
0.05
0.00
−0.05
−0.10
−0.15
5
10
15
20
Lag

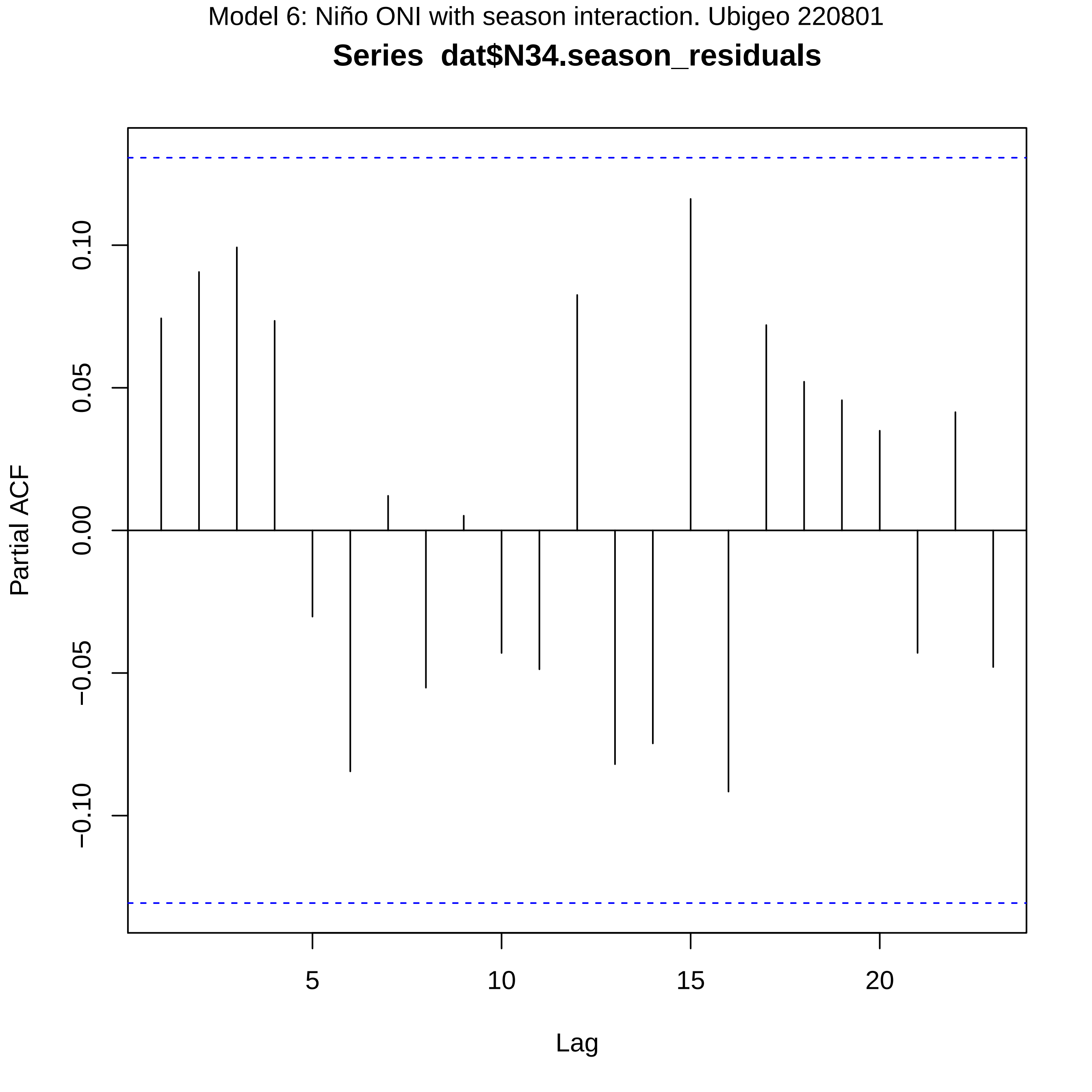
Model 6: Niño ONI with season interaction. Ubigeo 220801
Series dat$N34.season_residuals
Partial ACF
0.10
0.05
0.00
−0.05
−0.10
5
10
15
20
Lag

Model 6: Niño ONI with season interaction. Ubigeo 200107

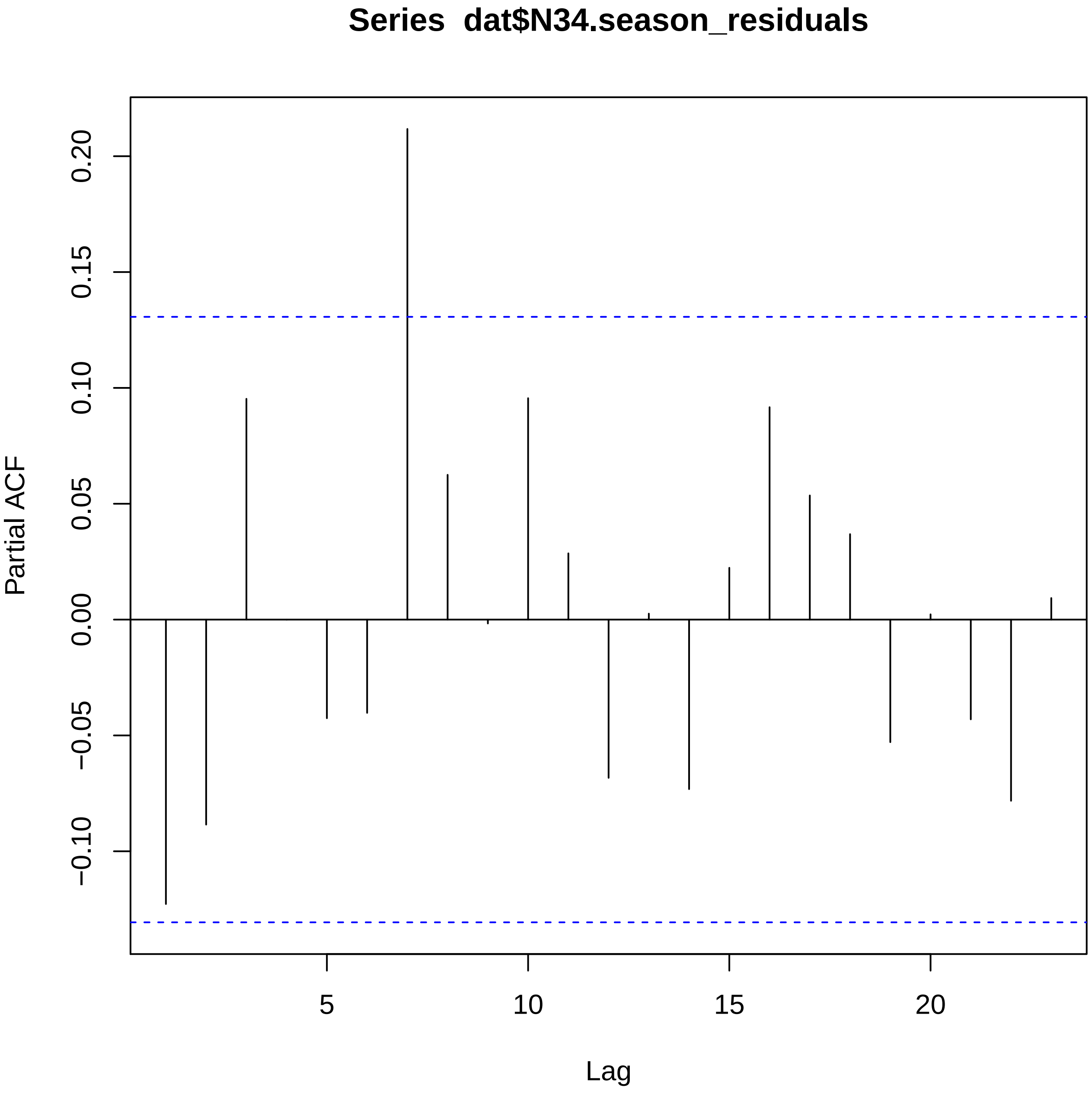

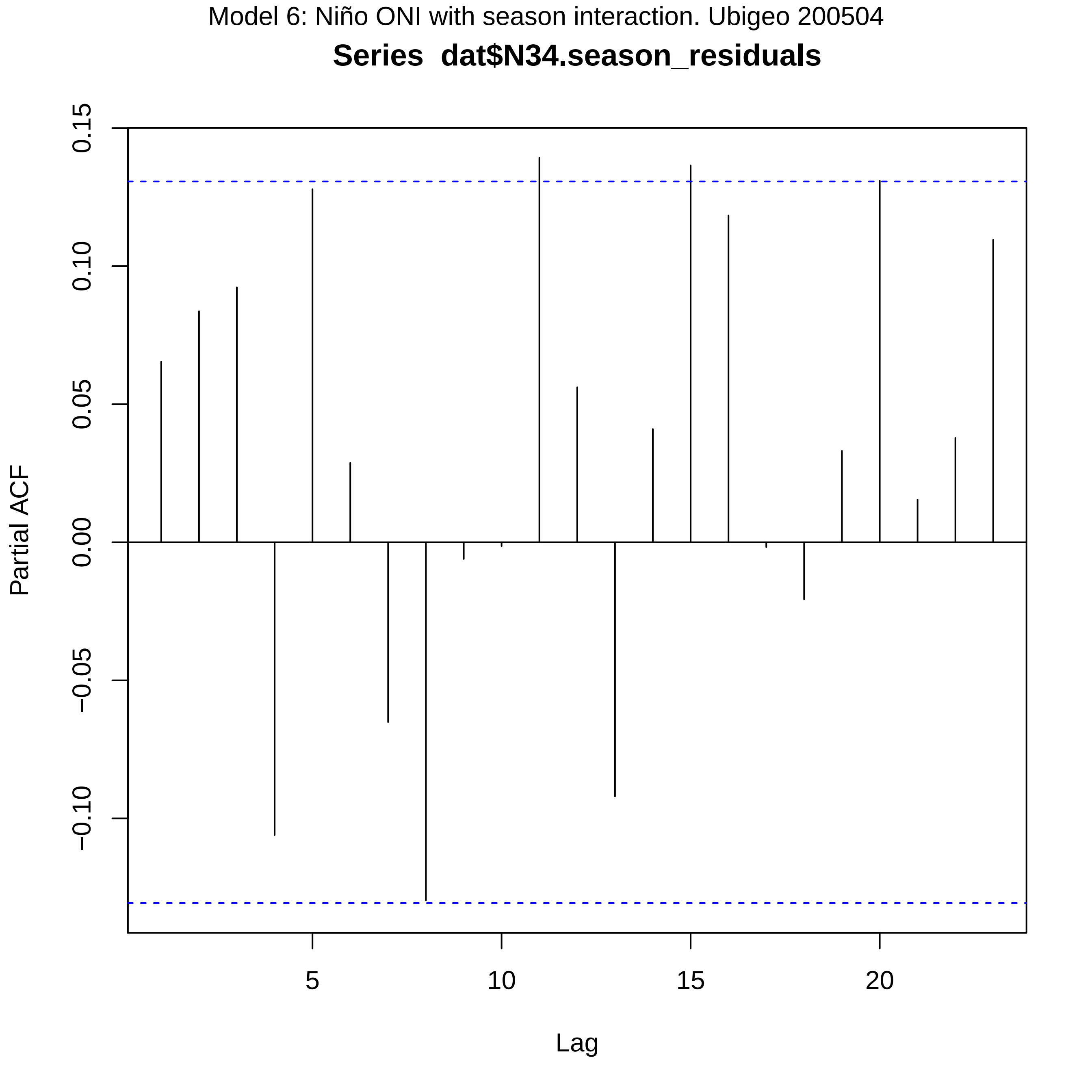
Model 6: Niño ONI with season interaction. Ubigeo 200504
Series dat$N34.season_residuals
Partial ACF
0.15
0.10
0.05
0.00
−0.05
−0.10
5
10
15
20
Lag

Model 6: Niño ONI with season interaction. Ubigeo 220603

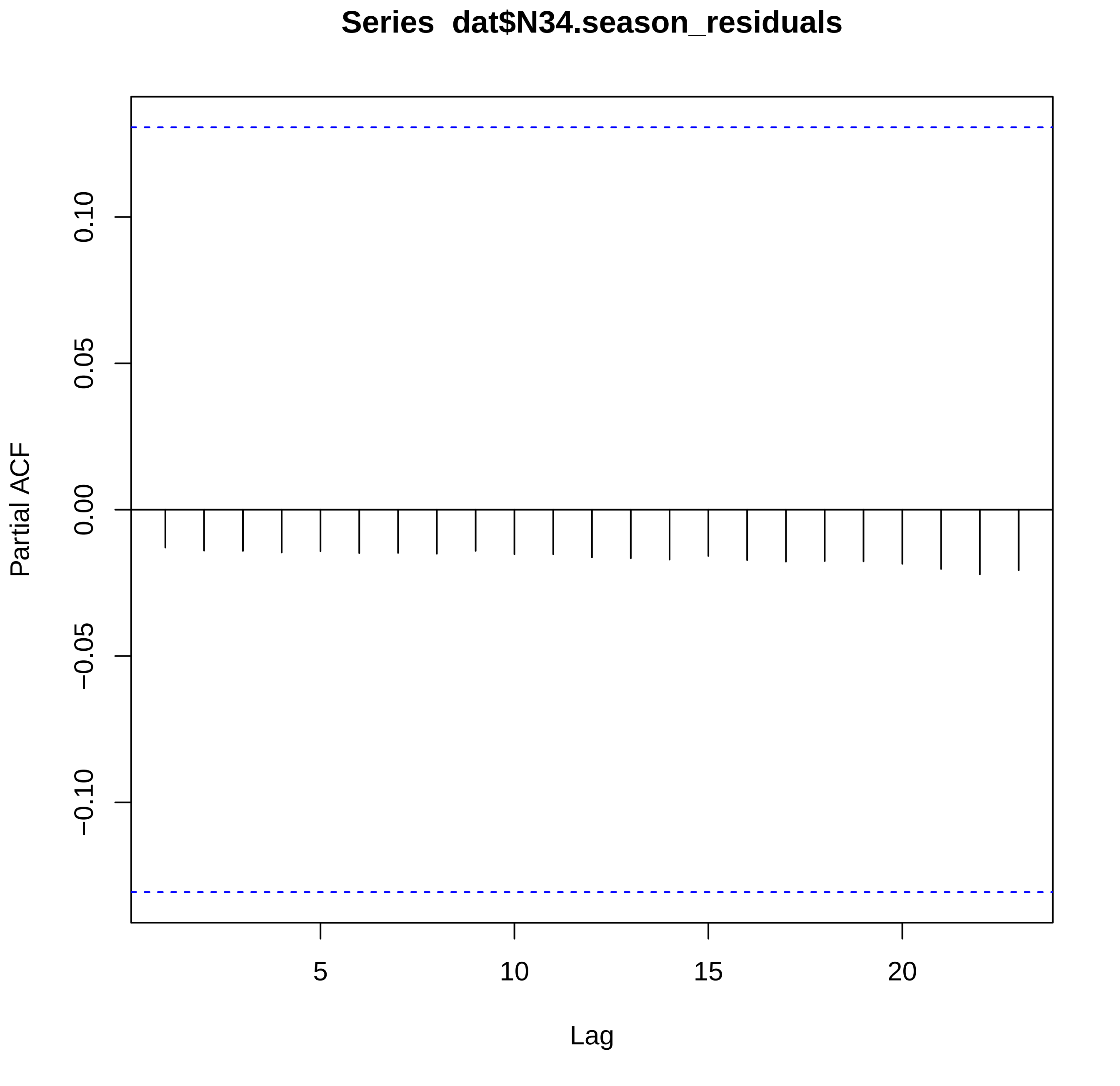

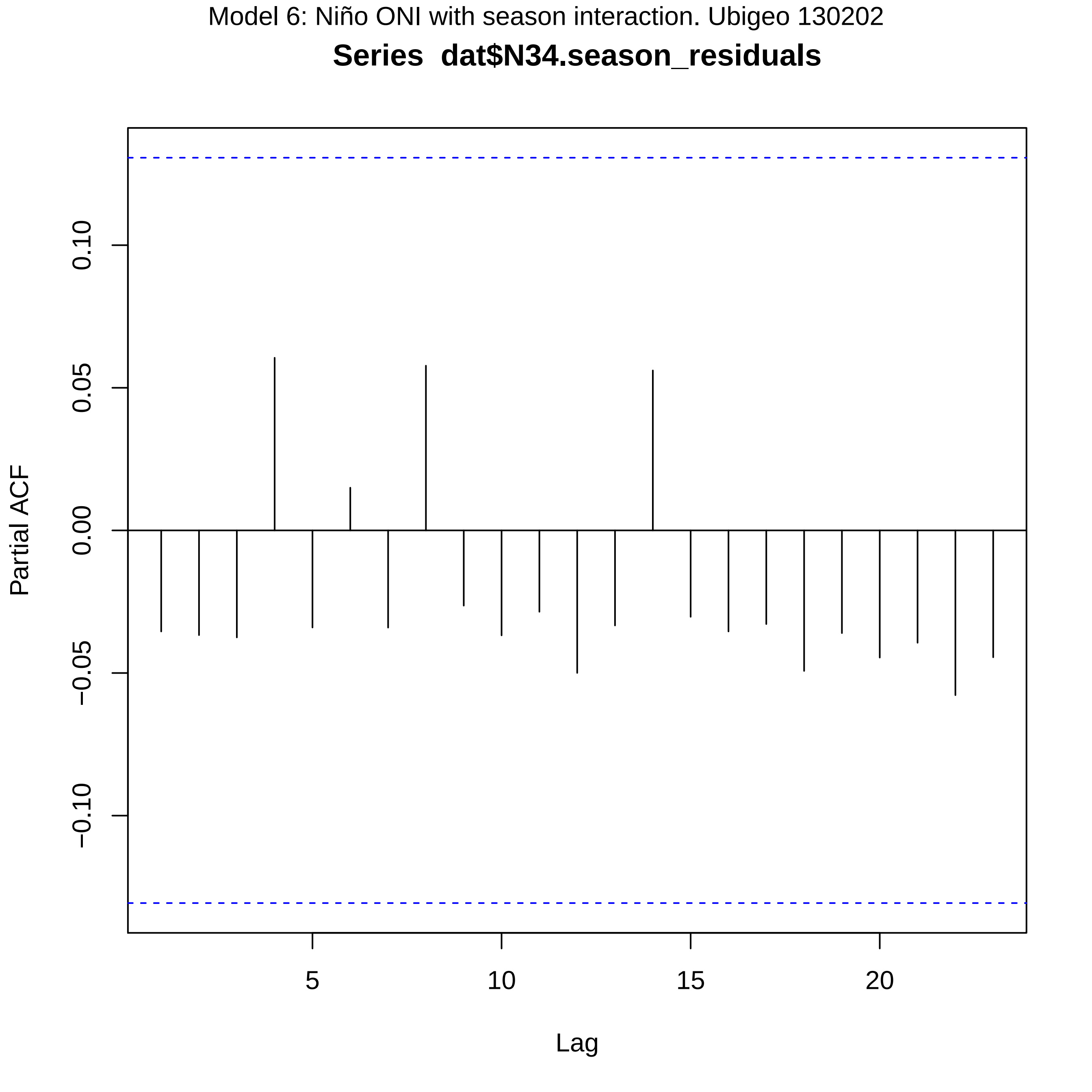
Model 6: Niño ONI with season interaction. Ubigeo 130202
Series dat$N34.season_residuals
Partial ACF
0.10
0.05
0.00
−0.05
−0.10
5
10
15
20
Lag

Model 6: Niño ONI with season interaction. Ubigeo 110105

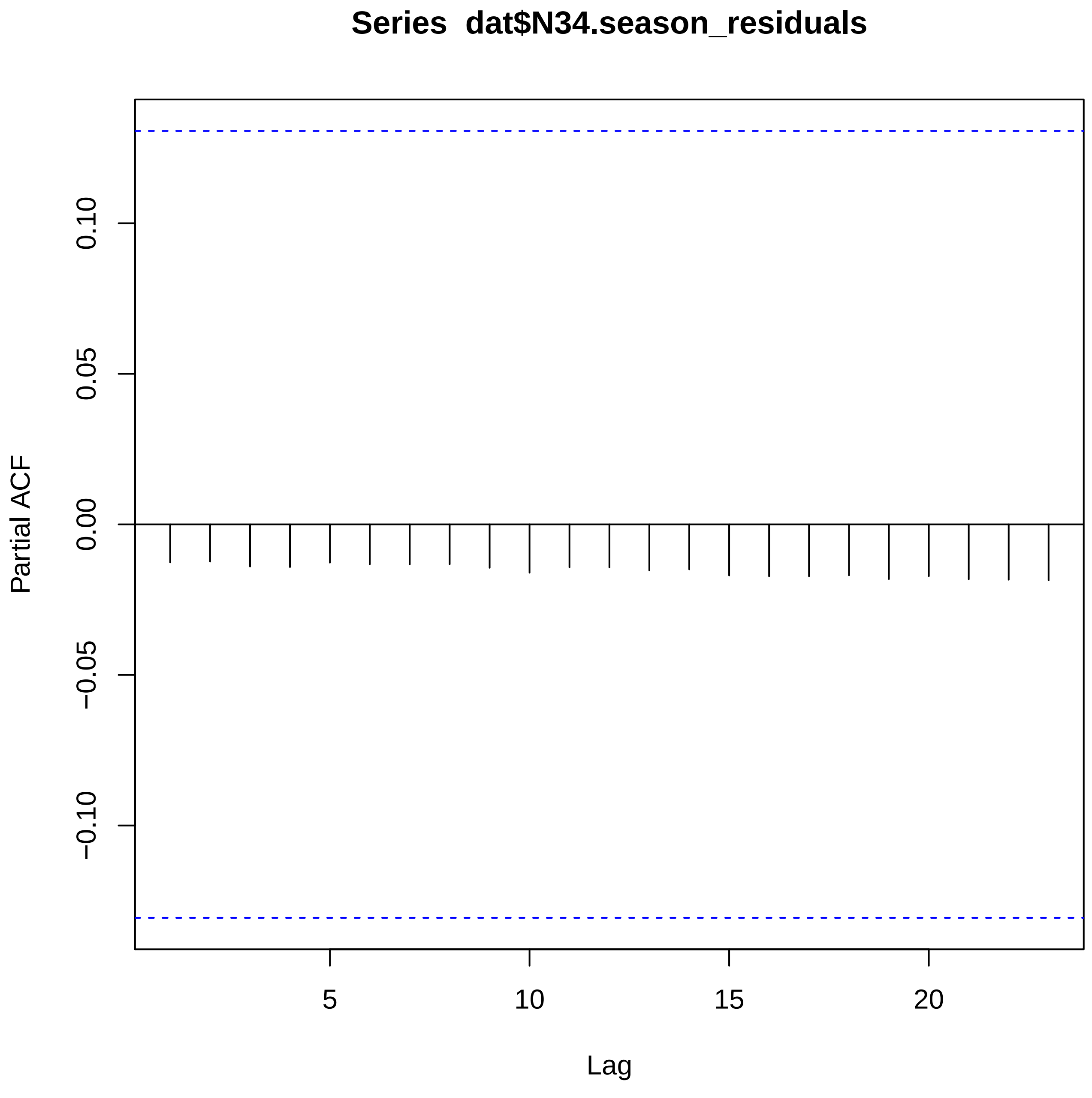

Model 6: Niño ONI with season interaction. Ubigeo 120608

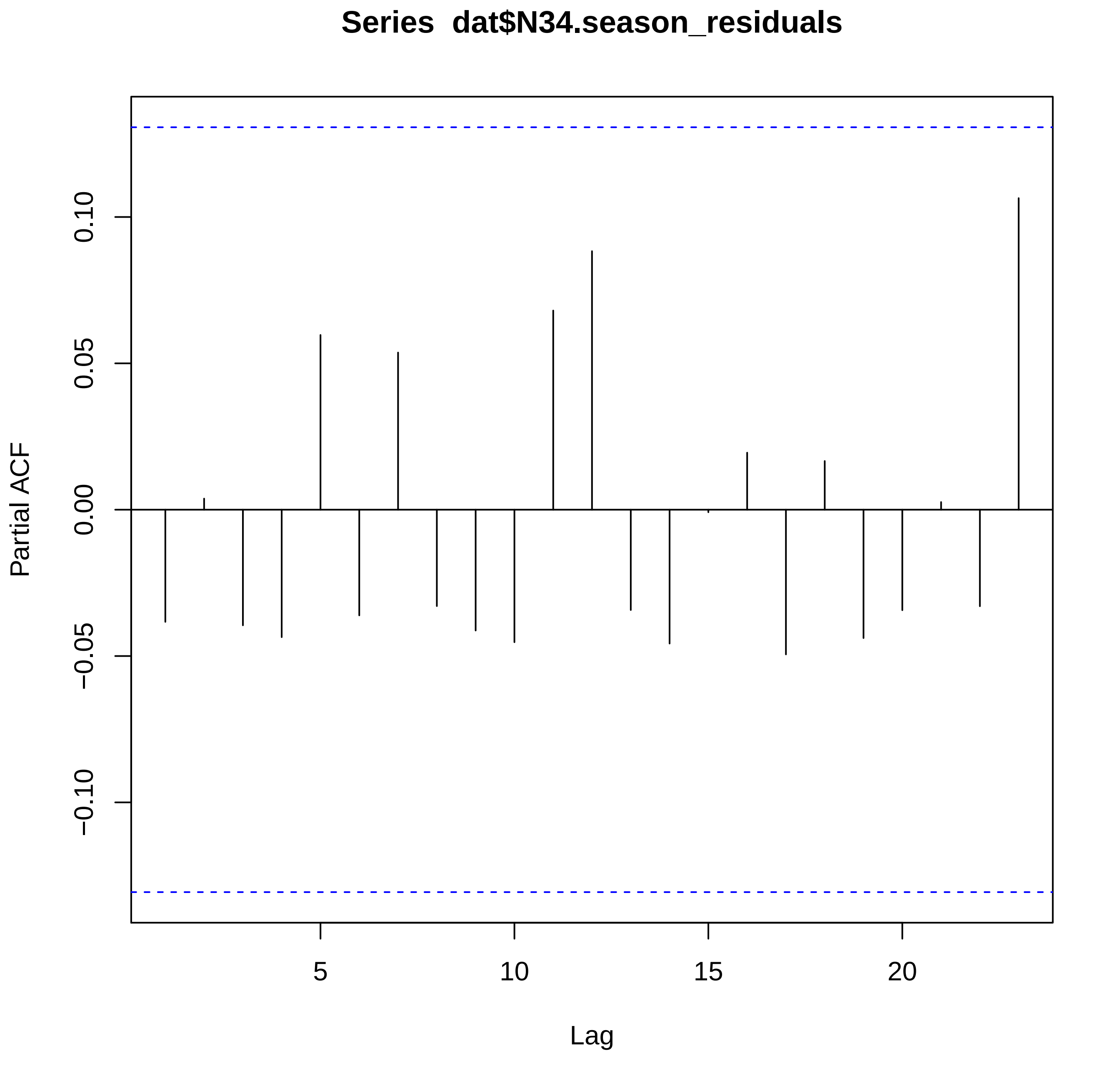

Model 6: Niño ONI with season interaction. Ubigeo 240103

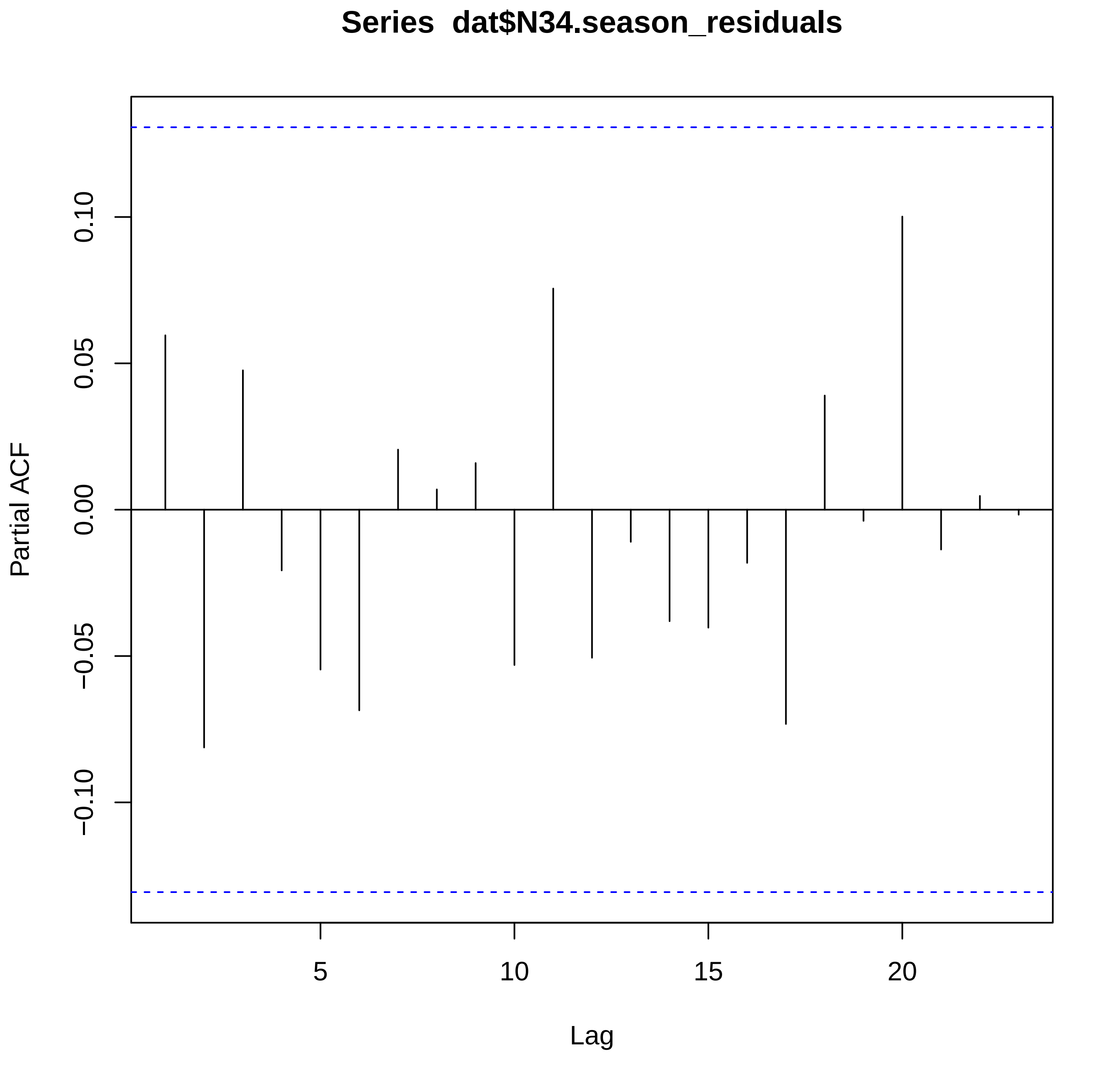

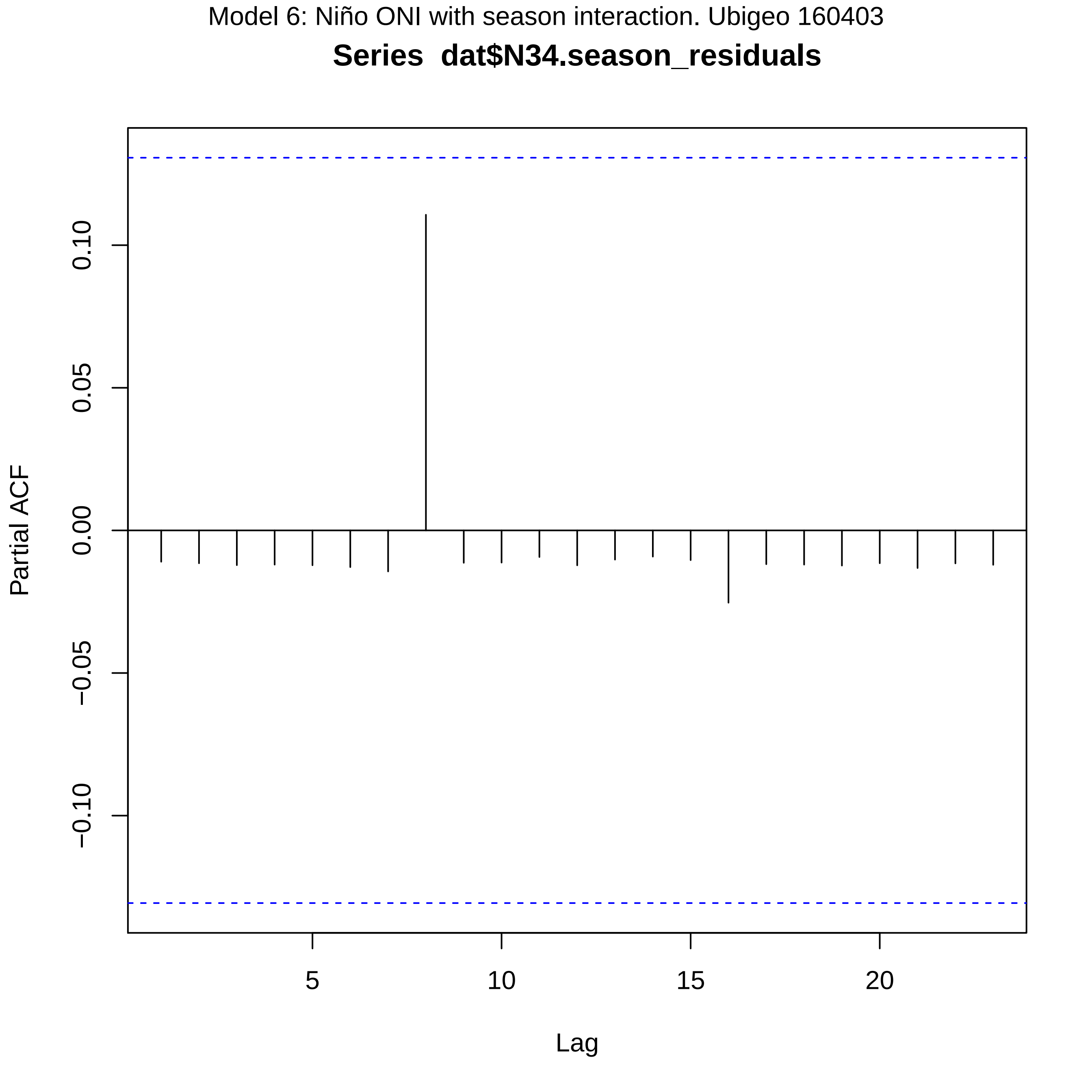
Model 6: Niño ONI with season interaction. Ubigeo 160403
Series dat$N34.season_residuals
Partial ACF
0.10
0.05
0.00
−0.05
−0.10
5
10
15
20
Lag

Model 6: Niño ONI with season interaction. Ubigeo 120302

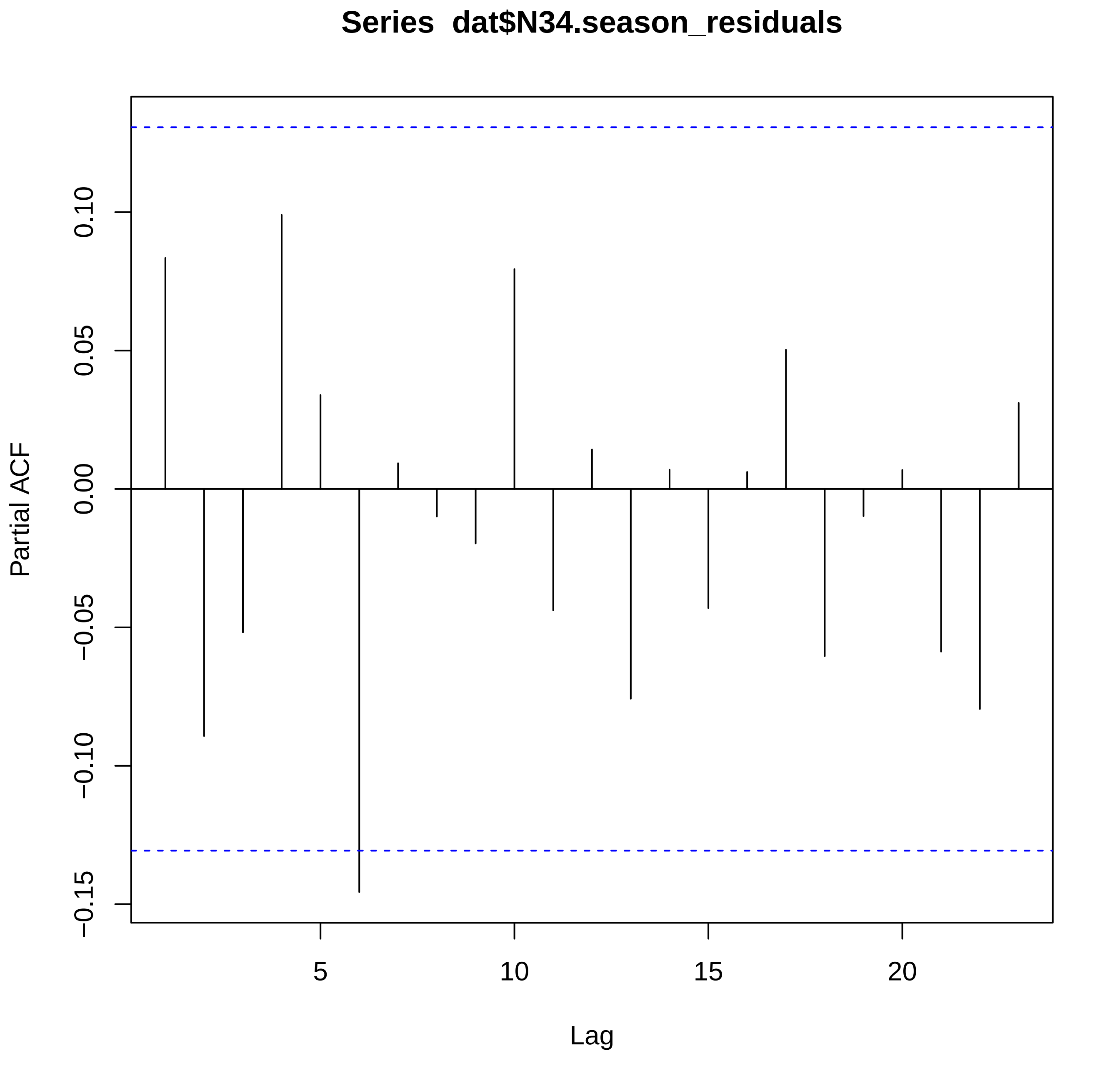

Model 7: Niño ICEN with season interaction. Ubigeo 220801

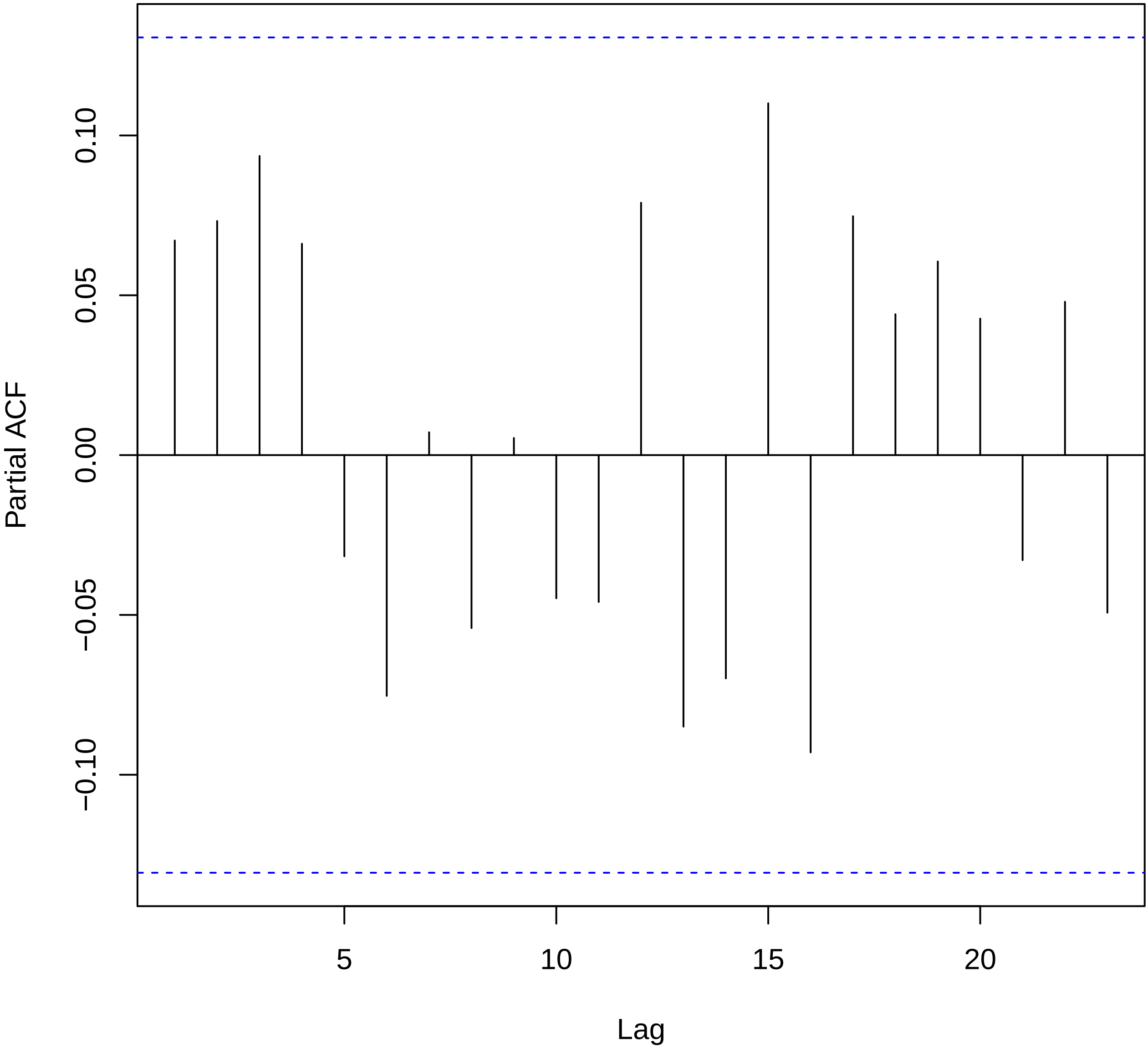

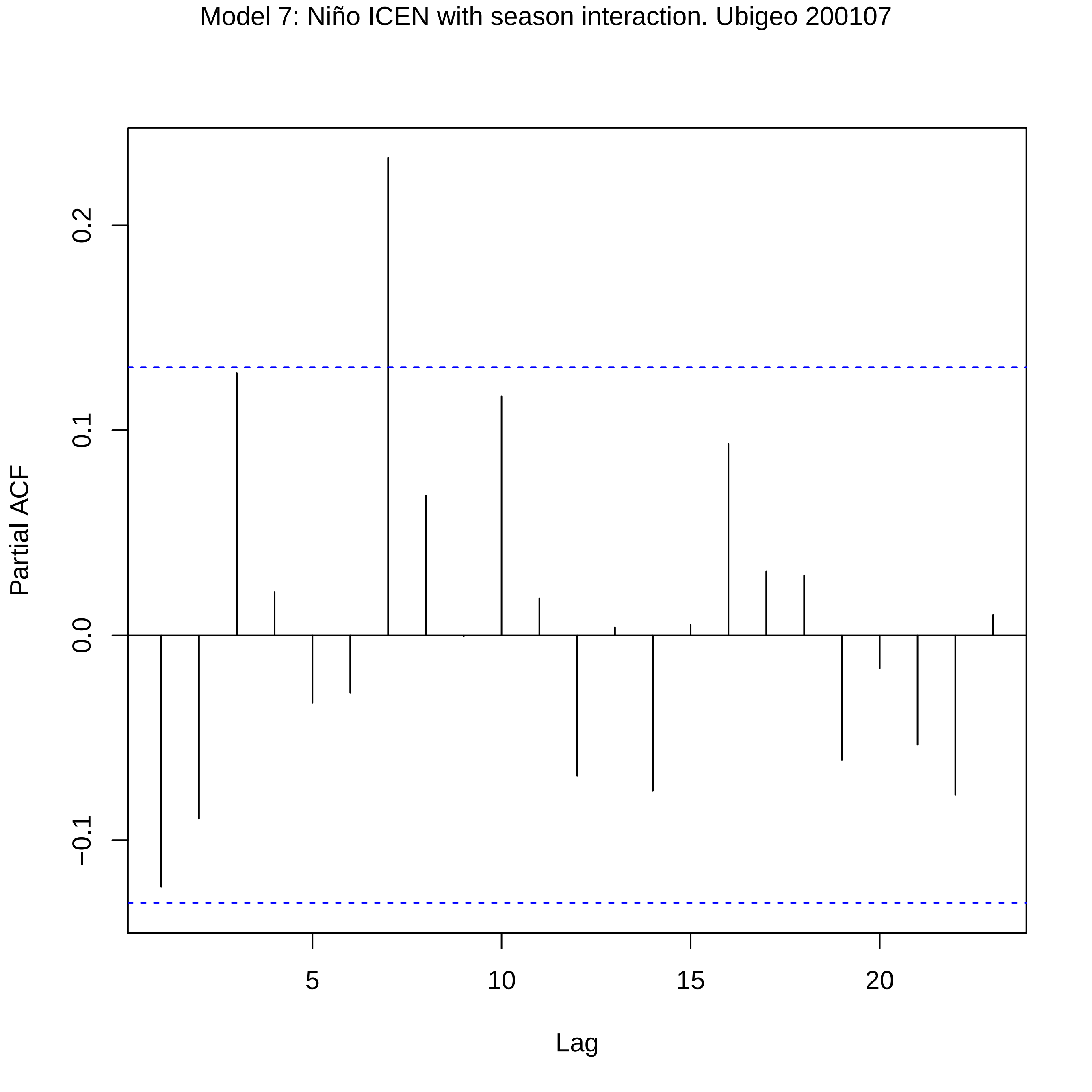
Model 7: Niño ICEN with season interaction. Ubigeo 200107
Partial ACF
0.2
0.1
0.0
−0.1
5
10
15
20
Lag

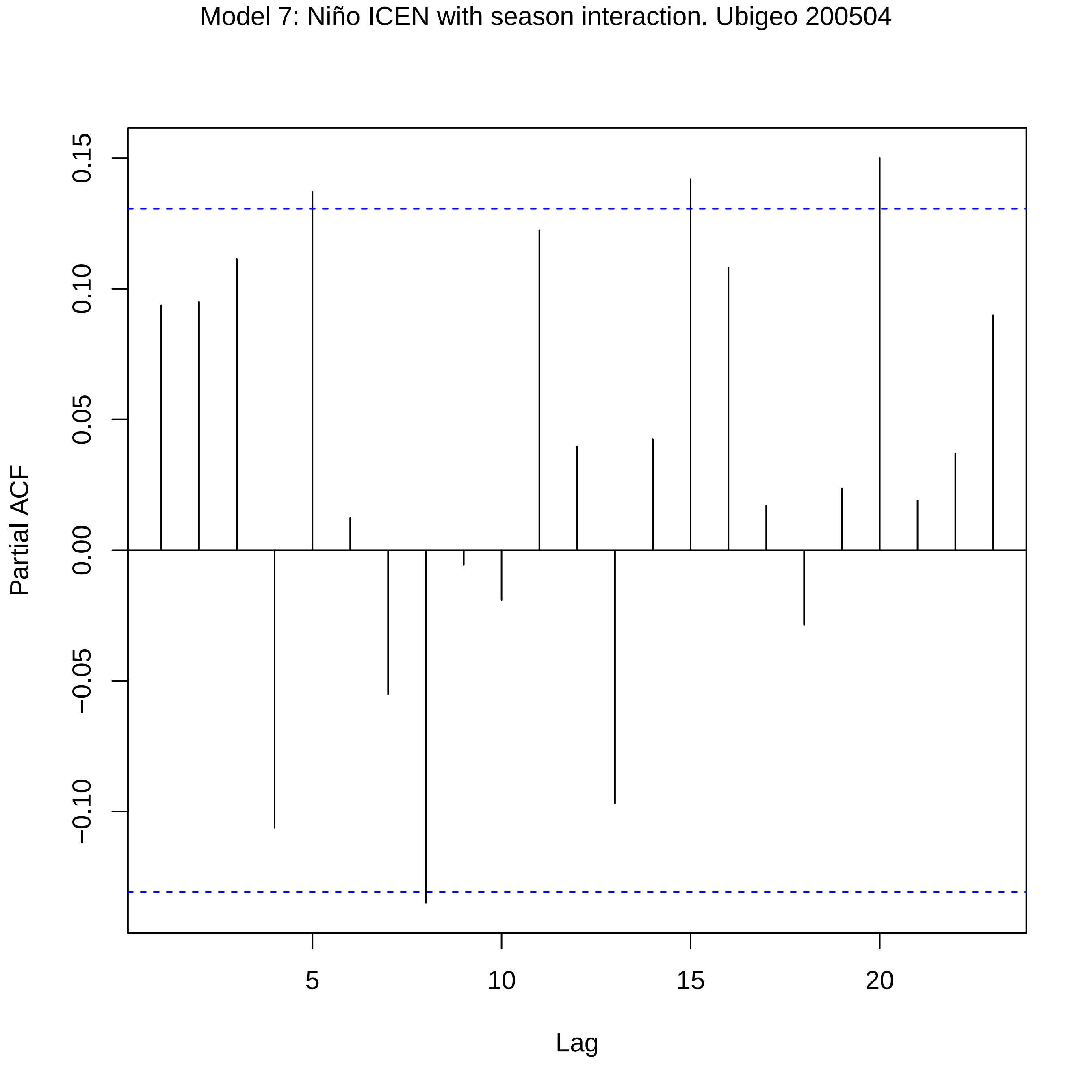
Model 7: Niño ICEN with season interaction. Ubigeo 200504
Partial ACF
0.15
0.10
0.05
0.00
−0.05
−0.10
5
10
15
20
Lag

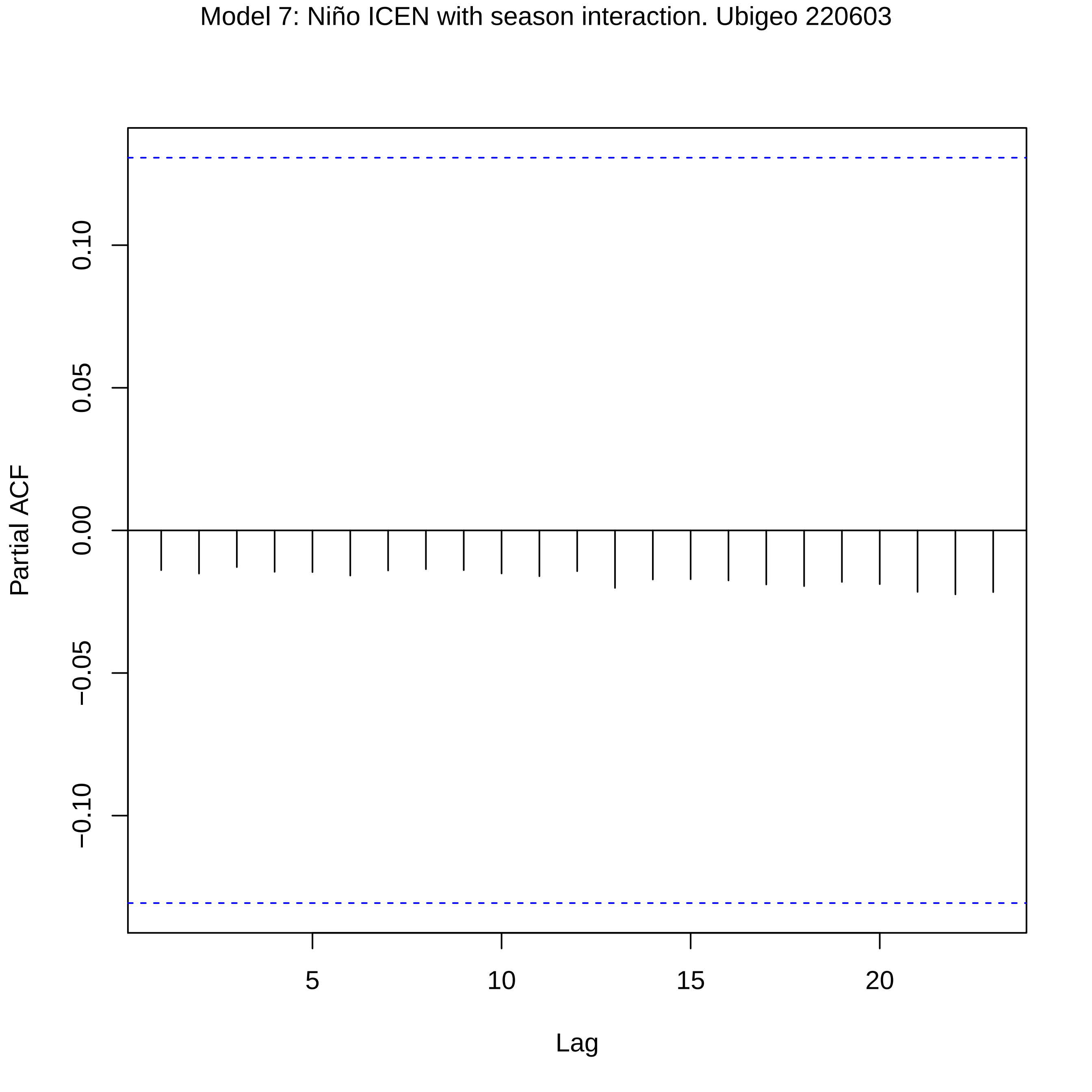

Model 7: Niño ICEN with season interaction. Ubigeo 220603
Partial ACF
0.10
0.05
0.00
−0.05
−0.10
5
10
15
20
Lag

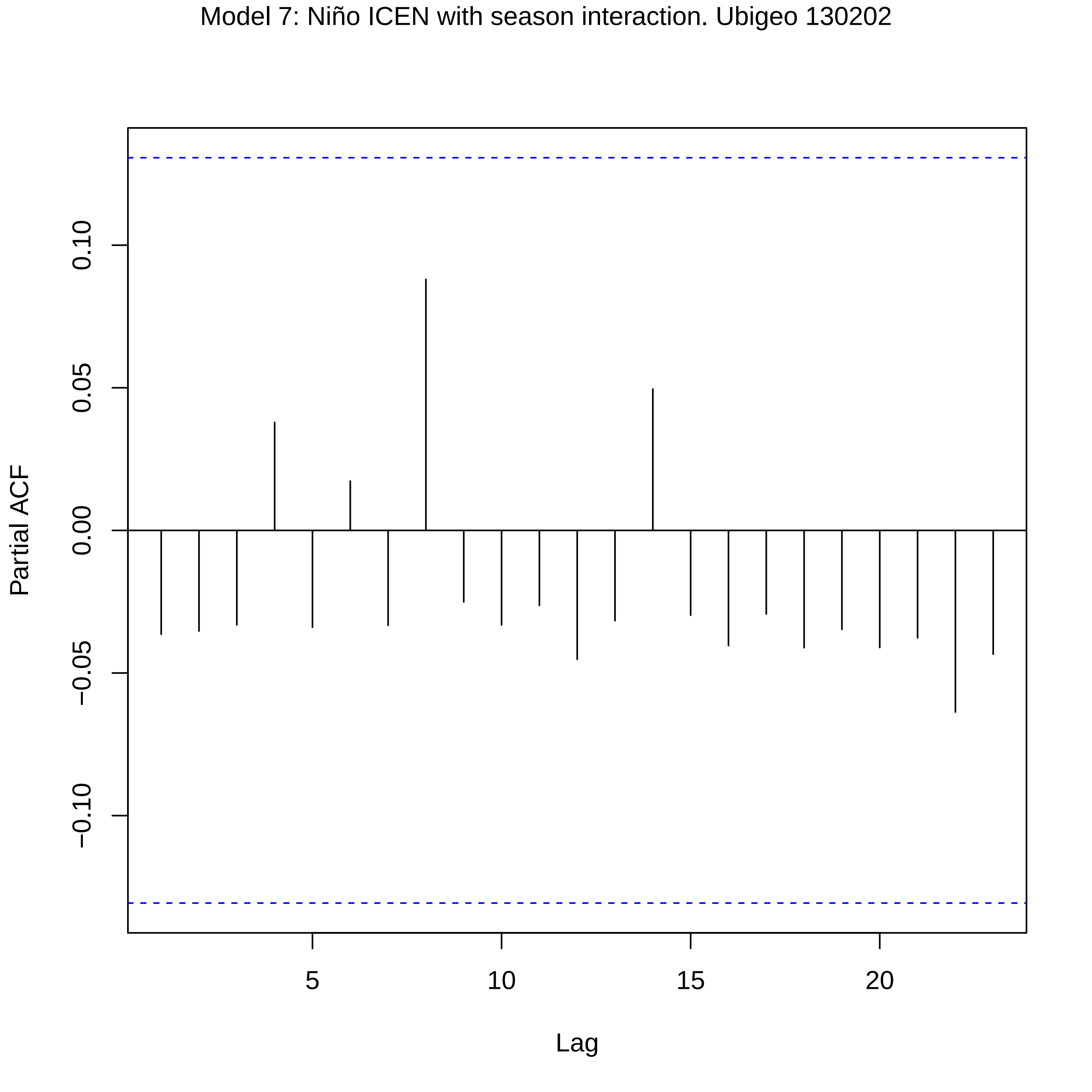
Model 7: Niño ICEN with season interaction. Ubigeo 130202
Partial ACF
Lag
0.10
0.05
0.00
−0.05
−0.10
5
10
15
20

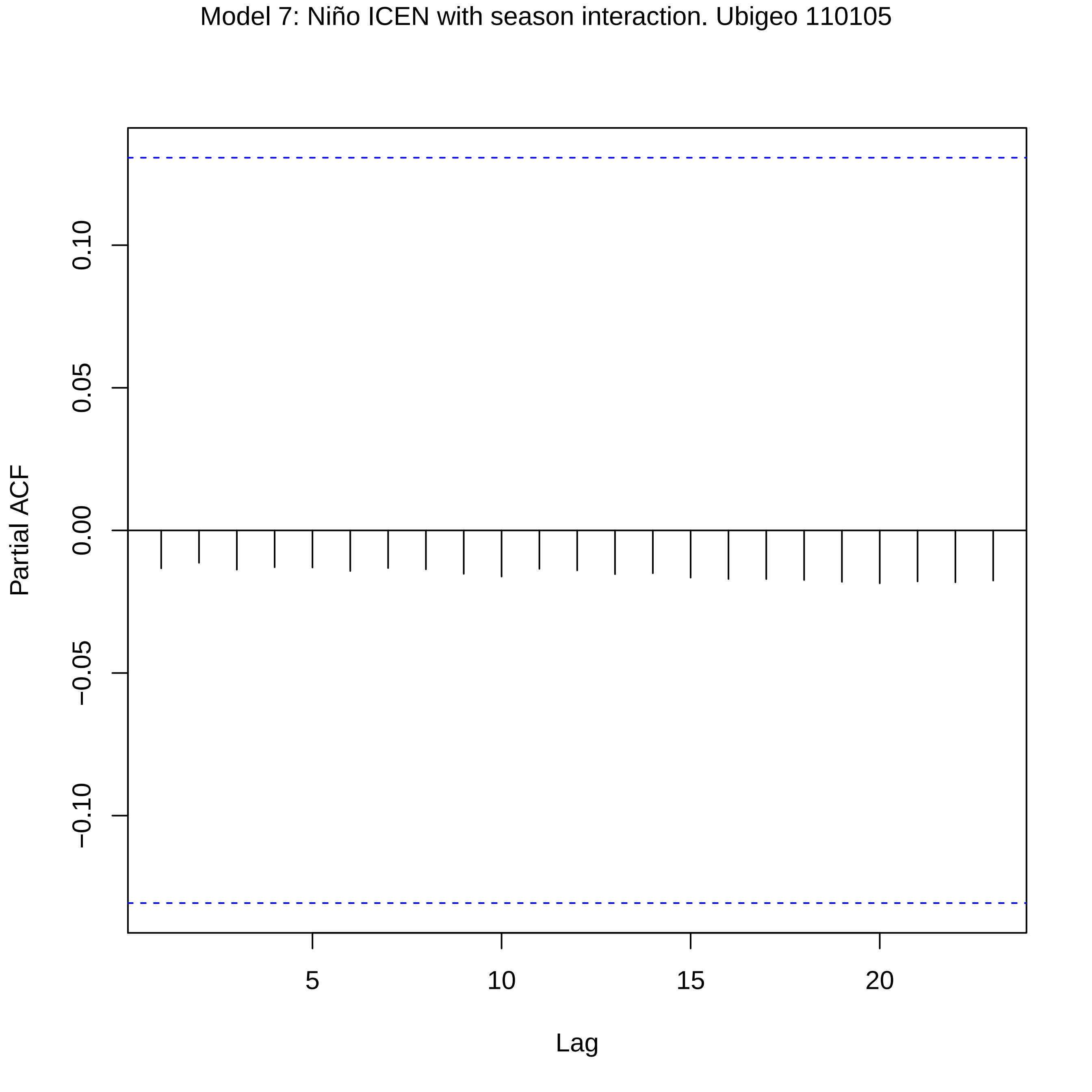
Model 7: Niño ICEN with season interaction. Ubigeo 110105
Partial ACF
0.10
0.05
0.00
−0.05
−0.10
5
10
15
20
Lag

Model 7: Niño ICEN with season interaction. Ubigeo 120608

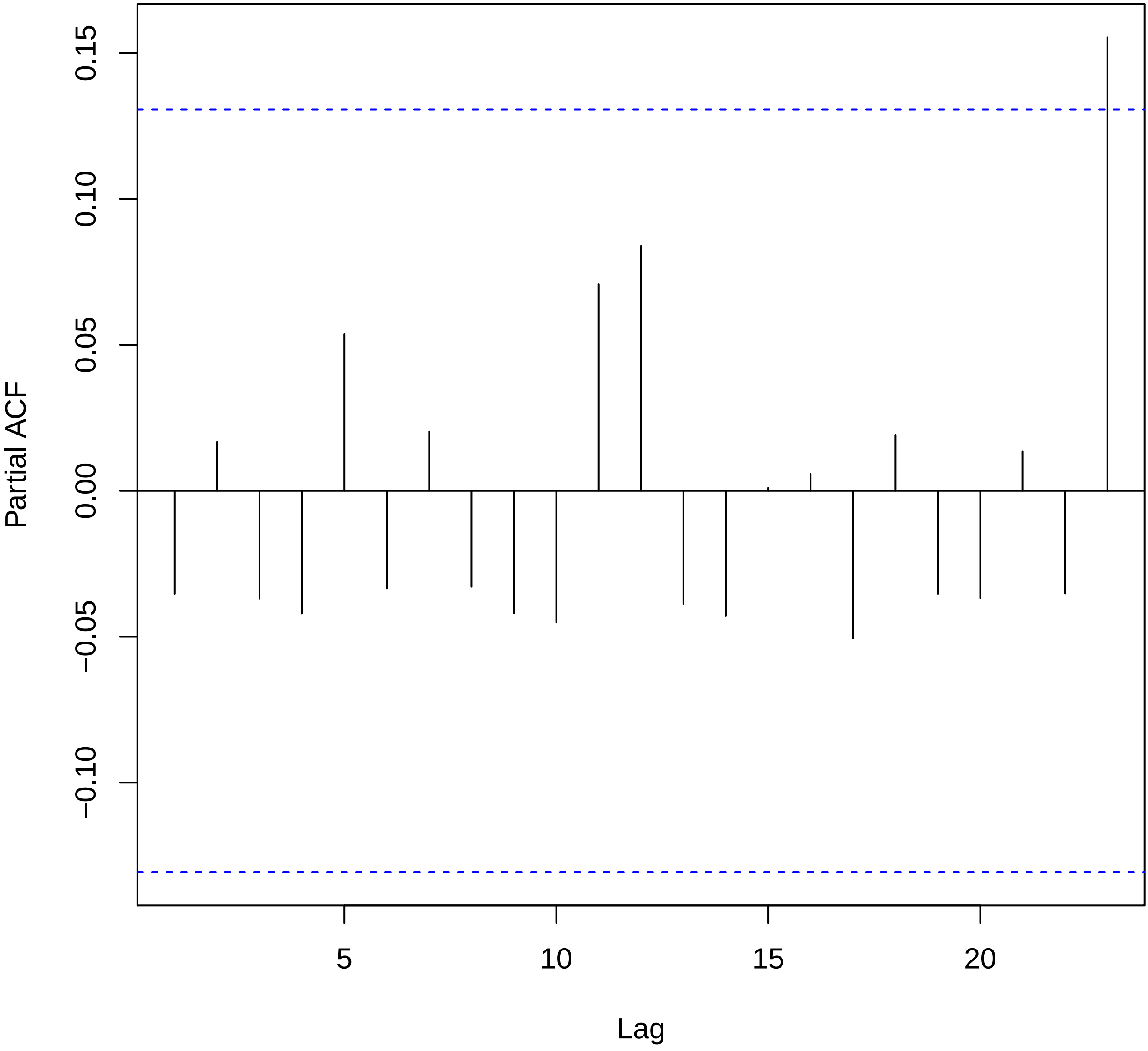

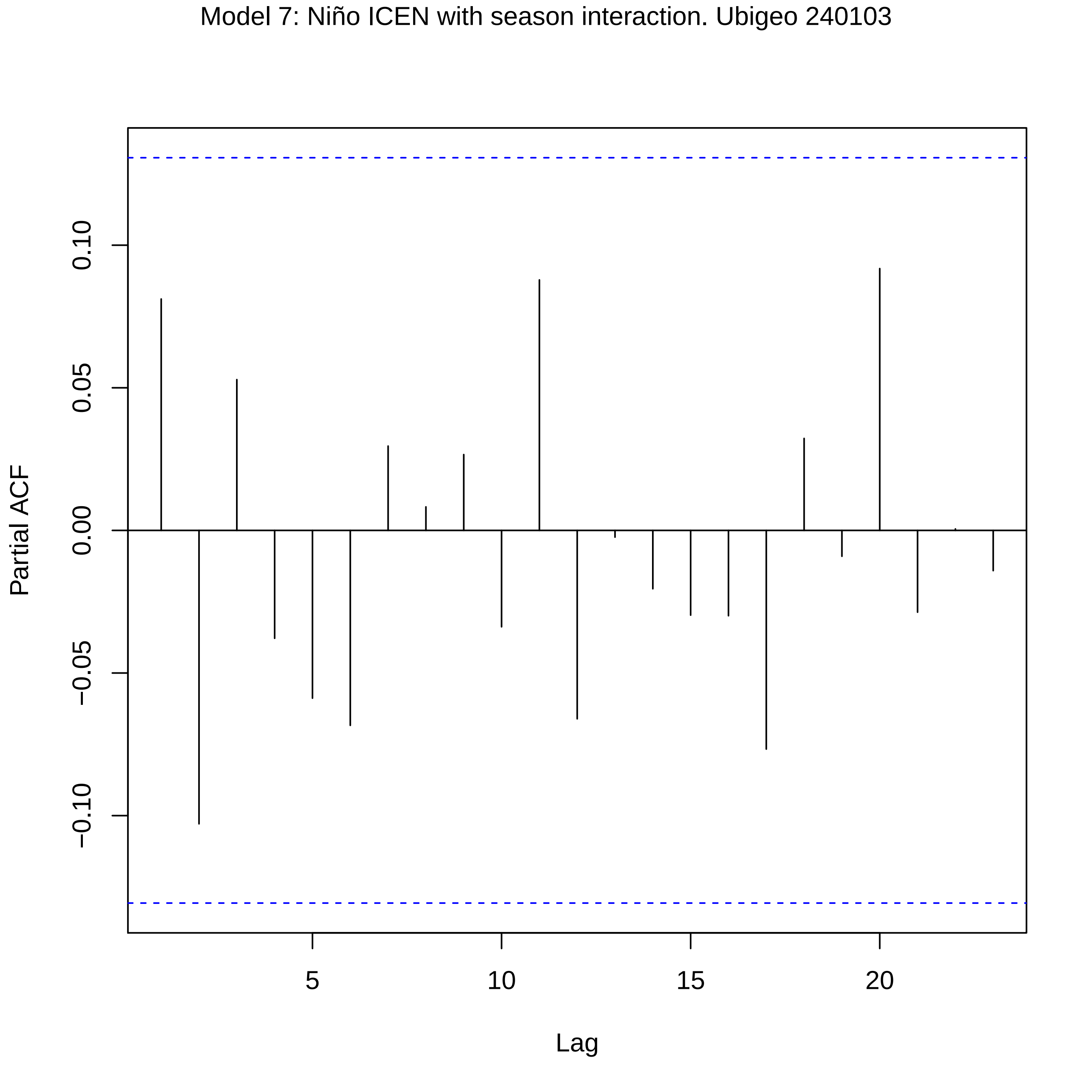

Model 7: Niño ICEN with season interaction. Ubigeo 240103
Partial ACF
0.10
0.05
0.00
−0.05
−0.10
5
10
15
20
Lag

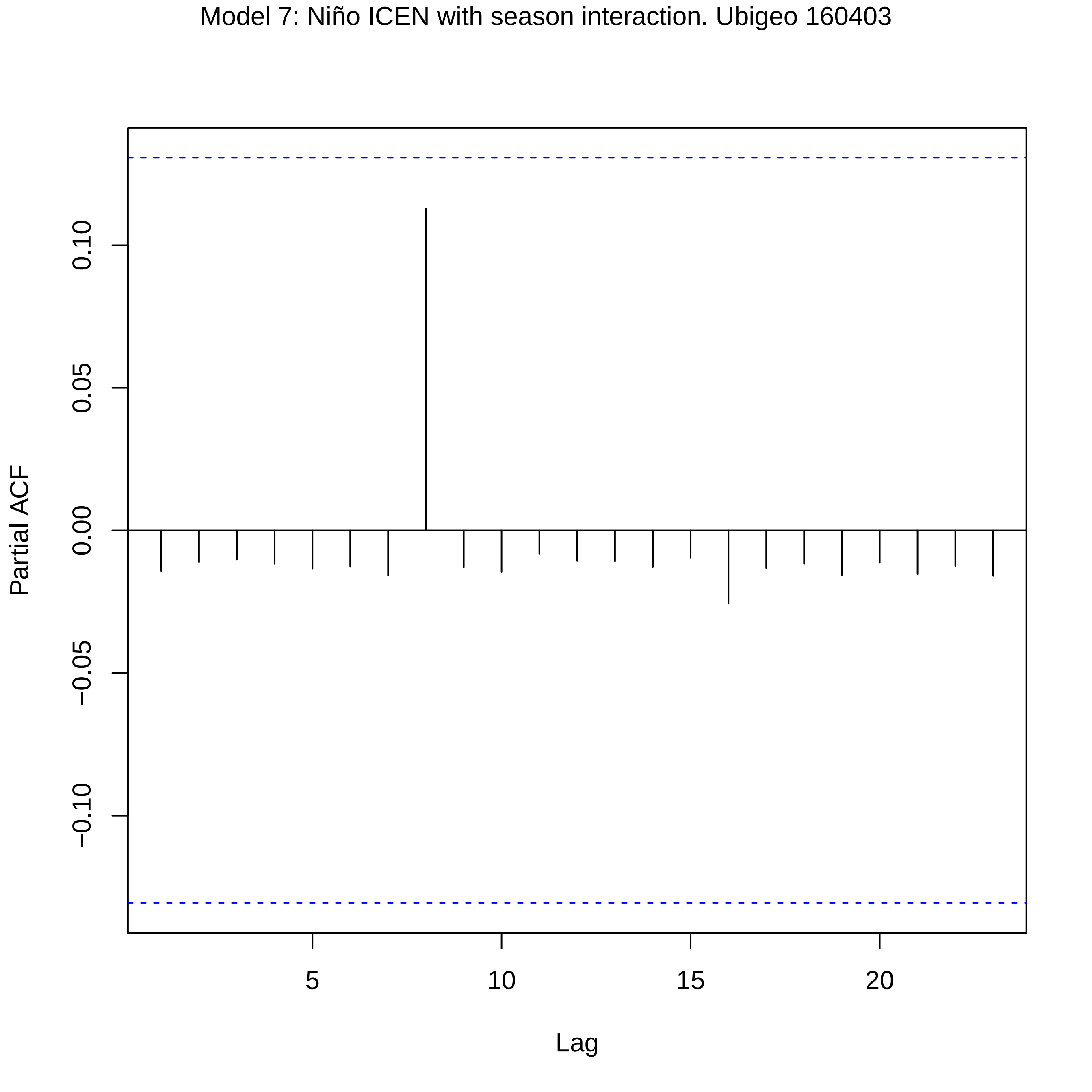

Model 7: Niño ICEN with season interaction. Ubigeo 160403
Partial ACF
0.10
0.05
0.00
−0.05
−0.10
5
10
15
20
Lag

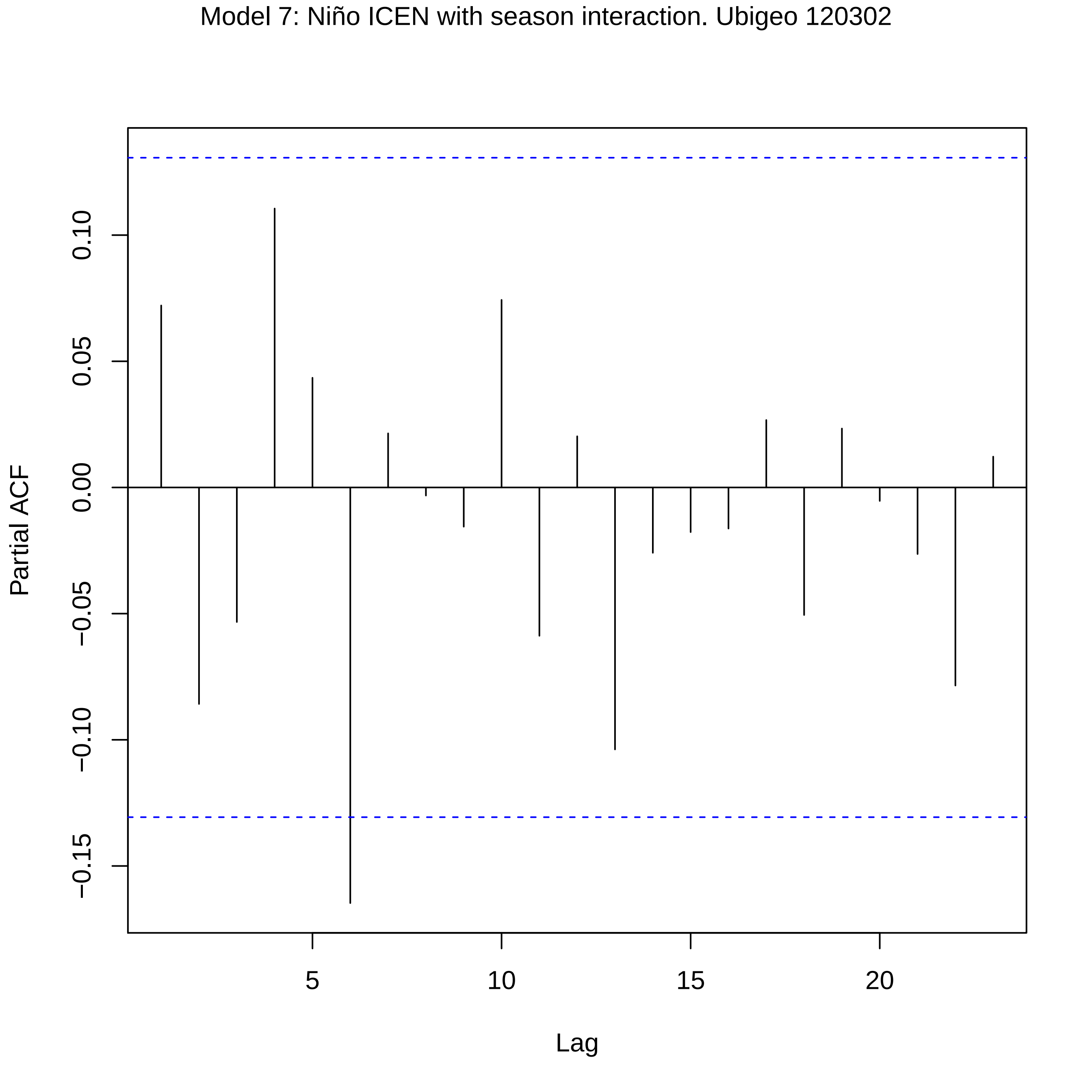
Model 7: Niño ICEN with season interaction. Ubigeo 120302
Partial ACF
0.10
0.05
0.00
−0.05
−0.10
−0.15
5
10
15
20
Lag
